# Oral Vaccination Using a Probiotic Vaccine Platform Combined with Prebiotics Impacts Immune Response and the Microbiome

**DOI:** 10.3390/vaccines10091465

**Published:** 2022-09-04

**Authors:** Bridget E. Fox, Allison C. Vilander, Darby Gilfillan, Gregg A. Dean, Zaid Abdo

**Affiliations:** Department of Microbiology, Immunology, and Pathology, Colorado State University, Fort Collins, CA 80523, USA

**Keywords:** *Lactobacillus acidophilus*, oral vaccine, IgA-seq, microbiome

## Abstract

Unique to mucosal vaccination is the reciprocal influence of the microbiome and mucosal immune responses, where the immune system is constantly balancing between the clearance of pathogens and the tolerance of self-antigen, food, and the microbiota. Secretory IgA plays a major role in maintaining the homeostasis of a healthy gut microbiome. Natural polyreactive IgA often coats members of the commensal microbiota to aid in their colonization, while high-affinity specific IgA binds to pathogens resulting in their clearance. We developed a probiotic-based mucosal vaccination platform using the bacterium *Lactobacillus acidophilus* (rLA) with the potential to influence this balance in the IgA coating. In this study, we sought to determine whether repeated administration of rLA alters the host intestinal microbial community due to the immune response against the rLA vaccine. To address this, IgA-seq was employed to characterize shifts in IgA-bound bacterial populations. Additionally, we determined whether using rice bran as a prebiotic would influence the immunogenicity of the vaccine and/or IgA-bound bacterial populations. Our results show that the prebiotic influenced the kinetics of rLA antibody induction and that the rLA platform did not cause lasting disturbances to the microbiome.

## 1. Introduction

Vaccination via mucosal routes is attractive because it provides both mucosal and systemic immunity, whereas parenterally delivered vaccines often do not provide protection at mucosal surfaces [1]. Lymphocytes exposed to antigen at one mucosal site can migrate to other mucosal sites, thus providing widespread protection against a pathogen [2]. These advantages come with a set of challenges. The inherent physical defense mechanisms of the gastrointestinal tract, such as the low pH of gastric acid, digestive enzymes, and the antimicrobial peptide-rich mucus, prevent many antigens from reaching immune inductive sites [3]. To overcome these obstacles, we developed a probiotic-based mucosal vaccination platform that utilizes the bacterium *Lactobacillus acidophilus*. *L. acidophilus* is bile-acid tolerant, allowing the bacterium to survive throughout the gastrointestinal tract [4,5]. *L. acidophilus* expresses natural ligands for TLR2 (peptidoglycans and lipoteichoic acid), NOD2 (muramyl dipeptide), and C-type lectin receptors (surface layer proteins) [6,7,8]. Oral vaccination with *L. acidophilus* provides an attractive and easy delivery system with several logistic benefits: cold-chain is not required for distribution or storage, medical training is not required for the needleless administration, and large-scale production is inexpensive [9,10]. These characteristics are particularly critical in low- and middle-income countries. We have constructed several recombinant rLA vaccine strains and characterized their immunogenic properties [5,11,12]. Recombinant *L*. *acidophilus* (rLA) strains can achieve massive surface expression of selected antigens when embedded in the surface layer protein A (slpA) [11]. Furthermore, adjuvants can be added to these rLA constructs, enhancing the immune responses and avoiding the induction of tolerance [13]. Thus, this rLA vaccine platform could be useful against a variety of important pathogens of humans and animals including rotavirus, coronaviruses, influenza, and HIV-1.

It is known that secretory IgA plays a major role in maintaining the homeostasis of a healthy gut microbiome [14,15,16]. Several studies have revealed that natural polyreactive IgA often coats members of the commensal microbiome population to aid in colonization, while high-affinity, antigen-specific IgA binds to pathogens resulting in clearance [17,18,19]. The rLA platform poses the potential to influence this balance in IgA coating through its combined function as a probiotic and activation of high-affinity antibodies against the vaccine antigen through a T-cell dependent response. To date, whether oral immunization influences IgA-coating of the intestinal microbiota has not been investigated.

In this study, we used IgA-seq to characterize shifts in IgA-bound bacterial populations to determine whether repeated administration of rLA alters the host intestinal microbial community because of the host mucosal immune response against the rLA vaccine. We used rLA that expressed the membrane proximal external region (MPER) from human immunodeficiency virus type 1 (HIV-1) and soluble mouse IL-1β (referred to as GAD19), which has previously been shown to induce shifts in the microbiome [20]. Additionally, we determined whether using rice bran as a prebiotic would influence the immunogenicity of the rLA vaccine and/or IgA-bound bacterial populations. Our results show that the prebiotic influenced the kinetics of rLA antibody induction and that the rLA platform did not cause lasting disturbances to the microbiome. The prebiotic played a primary role in modifications to the microbiome, while the *L. acidophilus* vector had a greater intermediate impact on the IgA-bound microbiome.

## 2. Materials and Methods

### 2.1. Ethics Statement

This study was carried out in strict accordance with relevant guidelines and regulations including the ARRIVE guidelines (https://arriveguidelines.org, accessed on 13 May 2019), the Guide for the Care and Use of Laboratory Animals of the National Institutes of Health, and the Association for the Assessment of Laboratory Animal Care standard with approval from the Institutional Animal Care and Use Committee of Colorado State University (protocol number: 14-5332A). Animals were monitored daily for clinical signs of illness or stress and humanely euthanized at the study’s endpoint via carbon dioxide inhalation and thoracotomy. 

### 2.2. Experimental Design

**Figure 1:**
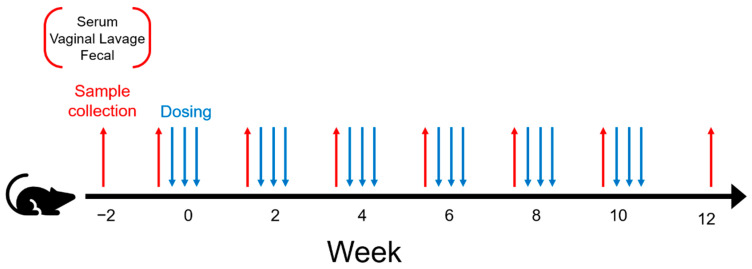
Experimental design for the vaccination schedule. Samples were collected from mice starting immediately prior to the change in diet at week 2. Mice were administered either NCK1895, GAD19, or the dosing buffer alone, as shown in Table 1. Samples collected prior to immunizations every two weeks included serum, vaginal lavage, and fecal samples.

Table 1 describes the experimental design associated with the current study. The table indicates a 3 × 2 experimental design with three vaccine treatments and two diets, resulting in 6 experimental groups. The three vaccine treatments used to orally dose mice included *L. acidophilus* expressing MPER and secreting IL-1β (GAD19), control strain *L. acidophilus* (NCK1895), and the carrier buffer alone (Buffer). For each of these treatment types, mice were fed either a standard chow diet (SC) or SC supplemented with 10% rice bran (RB). Eight mice were assigned to each experimental group, except Buffer_SC, where seven mice were used, as one of the mice died early in the study, for a total of forty-seven mice. All mice received the same number of treatment doses and were repeatedly measured biweekly over 8 time periods for immune response and changes in the microbiome as designated in Figure 1. Sampling associated with the immune response started two weeks prior to vaccination, allowing for sample collection just prior to starting mice on the rice bran diet. This resulted in a total number of 376 samples per type of immune response measured (i.e., fecal IgA, vaginal lavage IgA, and serum IgG). Sampling associated with the microbiome also started 2 weeks prior to vaccination and ended on week 12 resulting in a total of 376 samples per IgA-coating type (i.e., IgA positive, IgA negative, or whole microbiome), for a grand total of 1128 samples. 

### 2.3. Bacterial Strains and Culture Conditions

Wild-type *Lactobacillus acidophilus* strain NCK1895 [21] (harboring plasmid pTRK882) and GAD19 [5] (*L.* *acidophilus* strain NCK2208 with plasmid pGAD17) were grown in MRS broth (BD Diagnostics, Sparks, MD, USA) with 5 μg/mL of erythromycin (Em). Cultures were incubated overnight at 37 °C under static conditions. Expression of the membrane proximal external region (MPER), derived from human immunodeficiency virus type 1 (HIV-1) in the surface layer of our GAD19 strain, was confirmed using flow cytometry. Bacterial cells were first incubated with the anti-human immunodeficiency virus (HIV)-1 gp41 monoclonal antibody (2F5) in a 1% BSA PBS buffer and then incubated with goat anti-human IgG conjugated with Alexa Fluor 488 (Biolegend, San Diego, CA, USA). NCK1895 showed minimal background fluorescence, while GAD19 had high (>90%) fluorescence. Data were analyzed in FlowJo version 10.4 and gated on forward scatter (FSC), side scatter (SSC) to eliminate doublets and debris, and then FL1 to identify FITC positive events to indicate MPER-positive cells. Secretion of mouse IL-1β in GAD19 was confirmed using an ELISA-based detection kit.

### 2.4. Mouse Immunization and Housing

Female Balb/c mice from the Jackson Laboratory (Bar Harbor, Maine) were used, and all mice had access to ad libitum water and standard chow (Envigo, Teklad Rodent Diet) for two weeks at CSU’s Lab Animal Resources (LAR). After this two-week acclimatization period, mice were eight weeks of age at the start of the study, and half of the mice were switched to a Teklad custom 10% rice bran diet for the rest of the study (Envigo, Madison, WI, USA). Four mice per cage were housed in specific pathogen-free conditions with a continuous 12 h light/12 h dark cycle and were provided ad lib water and food throughout the study. Two weeks after changing to the rice bran diet, live bacterial vaccines were prepared using freshly grown overnight bacterial cultures for oral administration. NCK1895 and GAD19 bacterial cells were washed twice in PBS (Corning, Corning, NY, USA) and resuspended in a dosing buffer containing soybean trypsin inhibitor (STI, Sigma, Burlington, MA, USA) and sodium bicarbonate (NaHCO_3_). Mice were given 5 × 10^9^ CFU of either NCK1895 or GAD19 in 200 uL of dosing buffer or dosing buffer alone. Vaccines were delivered intragastrically three days in a row during weeks 0, 2, 4, 6, 8, and 10. Two weeks after the last dosing timepoint, the mice were euthanized, and the study was concluded.

### 2.5. Sample Collection and Processing

Blood, fecal, and vaginal samples were collected from each animal prior to administration of vaccination for investigation of antibody titers. Fecal samples were collected and homogenized with PBS supplemented with ProteaseArrest at a 10× weight-to-volume ratio. Homogenates were centrifuged at 9000 RCF for 10 min to pellet particulates and bacteria. Clear supernatants were aliquoted and stored at −80 °C for long-term storage. Fecal samples for microbiome analysis were collected directly from the anus of the animal into a sterile PCR tube and placed immediately on ice and transferred to −80 °C freezer for long-term storage. Serum samples were collected via tail bleeds. Blood was collected with a microvette (Sarstedt, Nümbrecht, Germany) and processed according to the manufacturer’s protocols for serum isolation. Serum was aliquoted and stored at −80 °C. Vaginal lavage samples were collected by gently washing the vagina of the mice with 100 ul of PBS. The collected sample were immediately placed on ice, centrifuged at 9000 RCF to pellet any debris, and then aliquoted and stored at −80 °C. 

### 2.6. ELISA Assays

An enzyme-linked immunosorbent assay (ELISA) was developed for the detection of MPER-specific and SlpA-specific murine antibodies from serum, fecal, and vaginal samples. Plates (Maxisorp; Nunc, Rochester, NY, USA) were coated with either MPER peptide (Bio-Synthesis Inc., Lewisville, TX, USA) at 1 ug/mL or SlpA protein (isolated from NCK1895) in carbonate coating buffer and incubated overnight at 4 °C. Plates were washed five times with PBS containing 0.05% Tween-20 (PBST) and blocked with 1% bovine serum albumin (BSA) in PBS for one hour at room temperature (RT). Plates were washed five times again with PBST. Samples were serially diluted in 1% BSA, 0.1% Kathon in PBS, and incubated for 2 h at RT. Plates were washed five times with PBST and incubated with either anti-mouse IgG (Cell Signaling Technology, 20 ng/mL) for the serum samples or IgA (Bethyl Laboratories, 40 ng/mL) for the vaginal wash and fecal samples. Both the anti-mouse IgG and IgA antibodies were conjugated with horseradish peroxidase (HRP) and incubated for 1 h at RT. Plates were washed six times with PBST. 3,3′,5,5′-tetramethylbenzidine (TMB) peroxidase (SeraCar, Milford, MA, USA) was filtered with a 40 uM syringe filter and acclimated to RT before adding to each well. The reaction was stopped with an equal volume of 1N HCl. The absorbance was read with a plate reader (BioTek, Winooski, VT, USA), with both 450 and 570 nm recorded (to remove any background noise with the 570 nm reading). The week 2 and 0 time points were used in the calculations of the mean baseline value for each group. This mean was then added to the standard deviation for each group times the standard deviation cutoff multiplier based on a within-experimental-group sample size of 8 and a 95% confidence level [22]. This value was used as the cutoff value to determine the reported endpoint titers used in the statistical analysis. 

### 2.7. Statistical Methods for ELISA Data

Statistical analyses were performed in R version 3.6.1. A Kruskal–Wallis test of the analysis of variance was used with Dunn’s multiple comparison post hoc test for each timepoint in the ELISA analysis, as the data were not normally distributed. Multiple testing was corrected using the Benjamini–Hochberg method to obtain adjusted *p*-values and are listed in Supplementary Materials Table S1. The endpoint titer means and standard error for each timepoint were plotted, with asterisks (*) representing significance (*p* < 0.05).

### 2.8. IgA-Bound Bacterial Sorting

The fecal pellets collected from animals at each timepoint were processed at the same time. Block randomization was used so that weekly timepoints and animal groups were evenly distributed among the plates to avoid batch bias in extraction, library generation, and sequencing. Additionally, samples were labeled with an assigned randomized number for all downstream work to blind investigators to the sample’s origin during library preparation and initial data processing. Fecal samples were first homogenized with PBS containing ProteaseArrest. Homogenates containing bacteria were placed on ice for 10 min to allow large particulates to fall out of the suspension. The remaining suspension was washed twice with PBS. One-third of the suspension was saved for direct microbiome processing (i.e., whole microbiome), while the remaining two-thirds were further processed for IgA sorting. Samples were stained with an IgG rat anti-mouse IgA antibody conjugated with FITC (Clone C10-3, BD Biosciences, San Jose, CA, USA) in a PBS staining buffer containing 0.01% bovine serum albumin (Sigma-Aldrich, St. Louis, MO, USA) and 1 mM EDTA. Samples were washed with staining buffer and filtered using 40 uM filter cap Falcon tubes before sorting with a FACS (fluorescence-activated cell sorter) Aria III at the CSU Flow Cytometry Facility. Sorting gates were set using both unstained samples and an isotype control with FACSDiva version 6.1.3 and FlowJo version 10.4. The side scatter (SSC) threshold was set to 250 to account for the small bacterial cells, and a 100 uM nozzle was used. Fresh CS&T beads (BD Biosciences) were run each day to ensure the machine was consistent in sensitivity detection throughout the sorting process. The gating strategy for sorting included selecting for intact cells based on FCS and SSC, followed by single cells to avoid clumps of heterogenous bacterial cells, and then gated on fluorescence in the FL-1 FITC channel to sort IgA-positive and IgA-negative cells (Supplementary Materials Figure S1). Samples from droplet stream were collected before and after sorting for negative controls. Several sorted samples were resorted to assess the accuracy of the IgA-positive and IgA-negative streams, with the purity ranging from 95 to 100%. Both IgA-positive and IgA-negative fractions were collected into sterile tubes and saved for 16S-library preparation. Sorting parameters were set to collect at least 100,000 cells in each fraction and stopped after 1 million cells were collected in either fraction so the collection tubes would not overflow. Samples were stored on ice throughout the sorting process until they were pelleted and frozen at −80 °C for later DNA extraction.

### 2.9. Microbiome Library Preparation

Each of the three fractions (i.e., whole microbiome, IgA positive, and IgA negative) were randomized into a 96-well plate format. Each plate received either three or six controls, which included no-template controls from either FACS sorting, extractions, PCR, or magnetic separation to track any contamination. Positive controls included ZymoBIOMICS Microbial Community Standards in the extraction (bacterial cells D6300) and in PCR (DNA D3605). DNA was extracted using the MagAttract PowerSoil DNA KF Kit (Qiagen) in conjugation with the KingFisher Flex (ThermoFisher Scientific, Waltham, MA, USA). Extracted DNA was used to create Illumina library molecules from the hyper variable region 4 (V4) of the 16S rDNA gene as described previously [20]. Briefly, primers included multiplexed barcodes, an Illumina adapter, pad and linker, and the V4-16S primer sequence. Dual-indexed libraries were purified using magnetic Mag-Bind TotalPure NGS beads (Omega Bio-Tek, Norcross, GA, USA) to select for DNA fragments greater than 300 bp to remove primers and any other unwanted PCR products. Library molecules in each sample were estimated using the AccuBlue dsDNA Quantitation Kit (Biotim). An equimolar amount of each sample was added to one of four pools. Each of the four pools were quality controlled and sequenced on an Illumina MiSeq at Colorado State University’s Next-Generation Sequencing Core Facility (Fort Collins, CO, USA). Illumina MiSeq v2500-cycle kits were used to sequence the 2 × 250 paired end reads. A dataset containing the raw reads is available in the National Center for Biotechnology Information’s (NCBI) Sequence Read Archive (SRA) under BioProject PRJNA723356 (https://www.ncbi.nlm.nih.gov/bioproject/PRJNA723356, accessed 20 April 2021).

### 2.10. Data Processing

We used the software fastqc [23] (version v0.11.5) to evaluate the quality of the demultiplexed fastq reads obtained from the MiSeq runs, totaling 56,460,071 reads. The software Trimmomatic [24] (version 0.39) was used to filter and trim the data using a sliding window of four and a cutoff quality of PHRED 25 in order to select for high-quality reads for downstream analysis. Only reads 150 base pairs or longer were used to ensure overlap between forward and reverse reads for assembling contigs in downstream processing, resulting in 25,387,655 reads. Filtered data were processed using mothur [25] (version 1.44.2), using the developers’ standard operating procedure (SOP) to further clean and process files, resulting in 24,748,343 sequences with 848,893 being unique. The output of this pipeline included an OTU-based data table and taxonomic classification. The SILVA database [26] (version 132) was used for alignment and classification. The microbial community standards (mock community) from ZymoBIOMICS were used to assess both the sequencing error rate and DNA extraction efficiency from different microbial taxa between extraction plates and sequencing batches. Negative controls were used to gauge the potential contamination introduced throughout the library preparation. These controls were also used to establish the cutoff for removing OTUs. Here, we removed OTUs with fewer than seven reads. Samples with fewer than 5000 reads were also removed. This cutoff also allowed for the convergence of nonmetric multidimensional scaling plots (NMDS). This process of data cleaning from the original mothur OTU table reduced the number of OTUs from 4567 to 417. Rarefaction curves were generated with the package vegan in R (version 3.6.1) to check that the depth of coverage for each sample allowed for the adequate discovery of OTUs (Supplementary Materials Figure S2). 

### 2.11. Diversity and Correlation Analysis

The alpha diversity was assessed using the Shannon diversity index and rarified richness. Shannon diversity was estimated using the phyloseq package [27] on non-normalized data. Richness was calculated based on the rarified data through the package vegan [28]. The R package lme4 was used to create a linear mixed-effects model to account for random effects from sampling the same mice over time, and the predicted values were used in the statistical analysis. This model specified individual mice as subject-specific random effects, and the experimental group as fixed variables. Autocorrelation was not used, as the AIC values between models with an autocorrelation component and those without were comparable (within 2 for all models). The predicted values were plotted as the means with standard error using the ggplot2 and sjPlot packages.

The beta diversity was analyzed via nonmetric multidimensional scaling (NMDS) at the OTU level after normalization using cumulative sum scaling [29]. The full dataset was used for ordination through the vegan package and by applying Bray–Curtis dissimilarity. The 95% confidence ellipsoids were plotted for each timepoint, group, and microbiome fractions, depending on the graph. Plotting was also conducted for each sequencing pool to identify possible batch bias. Venn diagrams were constructed using the VennDiagram package in R based on both the OTU and genus-level taxa counts. Data were separated either by the IgA fraction or the IgA fraction and treatment group to show the similarities and differences between the various groupings. 

Spearman’s correlation coefficient was used to assess the association between the microbiome and MPER-specific IgA. The top 20 taxa identified as the most important for classification into treatment groups in random forests (described below) were used in this correlation analysis. 

### 2.12. Random Forest

To create a list of taxa that had the most influence on the prediction of the treatment groups (Table 1) associated with vaccination and diet from the study, we used random forests (RFs) [30], utilizing the R package randomForest (version 4.6–14) [31]. Each IgA fraction was analyzed independently to determine the most important taxa for each population. The optimal number of features was identified with an iterative approach and used in constructing trees. We used the tuneRF function to iterate over 100 random forests with different mtry values, and ntreeTry was set to 200. The mtry value, representing the number of features to use in sampling when creating regression trees in the RF model, with the lowest median out-of-bag (OOB) error was selected (whole microbiome = 46; IgA-positive microbiome = 98; IgA-negative microbiome = 60) for the model to classify the experimental groups (Supplementary Materials Table S2). Additional models classified samples based on treatment and diet (Supplementary Materials Tables S3 and S4; OOB error rates as shown in Supplementary Materials Figure S3). We used ntree = 1000 and importance = TRUE to create the RF to identify features that were most important in classification to the experimental groups. All OTUs, sample collection time points (week), and sequencing pool were included in this analysis. The features and their importance are represented by the mean decrease in Gini [32], using a cutoff of 0.2 when plotting. Gini coefficients for all OTUs can be found in Supplementary Materials Tables S5–S7. The sequencing pool was included as a feature to assess any bias based on the sequencing batch, and no bias was detected. 

## 3. Results

### 3.1. Antigen-Specific Antibody Responses

**Figure 2:**
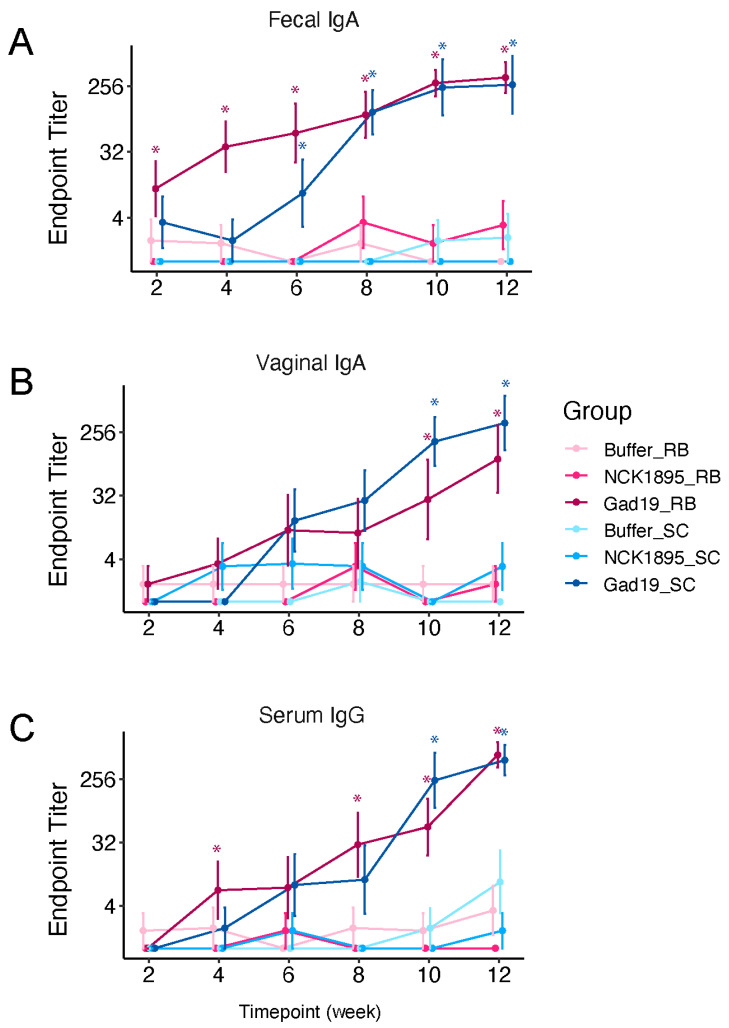
Diet improved antigen-specific antibody response to vaccination. Mice were fed either standard chow (SC) or SC supplemented with 10% rice bran (RB) throughout the study, and samples were taken every 2 weeks prior to the next vaccine boost. MPER-specific antibodies were detected with colorimetric ELISAs. (**A**) Fecal IgA, (**B**) vaginal IgA, and (**C**) serum IgG endpoint titers are reported for each group, with samples having no detection being assigned a value of 1. For all graphs, asterisks (*) indicate significance (*p* < 0.05) compared to the Buffer groups. Adjusted significant *p*-values are listed in Supplementary Materials Table S1 for all comparisons.

To investigate the kinetics and magnitude of the antigen-specific immune response induced by the recombinant *L. acidophilus* vaccine platform, mice were orally dosed with *L. acidophilus* expressing MPER and secreting IL-1β (GAD19), the control strain *L. acidophilus* (NCK1895), or the carrier buffer alone (Buffer). For each treatment type, mice were fed either a standard chow diet (SC) or SC supplemented with 10% rice bran (RB) (Table 1). MPER-specific IgA in feces and vaginal wash and serum IgG showed a pattern of increasing titers with each vaccine boost, reaching a maximum titer after the 6th immunization with GAD19 (Figure 2, adjusted *p*-values for pairwise comparisons shown in Supplementary Materials Table S1). Notably, mice fed the RB diet showed significantly higher antibody titers in feces and serum after two immunizations with GAD19 (week 4) compared to all other treatment groups (Figure 1 and Figure 2A). This trend continued for the GAD19_RB group, which had significantly higher titers of serum IgG after the 4th immunization as well (Figure 2C). These results show that the GAD19 vaccinated groups produced significant levels of MPER-specific antibodies in all three sample types (i.e., fecal, vaginal, and serum), but rice bran could improve early mucosal and systemic humoral responses to rLA vaccination.

### 3.2. Impact on Alpha Diversity

**Figure 3:**
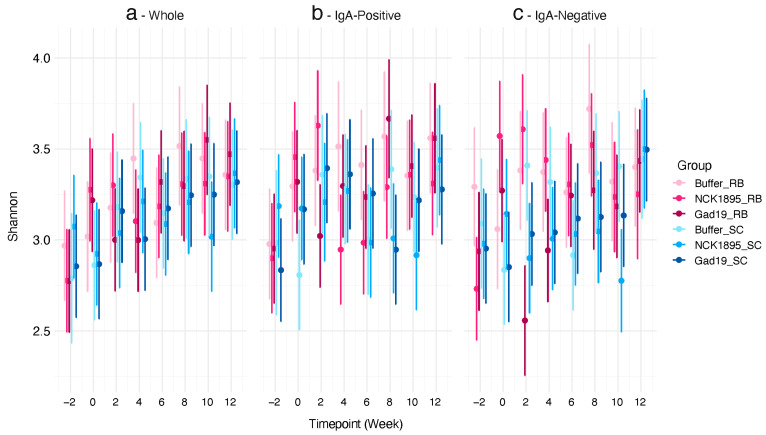
Alpha diversity was not affected by diet or vaccination: (**a**) predicted values of the Shannon diversity index for the whole microbiome from each timepoint (i.e., weeks 2, 0, 2, 4, 6, 8, 10, and 12) taken from each group are represented by the 95% credibility intervals; (**b**) IgA-positive and (**c**) IgA-negative microbiome fractions are also represented by the 95% credibility intervals. A linear mixed-effects model was used to determine the predicted values for each microbiome type (i.e., whole, IgA positive, and IgA negative).

We used linear mixed-effects (LME) models to fit both the Shannon diversity index and expected richness. Each model was fitted separately for the different microbiome fractions (i.e., IgA positive, IgA negative, and whole). Figure 3 shows the Shannon diversity indices for each microbiome fraction. Although no significant differences were observed between any of the timepoints or groups in the whole microbiome (Figure 3a), the trajectory of the means of the Shannon diversity for most groups increased overtime. There was less variability in the confidence intervals for the whole microbiome compared to the IgA-positive and IgA-negative populations (Figure 3b,c). The means and confidence intervals for both fractions did not follow a linear trend. Figure 3c indicates that post-vaccination (week 2), a significant impact on the Shannon diversity of the IgA-negative microbiome was associated with the RB treatment (GAD19_RB). However, these impacts recovered quickly after that time point.

The predicted values of expected richness also show a nonlinear trend over time for each microbiome fraction (see Supplementary Materials Figure S4). No significant differences were found between treatment groups at any timepoint or between IgA-positive and IgA-negative fractions. The sample collection timepoint, which occurred two-weeks post-vaccination, also provides time for recovery from a short-term perturbation caused by vaccination. Therefore, these results indicate that our live bacterial vaccine platform did not lead to lasting effects on the alpha diversity of the intestinal microbiome. 

### 3.3. Temporal Changes in Beta Diversity

**Figure 4:**
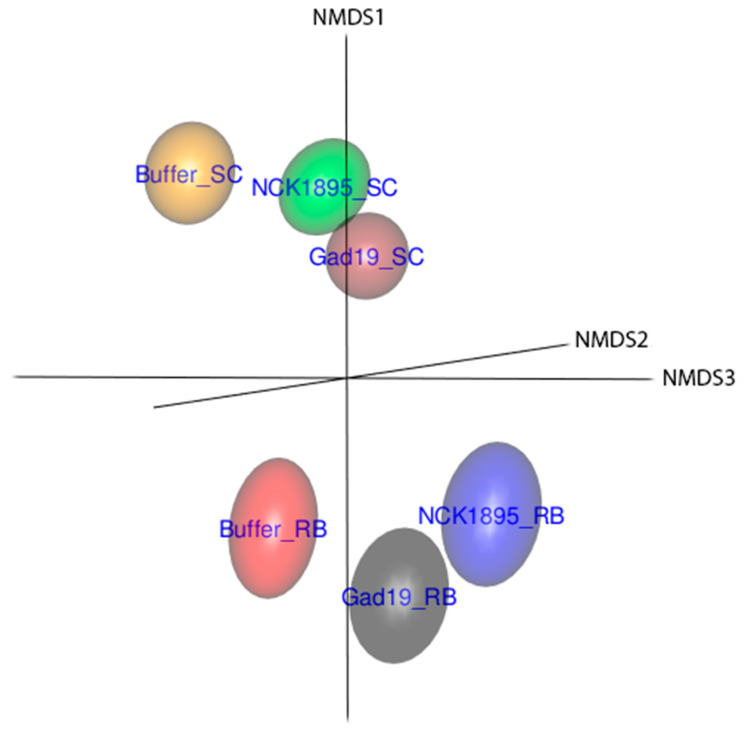
The three-dimensional (3D) nonmetric multidimensional scaling (NMDS) plot shows that the beta diversity separated samples by the experimental groups. The Bray–Curtis distance was used to determine differences in the microbiome community structure between groups. Data included all samples from all timepoint from each experimental group. Data are represented by the 95% confidence ellipsoids.

A clear separation of whole microbial communities based on diet is demonstrated in Figure 4. Beta diversity is presented based on the nonmetric multidimensional scaling (NMDS) ordination using the Bray–Curtis distance and utilizing the full dataset. The 3D representation of these data highlights the separation of the microbial communities based on diet and partially on immunization type, where the 95% confidence ellipsoids represent the community structure for each group from all three microbiome fractions. The slightly overlapping ellipsoids from GAD19_SC and NCK1895_SC represent a more similar microbiome. These treatment groups were administered the same species but different strains of *L. acidophilus*, indicating that shifts in the microbiome may be caused by the administration of the live *L. acidophilus* vector itself. The close proximity between GAD19_RB and NCK1895_RB also supports this possibility. 

Supplementary Materials Figure S5 reveals the temporal changes in the microbial community throughout the duration of the study. Differences in the microbial communities caused by diet alone were highlighted in the control buffer group (Supplementary Materials Figure S5A,B). The centroids for all IgA fractions in the Buffer_SC group shifted downwards overtime, with the microbiome seeming to stabilize between weeks 8 and 12. Conversely, the microbiome in the RB buffer group shifted from week 2 to 0 for all IgA fractions (Supplementary Materials Figure S5A), again emphasizing diet as a primary driver of changes to the microbiome. The whole microbiome samples for this group had two main clusters: one with the −2, 2, 4, and 6 week timepoints and another cluster containing the 0, 8, 10, and 12 week time points. A similar clustering pattern was seen in the IgA-negative and IgA-positive communities, but the overlapping ellipsoids at many of the timepoints indicated that the difference was not significant, whereas the differences were in the whole microbiome. 

A comparable pattern was seen in the NCK1895 groups, with the SC diet mice showing little change in community structure over time, while the RB diet group showed significant changes and similar clustering patterns (Supplementary Materials Figure S5C,D). The IgA-negative and IgA-positive microbiome fractions for the NCK1895 group on the SC diet showed no significant changes over time, while the only significant differences in the whole microbiome appeared between week 2 and 12 (Supplementary Materials Figure S5D). Animals on the RB diet in the NCK1895 group did show significant changes in the microbiome over time, but this can be attributed to the change in diet alone (Supplementary Materials Figure S5C). The whole microbiome community showed clusters between weeks 4, 6, and 8 as well as 0, 2, 10, and 12. Because the 0 time point overlapped with the 10 and 12 week timepoints, we concluded that the microbiome recovered to its state before probiotic treatment. These results indicate that repeated dosing with the probiotic bacterium *L. acidophilus* did not significantly alter the microbiome as a whole or the subset of the microbiome that is recognized by the mucosal immune system.

Mice immunized with the GAD19 strain, again, showed differences during the switch from the SC diet at week 2 to the RB diet at week 0 (Supplementary Materials Figure S5F). This shift was clearly seen in both the whole microbiome and IgA-positive microbiome. The centroids for each timepoint also followed a similar trajectory between the two sample types (i.e., whole microbiome and IgA positive) in the GAD19 RB group. Animals in the GAD19 and SC diet did not show a significant difference between the community structure at weeks 0 and 12 for any of the microbiome fractions (Supplementary Materials Figure S5E). The IgA-negative microbiome fraction for the GAD19 animals on both diets showed a similar pattern, as seen by the overlapping ellipsoids for the majority of the timepoints. The shifts in community structure earlier in the study showed that the perturbations caused by vaccination were resolved and returned to the starting homeostasis by the conclusion of the study, highlighted by the similarities in the week 0 and week 12 timepoints. 

These data show the large role that the prebiotic played in inducing changes to the microbiome. However, this impact was mainly observed in the whole microbiome samples and partially observed in the IgA-positive fractions. Additionally, the administration of our *L. acidophilus* strains did not induce long-lasting effects on the microbiome, as shown by the overlapping ellipsoids representing the community structure between early and late timepoints. The similarities across the IgA-negative fractions between treatment groups shows that neither the rLA vaccine nor RB prebiotic caused a major disturbance in the IgA-negative bacterial community. The similar temporal clustering patterns of the GAD19 and NCK1895 IgA-positive and whole microbiome samples also indicate that the intermediate shifts seen in the whole microbiome samples were attributed to changes in the IgA-positive bacterial communities.

### 3.4. IgA Fractions Uncovered Low-Abundant Taxa

**Figure 5:**
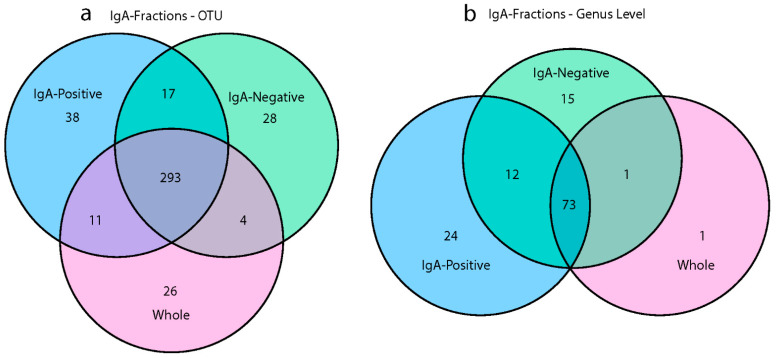
Shared and unique bacteria in different microbiome fractions. Venn diagrams represent the number of (**a**) OTUs or (**b**) genera found in the three microbiome types (i.e., whole, IgA positive, and IgA negative). Data are based on the cumulative sum scaling normalized counts for each OTU.

To quantify the shared and unique taxa between the different microbiome fractions, Venn diagrams were generated at both the operational taxonomic unit (OTU) and genus-levels (Figure 5). Two hundred and ninety-three OTUs were shared among all microbiome fractions, and the whole microbiome community had 26 unique OTUs, the IgA positive had 38, and the IgA negative had 28, as shown in Figure 5a. Without sorting the IgA-positive and IgA-negative fractions, 83 OTUs would not have been identified. At the genus level, there was only one unique taxon in the whole microbiome community, while the IgA-positive community had 24 unique taxa and the IgA-negative community had 15 (Figure 5b). Although 73 genera were shared among all microbiome fractions, 51 genera were found between the IgA-positive and IgA-negative fractions and not in the whole microbiome. These 51 genera found in the IgA fractions accounted for 40.5% of the total identified genera in the study, while the IgA fractions discovered 19.9% of the total OTUs. The majority of OTUs found in the IgA fractions that were absent in the whole microbiome samples were represented by fewer than 50 reads, as seen in Supplementary Materials Table S8. These taxa were often present in at least half of the experimental groups, and many OTUs were from the Proteobacteria phylum (Supplementary Materials Figure S6). These lowly abundant bacteria may not have been detected in the whole microbiome samples due to the fact of limitations in sequencing depth and the inherent nature of random sampling during library generation. Venn diagrams for each experimental group showed a similar pattern, with 60–68 genera shared among all microbiome fractions and 5–13 unique genera in IgA-positive and IgA-negative fractions (Supplementary Materials Figure S7). Previous research shows that rare bacteria can account for up to 20% of the diversity within the bacterial microbiome, and often many remain undetected [33,34]. It is necessary to investigate the impact that vaccination with rLA has on the diversity of lowly abundant taxa. 

### 3.5. Random Forest Predictions of Important Taxa 

**Figure 6:**
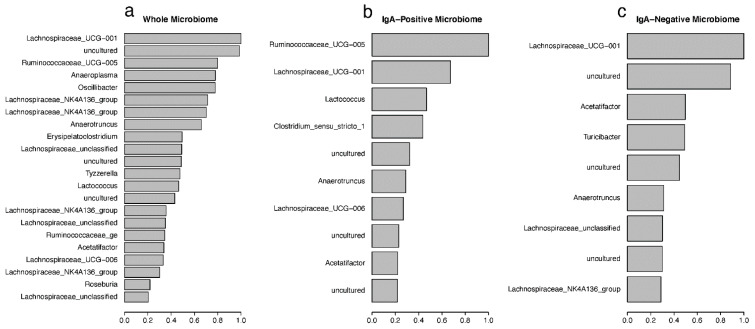
The mean decreasing Gini (MDG) coefficients plot showing the important OTUs. The MDG value represents the importance of the OTUs for (**a**) whole microbiome, (**b**) IgA-positive and (**c**) IgA-negative microbiome fractions. OTUs are labeled by assigned genera, from the random forest classification for each microbiome fraction. Cutoff was set to greater than 0.2.

The random forest (RF) [30] machine learning approach was used to classify samples into the designated treatment groups (Table 1), and important taxonomic drivers of the differences between these treatment groups were identified. Figure 6 shows the mean decreasing Gini (MDG) coefficients for the most important drivers in each microbiome fraction, calculated from OTU counts and identified by their genus classification. These MDG coefficients were calculated based on the original RF model, which uses the treatment-group as the classifier, and the OTUs with an MDG greater than 0.2 were plotted. *Lachnospiraceae_UCG-001* was identified as the most important driver for classification within the whole microbiome samples, with uncultured bacteria and *Ruminococcaceae_UCG-005* also in the top three (Figure 6a). Supplementary Materials Table S5 displays the full taxonomic description for each OTU from the whole microbiome. The top 77 OTUs all belonged to the Firmicute phylum, except for OTU0073, which is an *Anaeroplasma* in the Tenericutes phylum. The majority of these Firmicutes were classified into either the *Ruminococcaceae* or *Lachnospiraceae* family, both of which belong to the Clostridia family. Similar pattens were seen in both the IgA-positive and IgA-negative fractions (Figure 6b,c and Supplementary Materials Tables S6 and S7, respectively). Like the whole microbiome samples, Firmicutes made up the vast majority of the important taxa. OTU0023 and OTU00109 were the most important variables in both the whole microbiome and IgA-negative models, with OTU0023 (of the *Lachnospiraceae_UCG-001* genus) being the second most important variable in the IgA-positive microbiome. 

The overall out-of-bag (OOB) misclassification error rates for whole microbiome, IgA positive, and IgA negative were 28.09%, 51.04%, and 42.56%, respectively (Supplementary Materials Table S2). The highest misclassification error rates were consistently found in the NCK1895_SC diet classification (44.26% in whole, 70.69% in IgA positive, and 52.54% in IgA negative), with misclassification often occurring in the other SC groups. Treatment-group error rates for the whole microbiome ranged from 11.11% to 44.26%, with NCK1895_RB having the lowest error rate and NCK1895_SC having the highest. Within NCK1895_SC classification for whole microbiome, 34 samples were identified correctly, 7 were misclassified as Buffer_SC, 15 were misclassified as GAD19_SC, and 5 samples were misclassified into the RB groups (4 as Buffer_RB and 1 as NCK1895_RB, Supplementary Materials Table S2). The error rates in the IgA-positive RF model were high for all treatment-groups, ranging from 42.37% to 70.69%. Because of these high error rates, RF models were also generated using vaccine type (i.e., buffer, NCK1895, or GAD19; Supplementary Materials Table S3) or diet type (i.e., SC or RB; Supplementary Materials Table S4). For these RF models, the whole microbiome, again, had the lowest misclassification error rates, with the vaccine classification model showing a 16.6% error rate, and the diet classification model having a 14.6% error rate. The IgA-positive models had 41.8% and 38.1% error rates for the vaccine and diet models. The IgA-negative models showed 24.1% and 25.9% error rates also for the vaccine and diet models. These results again indicate that some of the treatments overlapped in the composition of the microbiome, and the error rates improved when the impact of the prebiotic or vaccination were used separately in the classification.

### 3.6. Lactobacilli Abundance and Importance over Time

Since four out of our six animal groups received a strain of *Lactobacillus acidophilus* (either NCK1985 or GAD19), we investigated the importance that the *Lactobacillus* genus had on RF classification and relative abundance throughout the study. Results from the RF show that *Lactobacillus* was the 15th most impactful taxon in the IgA-positive model, 64th in the whole microbiome, and 112th in the IgA negative (Supplementary Materials Tables S5–S7). The *Lactobacillus* observed in both the whole microbiome and IgA-positive tables were represented by OTU0017, while the *Lactobacillus* in the IgA-negative table was OTU0004. Due to the limitations of 16S-sequencing, we were unable to identify the species of the *Lactobacillus* OTUs that were observed, even with the use of comprehensive BLAST searches in the NCBI’s databases. Importantly, timepoint and sequencing pool (to identify any batch bias) were both included as features in the RF, neither of which were highly important for classifying the microbiome samples into treatment groups.

**Figure 7:**
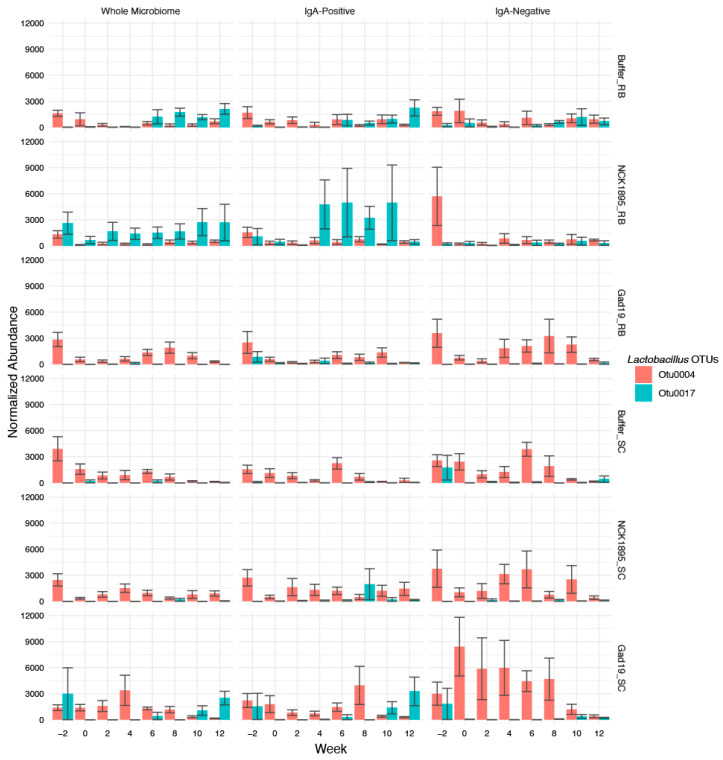
The normalized abundances of the most abundant OTUs in the *Lactobacillus* genus are shown for the whole microbiome (first column), IgA-positive microbiome (middle column), and IgA-negative microbiome (last column). The values are based on the normalized abundances calculated using cumulative sum scaling, as described in Section 2. Bars represent the mean count for each group at each timepoint, with error bars showing the standard error.

We also investigated changes in the normalized relative abundances of *Lactobacillus* species between the different microbiome fractions overtime (Figure 7). The greatest changes between microbiome fractions was observed in the GAD19_SC group. The abundance of *Lactobacillus* within this group was greatest in the IgA-negative fraction until week 10, where the relative levels of *Lactobacillus* were higher in the IgA-positive fraction. This pattern was not observed in the GAD19_RB group, where *Lactobacillus* levels were consistently higher in either the IgA-positive group or whole microbiome. Alternatively, the NCK1895_RB group had high levels of *Lactobacillus* abundance in the IgA-bound and whole microbiome fractions. OTU0017 also appeared to be more prevalent in the IgA-positive and whole microbiome communities compared to the IgA-negative fraction, especially within RB groups, while OTU0004 accounted for the majority of the detected *Lactobacillus* in the IgA-negative samples.

These data indicate that *Lactobacillus* was most impactful in the RF classification of the experimental groups for the IgA-positive samples, which could be attributed to the high abundance of OTU0017 in the IgA-positive fraction of the NCK1895_RB samples.

### 3.7. Correlation between Antigen-Specific IgA and Microbial Taxa

**Figure 8:**
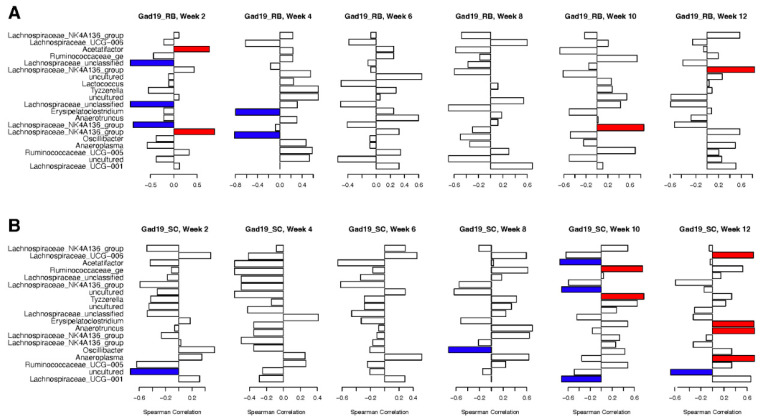
Spearman’s correlation plots between the 20 most impactful taxa from the whole microbiome and MPER-specific IgA production in the fecal samples. Positive correlations are represented in red and negative correlations in blue. No correction for multiple testing was applied, and significance was set to <0.1.

Data from MPER-specific fecal ELISAs (Figure 2) were used in Spearman’s correlation to highlight associations between specific taxa and increased MPER-specific antibodies. To remove negative observations, only GAD19 vaccinated groups were included in correlation to the 20 most important taxa from the whole microbiome at the OTU level, found using RF. Figure 8 shows significant positive (red) and negative (blue) correlations between MPER-specific IgA and the presence of specific OTUs, without correction for multiple testing. Antigen-specific IgA associated with the RB group displayed negative correlation with several *Lachnospiraceae* taxa in weeks 2 and 4, but no negative correlations with any other taxa were found after week 4 (Figure 8A). Positive correlations associated with the RB group included *Acetatifactor* and *Lachnospiraceae_NK4A136_group* at week 2, *Anaerotruncus* at week 10, and *Lachnospiraceae_NK4A136_group* again at week 12.

Conversely, antigen-specific IgA within the SC diet group displayed both positive and negative correlations with several different taxa during weeks 10 and 12 (Figure 8B). Significant negative correlations included *Acetifactor, Lachnospiraceae_UCG-001*, and an uncultured bacterium in the Lachnospiraceae family. Only *Lachnospiraceae_UCG-001* continued with a negative correlation during week 12. Week 10 had two taxa that were positively correlated with antigen-specific IgA in the GAD19_SC group: *Ruminococcaceae_ge* and *Lactococcus*. However, during week 12, there were four taxa with positive correlations including *Lachnospiraceae_UCG-006, Erysipelatoclostridium, Anaeroplasma,* and *Anaerotruncus*.

These results follow patterns with MPER-specific antibodies between the two diet groups, with more correlations found at earlier timepoints for the RB groups and most correlations for the SC groups found in weeks 10 and 12. 

## 4. Discussion

The rLA vaccine platform offers several logistical and immunological advantages over parenterally delivered vaccines. Immunological results from this study highlight the ability of orally delivered vaccines to induce both mucosal and systemic humoral immune responses. The presence of MPER-specific IgG indicates that a systemic immune response was mounted, while MPER-specific IgA detected in both fecal and vaginal samples highlight the ability of the rLA vaccine (GAD19) to stimulate the gut-associated lymphoid tissues (GALT), leading to the migration of effector cells to distant mucosal tissues. This property of oral vaccination allows for both local control of mucosally transmitted pathogens as well as surveillance for systemic spread [1,35,36]. 

The present study shows the possibility of improving the humoral immune response through the use of the prebiotic rice bran. Previous studies showed that a 10% rice bran diet increased the abundance of *Lactobacillus* populations and increased levels of secretory IgA in animals and humans [37,38,39]. The use of prebiotics could be a solution to the known variation in vaccine efficacy between high-income and low- or middle-income countries [40], where Rotavirus vaccines, for example, are 30–50% less efficacious, possibly due to the poor sanitation, malnutrition of mothers and their children, and chronic intestinal inflammation [34,40,41,42,43]. Rice bran is especially attractive, as it is a globally accessible and abundant grain byproduct [37]. However, because this study did not recapitulate a state of malnutrition in mice, the full potential of rice bran as a prebiotic could not be assessed. Future studies using animal models of malnutrition and/or dysbiosis would better simulate conditions of people in developing countries. The accelerated antibody induction in the rice bran supplemented animals in this study justifies continued investigation of prebiotics to enhance the immune response to rLA in individuals with compromised mucosal immunity.

We used IgA-seq to investigate potential disturbance of the microbiome fraction that is recognized by the mucosal immune system. We showed that the IgA-positive and IgA-negative fractions accounted for 40% of the diversity within identified genera and 20% of OTUs. This is in concordance with results using a similar technique of bacterial sorting reported by D’Auria et al., where 20% of the total diversity was attributed to rare bacteria [33]. This occurrence is hypothesized to be due to the use of traditional sequencing methods, which may not recover these rare taxa, or data processing that filters out rare taxa. In agreement with the literature [33], we showed that these lowly abundant bacteria have a greater likelihood of being sequenced as a result of IgA sorting. Additionally, many of the taxa absent from the whole microbiome group but present in the IgA fractions were members of the Proteobacteria phylum, which has also been observed by others [33,44]. Due to the pathogenic nature of many members of the Proteobacteria phylum, including *Escherichia*, *Shigella*, *Salmonella*, *Brucella*, and *Helicobacter*, a high abundance of these species is often not found in healthy individuals [45]. It has also been proposed that rare species provide a pool of genetic resources that are utilized when necessary [46,47]. 

Significant changes in the predicted alpha diversity were not found throughout the study, but the beta diversity was altered, with diet as a primary driver and the *L. acidophilus* platform contributing to intermediate shifts. This pattern of a change in diet leading to alterations in beta diversity but not alpha diversity has been documented previously [48], but to date, the diversity of IgA-positive and IgA-negative fractions has not been evaluated. We showed that the IgA-positive communities had a closer resemblance to trends observed in the beta diversity of the whole microbiome samples compared to the IgA-negative communities, especially regarding longitudinal shifts from the initial change in diet. Previously, we showed the impact of rLA on the whole microbiome and found IL-1β secretion from rLA to have the greatest impact on the variation in the beta diversity [20]. However, the results presented here demonstrate that rice bran led to the greatest changes in beta diversity. These results also indicate that separation between the negative control groups (Buffer) and the *L. acidophilus* vaccinated groups (GAD19 and NCK1895) was likely due to the probiotic itself and not a result of the rLA vaccine construct alone. The impacts of probiotics on the resident microbiome have been documented; however, alterations to the community structure were short-lived, at least in the fecal microbiome [49,50,51,52,53]. 

The influence of *Lactobacillus* and *Lachnospiraceae* was observed through the random forests classification of the experimental groups. *Lactobacillus*, specifically OTU0004, was classified as the 15th most impactful taxon in the IgA-positive fractions based on the Gini coefficient. *Lactobacillus* (represented by OTU0017) was 64th in the whole microbiome and 112th in the IgA-negative fractions. These results suggest that the IgA-positive samples relied more on the presence of *Lactobacillus* to identify the experimental group to which they belonged compared to the IgA-negative and whole microbiome samples. OTU0017 had a higher relative abundance in the NCK1895_RB and GAD19_SC groups for both the whole and IgA-positive microbiomes, while OTU0004 was often the most abundant *Lactobacillus* OTU in the other experimental groups. The Gini coefficient highlighted several *Lachnospiraceae* taxa as being highly important for classification, especially the genus *Lachnospiraceae_NK4A136_group*. Correlation analysis also showed the importance of *Lachnospiraceae* abundance with antigen-specific IgA production. The function of many members of the *Lachnospiraceae* family and the *Erysipelatoclostridium* genus have an associated role in obesity, diabetes, and intestinal inflammation, although findings are often strain dependent [54,55]. Notably, *Lachnospiraceae* are also among the main producers of short-chain fatty acids (SCFAs) in the gut. *Lachnospiraceae_NK4A136_group* and *Ruminococcaceae* are both known butyrate producers and were correlated with enhanced gut barrier function and lower long-term weight gain when a high fiber diet was consumed [56,57]. Others have shown how a change in diet, including salt, walnuts, and processed foods, can alter the abundance of members of the *Clostridia* class, specifically *Lachnospiraceae* and *Ruminococcaceae* [58,59,60]. Our results showed an overall negative correlation between *Lachnospiraceae* and antigen-specific IgA production for mice on the RB diet early in the study and both positive and negative correlations between several taxa in the *Lachnospiraceae* family in the SC diet at the end of the study. Together, these results suggest *Lachnospiraceae* may play a dual role, based on the strain and environment within the gut, in both instigating and resolving inflammation.

## 5. Conclusions

In this study, IgA-seq was used to evaluate if the combined effects from rLA vaccination and the activated mucosal immune response impacted the IgA-bound bacterial population of the microbiome. We also evaluated the impact that prebiotic rice bran has on the immune response against antigens orally delivered utilizing rLA. Neither *L. acidophilus* itself (NCK1895) nor the rLA vaccine (GAD19) resulted in long-term alterations in the intestinal microbial community diversity. Using rice bran as a prebiotic resulted in quicker induction of mucosal and systemic humoral responses. It remains to be determined whether the observed more rapid immune response was due to the fact of shifts in the microbiome or to a direct influence of the rice bran on the performance of the rLA platform.

## Figures and Tables

**Table 1:** Experimental groups with assigned vaccine treatment and diet. Eight mice were assigned to each experimental group, except for Buffer_SC, where seven mice were used, for a total of forty-seven mice. All mice received the same number of treatment doses and were repeatedly, biweekly measured over 8 time periods, starting at week 2, for immune response and to obtain fecal samples to study the microbiome. This resulted in a total number of 376 samples per type of immune response (i.e., fecal IgA, vaginal IgA, or serum IgG), for a grand total of 1128 samples, and per IgA-coating type (i.e., IgA positive, IgA negative, or whole microbiome), also for a grand total of 1128 samples.

Group	N	Treatment	Diet
Buffer_SC	7	Carrier buffer only	Standard chow
NCK1895_SC	8	NCK1895	Standard chow
GAD19_SC	8	GAD19	Standard chow
Buffer_RB	8	Carrier buffer only	10% Rice bran
NCK1895_RB	8	NCK1895	10% Rice bran
GAD19_RB	8	GAD19	10% Rice bran

## Data Availability

Raw data are available in the National Center for Biotechnology Information’s (NCBI) Sequence Read Archive (SRA) under BioProject PRJNA542488.

## Supplementary Materials

The following supporting information can be downloaded at https://www.mdpi.com/article/10.3390/vaccines10091465/s1, Table S1. Adjusted P-values for the pairwise comparison of ELISA endpoint titers between experimental groups for each timepoint in the study. The Kruskal-Wallis test of analysis of variance was followed by Dunn’s multiple comparison post-hoc test since data was not normally distributed. The Benjamini-Hochberg method was used to adjust p-values for multiple testing; Table S2. Confusion matrix for RF model based on classification of experimental groups. The confusion matrices are shown for each microbiome fraction (whole, IgA-positive, and IgA-negative, respectively); Table S3. Confusion matrix for RF model based on classification of treatment (buffer, NCK1895, or GAD19). The confusion matrices are shown for each microbiome fraction (whole, IgA-positive, and IgA-negative, respectively); Table S4. Confusion matrix for RF model based on classification of diet (rice bran or standard chow). The confusion matrices are shown for each microbiome fraction (whole, IgA-positive, and IgA-negative, respectively); Table S5. Gini coefficients of importance for the whole microbiome features from the RF model. Extension of Gini coefficients shown in Figure 5, with OTU listed in the first column followed by taxonomic classification. The last column shows the mean Gini coefficient of importance for that feature; Table S6. Gini coefficients of importance for the IgA-positive microbiome features from the RF model; Table S7. Gini coefficients of importance for the IgA-negative microbiome features from the RF model; Table S8. Sum of library reads for each OTU. Microbiome fractions are shown in each column: whole = Whole microbiome, positive = IgA-positive, negative = IgA-negative. Figure S1. Gating of fecal samples to obtain IgA-positive and IgA-negative fractions. First, cells were gated based on SSC and FSC, the FSC-H and FSC-A to gate for single cells. IgA-negative and IgA-positive populations were selected based on intensity of FITC fluorescence and collected into sterile tubes. FITC and PE graphs were analyzed to observe spill over into the PE channel. Panel A shows unstained cells, and panel B shows cells stained with anti-mouse IgA with a FITC tag from the randomized sample 347; Figure S2. Rarefaction curves for (a) whole microbiome, (b) IgA-positive, and (c) IgA-negative fractions. Samples are each represented by a colored line, with OTU counts per sample on the *x*-axis and number of reads per sample on the *y*-axis. Horizontal lines indicate a low chance for the discovery of more OTUs based on the number of reads; Figure S3. Iteration of out-of-bag (OOB) error rates for each microbiome fraction tuning model with (a) whole microbiome, (b) IgA-positive, and (c) IgA-negative fractions. The optimal number of features for each model (separated by microbiome fraction) was selected by iterating over 100 trees, with the median error rates displayed on the *x*-axis and number of features on the *y*-axis; Figure S4. Alpha diversity represented by the predicted values of observed richness. Figures for the whole microbiome, IgA-positive, and IgA-negative microbiome fractions represented by the 95% credibility intervals. A linear mixed effects model was used to determine predicted values; Figure S5. Temporal changes in beta-diversity shown by NMDS ordination. Each experimental group is shown in sections a-f, with data separated into plots for IgA-negative, IgA-positive, and whole microbiome communities, respectively, as indicated by title above each plot. Each 95% confidence ellipsoid represents the samples taken at that timepoint, with number at the end of the label corresponding with the week of the sample collection; Figure S6. Normalized abundances of OTUs found only in IgA-positive or IgA-negative fractions. Each OTU is labeled with the phylum and genus it belongs to, and the relative abundance for each experimental group. Normalized abundances were calculated by Cumulative Sum Scaling as described in the Methods; Figure S7. Unique and shared genera between microbiome fractions for each experimental group. Each Venn diagram represents genera found in each mouse group used in this study.
