# Supplementary material for: Oral Vaccination Using a Probiotic Vaccine Platform Combined with Prebiotics Impacts Immune Response and the Microbiome
Source: Vaccines (Basel). 2022 Sep 4;10(9):1465. doi: 10.3390/vaccines10091465 (PMC9504555; doi:10.3390/vaccines10091465)
Supplement: Supplementary file 1 [file vaccines-10-01465-s001.zip › IgA-Seq_Supplementary_Information_vaccines_Final.pdf]

Oral vaccination using a probiotic vaccine platform combined with prebiotics impacts immune response and the microbiome

Bridget E. Fox[1,*], Allison Vilander[1], Darby Gilfillan[1], Gregg A. Dean[1,*], and Zaid Abdo[1,*]

[1]Department of Microbiology, Immunology, and Pathology, Colorado State University, Fort Collins, Colorado 80523, USA

[*]Corresponding authors

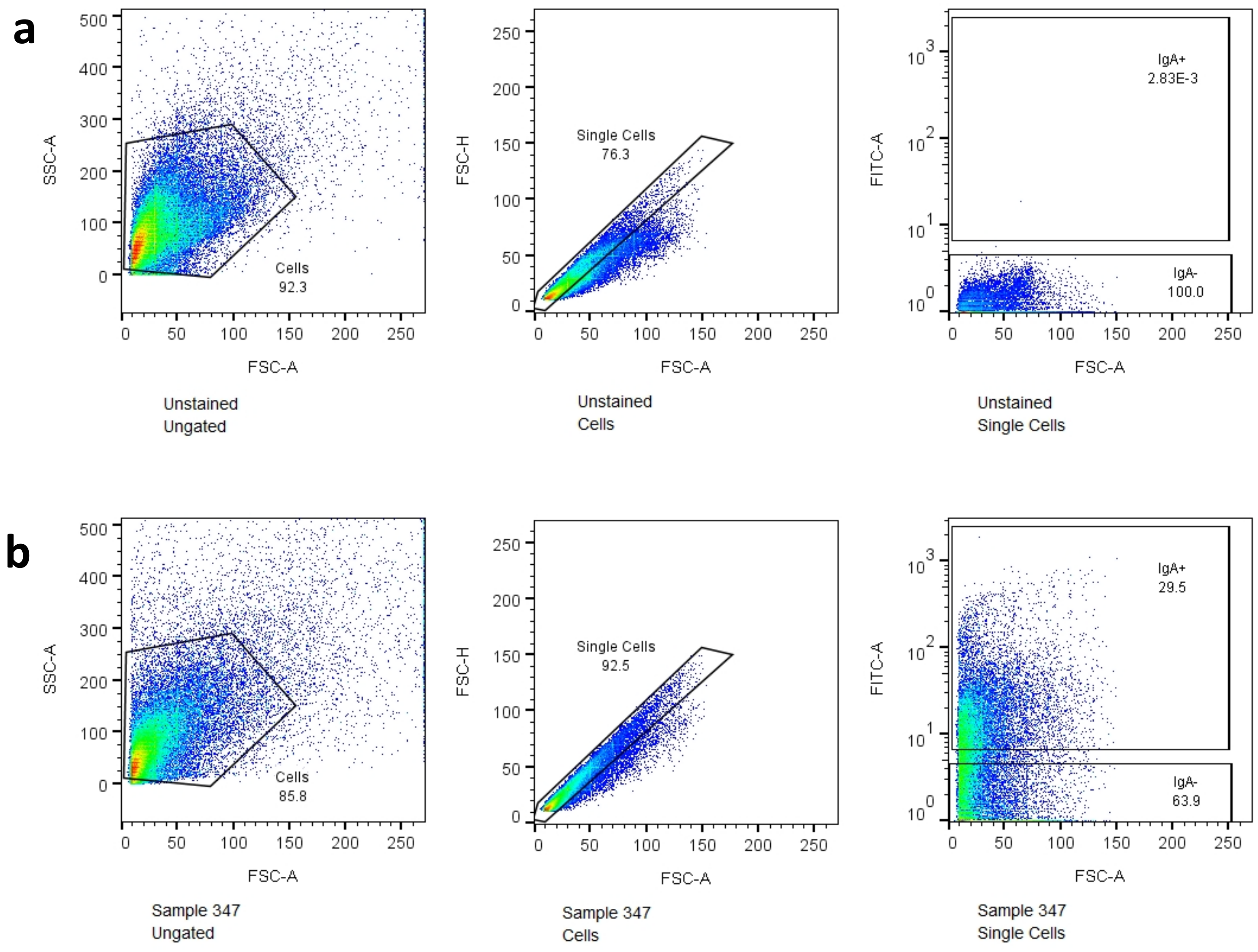

Supplementary Figure S1. Gating of fecal samples to obtain IgA-positive and IgA-negative fractions. First, cells were gated based on SSC and FSC, the FSC-H and FSC-A to gate for single cells. IgA-negative and IgA-positive populations were selected based on intensity of FITC fluorescence and collected into sterile tubes. FITC and PE graphs were analyzed to observe spill over into the PE channel. Panel A shows unstained cells, and panel B shows cells stained with anti-mouse IgA with a FITC tag from the randomized sample 347.

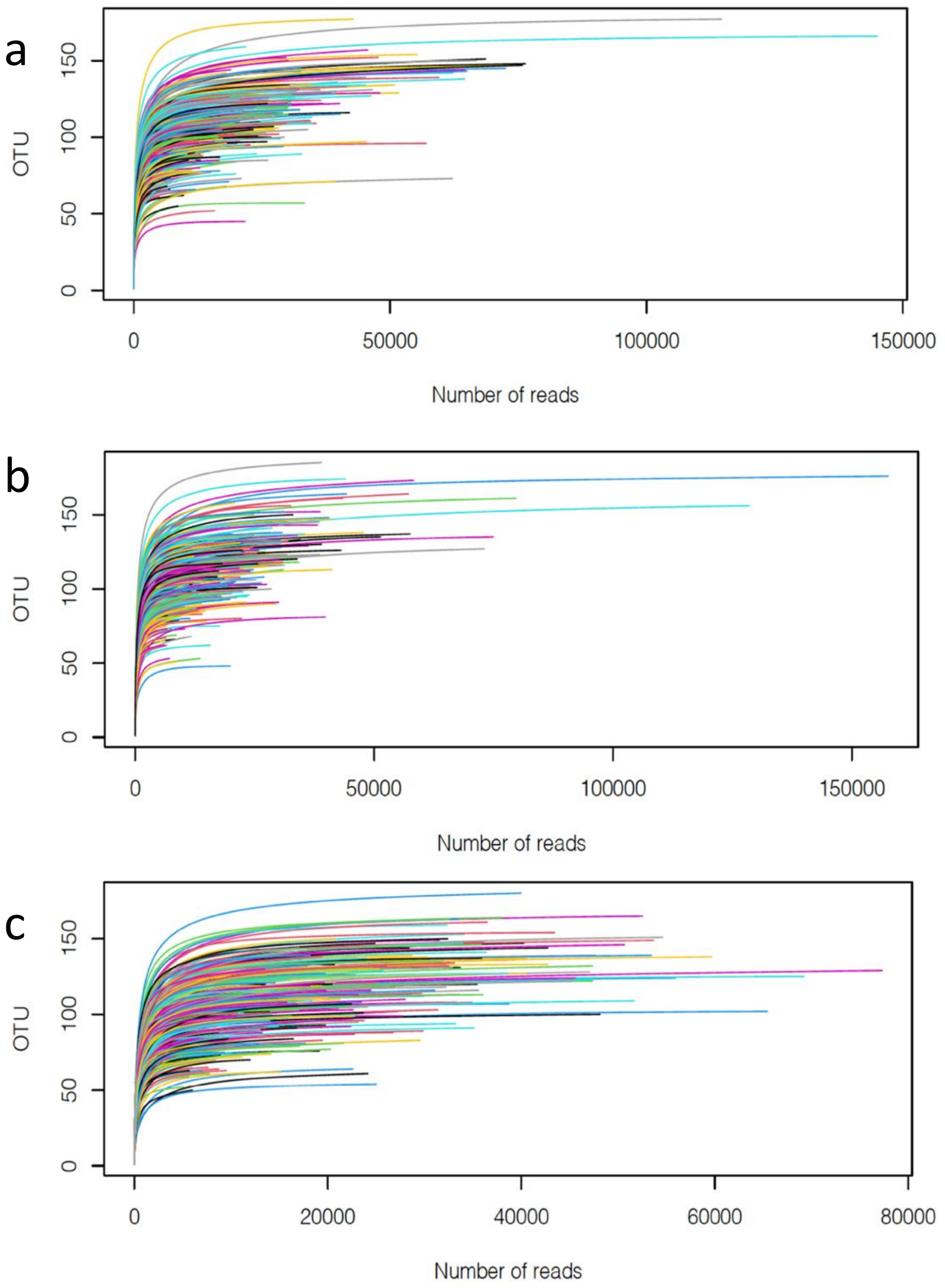

Supplementary Figure S2. Rarefaction curves for (a) whole microbiome, (b) IgA-positive, and (c) IgA-negative fractions. Samples are each represented by a colored line, with OTU counts per sample on the X-axis and number of reads per sample on the y-axis. Horizontal lines indicate a low chance for the discovery of more OTUs based on the number of reads.

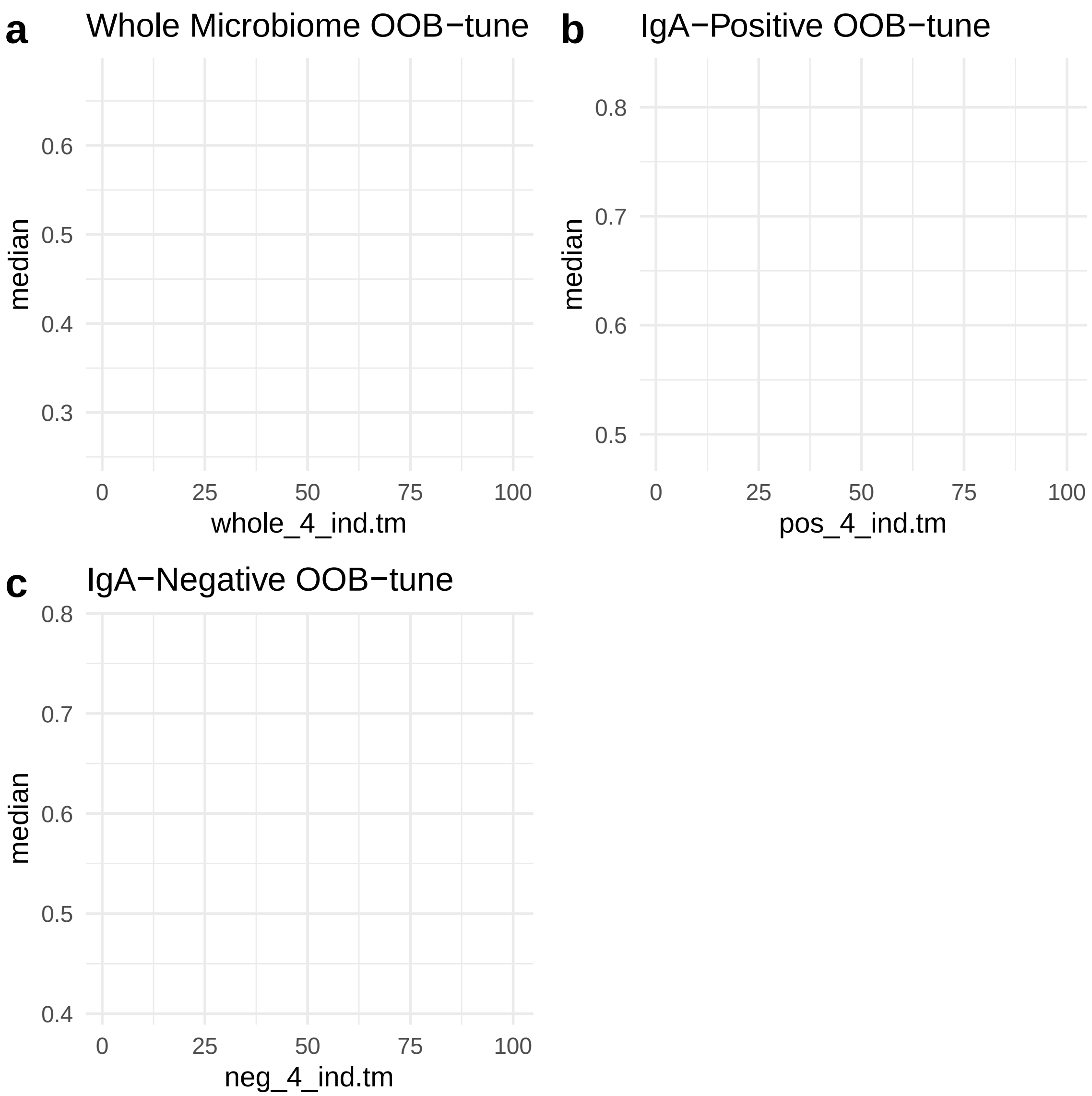

Supplementary Figure S3. Iteration of out-of-bag (OOB) error rates for each microbiome fraction tuning model with (a) whole microbiome, (b) IgA-positive, and (c) IgA-negative fractions. The optimal number of features for each model (separated by microbiome fraction) was selected by iterating over 100 trees, with the median error rates displayed on the x-axis and number of features on the y-axis.

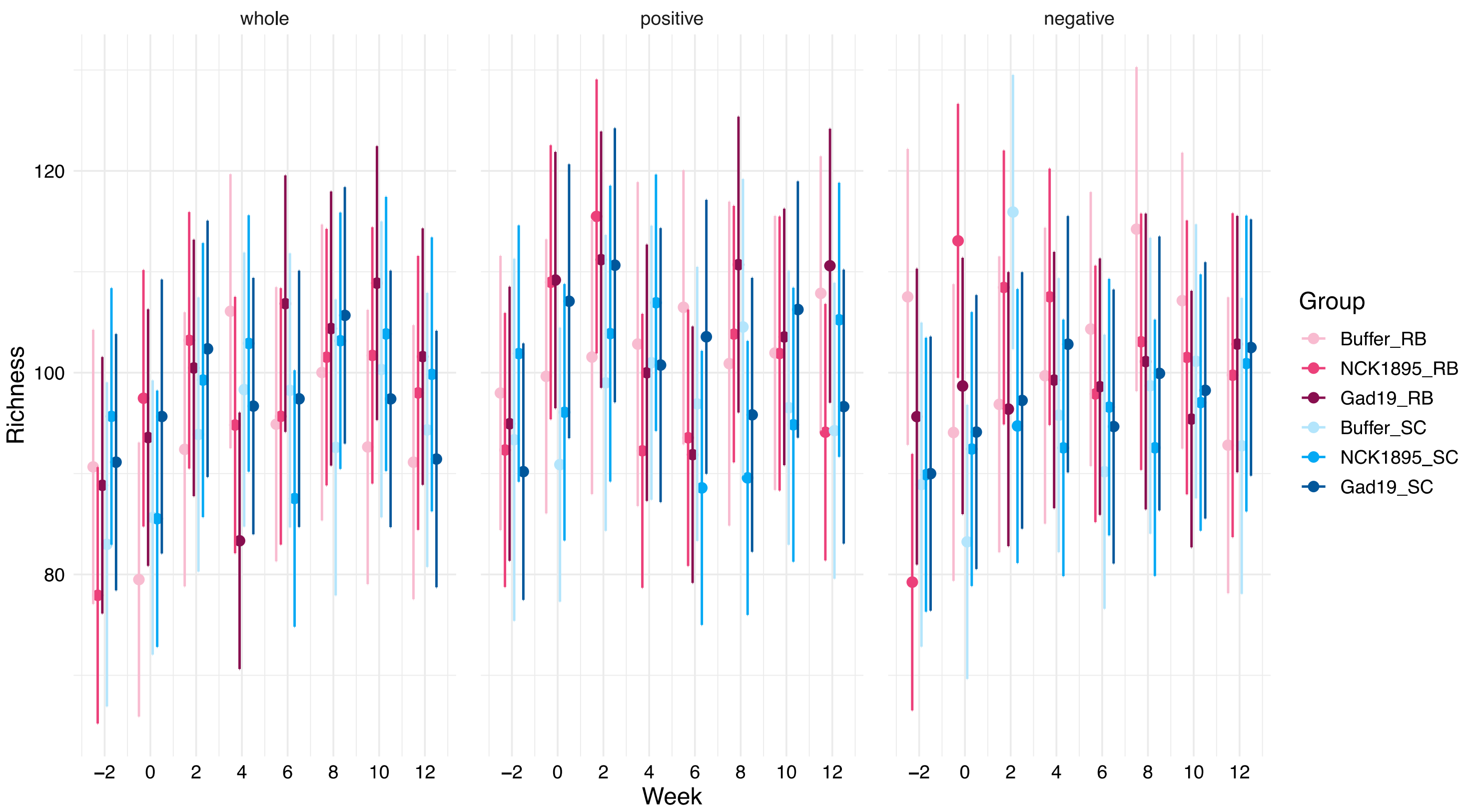

Supplementary Figure S4. Alpha diversity represented by the predicted values of observed richness. Figures for the whole microbiome, IgA-positive, and IgA-negative microbiome fractions represented by the 95% credibility intervals. A linear mixed effects model was used to determine predicted values.

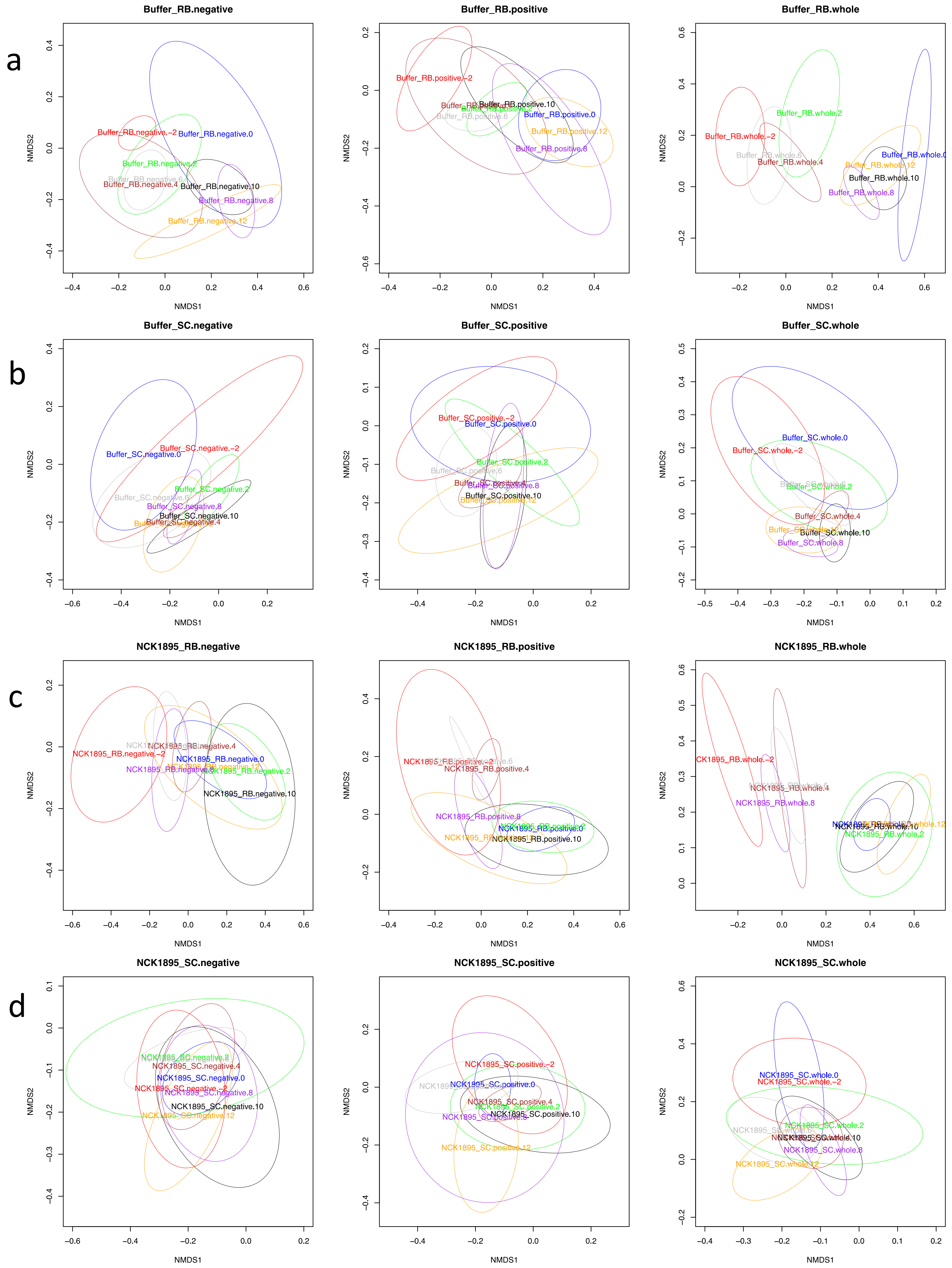
a
Buffer_RB.negative
Buffer_RB.positive
Buffer_RB.whole
b
Buffer_SC.negative
Buffer_SC.positive
Buffer_SC.whole
c
NCK1895_RB.negative
NCK1895_RB.positive
NCK1895_RB.whole
d
NCK1895_SC.negative
NCK1895_SC.positive
NCK1895_SC.whole
NMDS1
NMDS2
Buffer_RB.negative.−2
Buffer_RB.negative.0
Buffer_RB.negative.2
Buffer_RB.negative.4
Buffer_RB.negative.10
Buffer_RB.negative.8
Buffer_RB.negative.12
Buffer_RB.positive.−2
Buffer_RB.positive.10
Buffer_RB.positive.0
Buffer_RB.positive.6
Buffer_RB.positive.12
Buffer_RB.positive.8
Buffer_RB.whole.2
Buffer_RB.whole.−2
Buffer_RB.whole.6
Buffer_RB.whole.4
Buffer_RB.whole.12
Buffer_RB.whole.10
Buffer_RB.whole.8
Buffer_SC.negative.−2
Buffer_SC.negative.0
Buffer_SC.negative.6
Buffer_SC.negative.2
Buffer_SC.negative.8
Buffer_SC.negative.10
Buffer_SC.negative.4
Buffer_SC.positive.−2
Buffer_SC.positive.0
Buffer_SC.positive.2
Buffer_SC.positive.6
Buffer_SC.positive.4
Buffer_SC.positive.8
Buffer_SC.positive.10
Buffer_SC.positive.12
Buffer_SC.whole.0
Buffer_SC.whole.−2
Buffer_SC.whole.2
Buffer_SC.whole.4
Buffer_SC.whole.10
Buffer_SC.whole.8
NCK1895_RB.negative.4
NCK1895_RB.negative.−2
NCK1895_RB.negative.0
NCK1895_RB.negative.2
NCK1895_RB.negative.10
NCK1895_RB.positive.4
NCK1895_RB.positive.8
NCK1895_RB.positive.0
NCK1895_RB.positive.10
NCK1895_RB.whole.4
NCK1895_RB.whole.8
NCK1895_RB.whole.10
NCK1895_RB.whole.2
NCK1895_SC.negative.4
NCK1895_SC.negative.0
NCK1895_SC.negative.−2
NCK1895_SC.negative.8
NCK1895_SC.negative.10
NCK1895_SC.negative.12
NCK1895_SC.positive.−2
NCK1895_SC.positive.0
NCK1895_SC.positive.4
NCK1895_SC.positive.2
NCK1895_SC.positive.10
NCK1895_SC.positive.12
NCK1895_SC.whole.0
NCK1895_SC.whole.−2
NCK1895_SC.whole.2
NCK1895_SC.whole.10
NCK1895_SC.whole.8
NCK1895_SC.whole.12

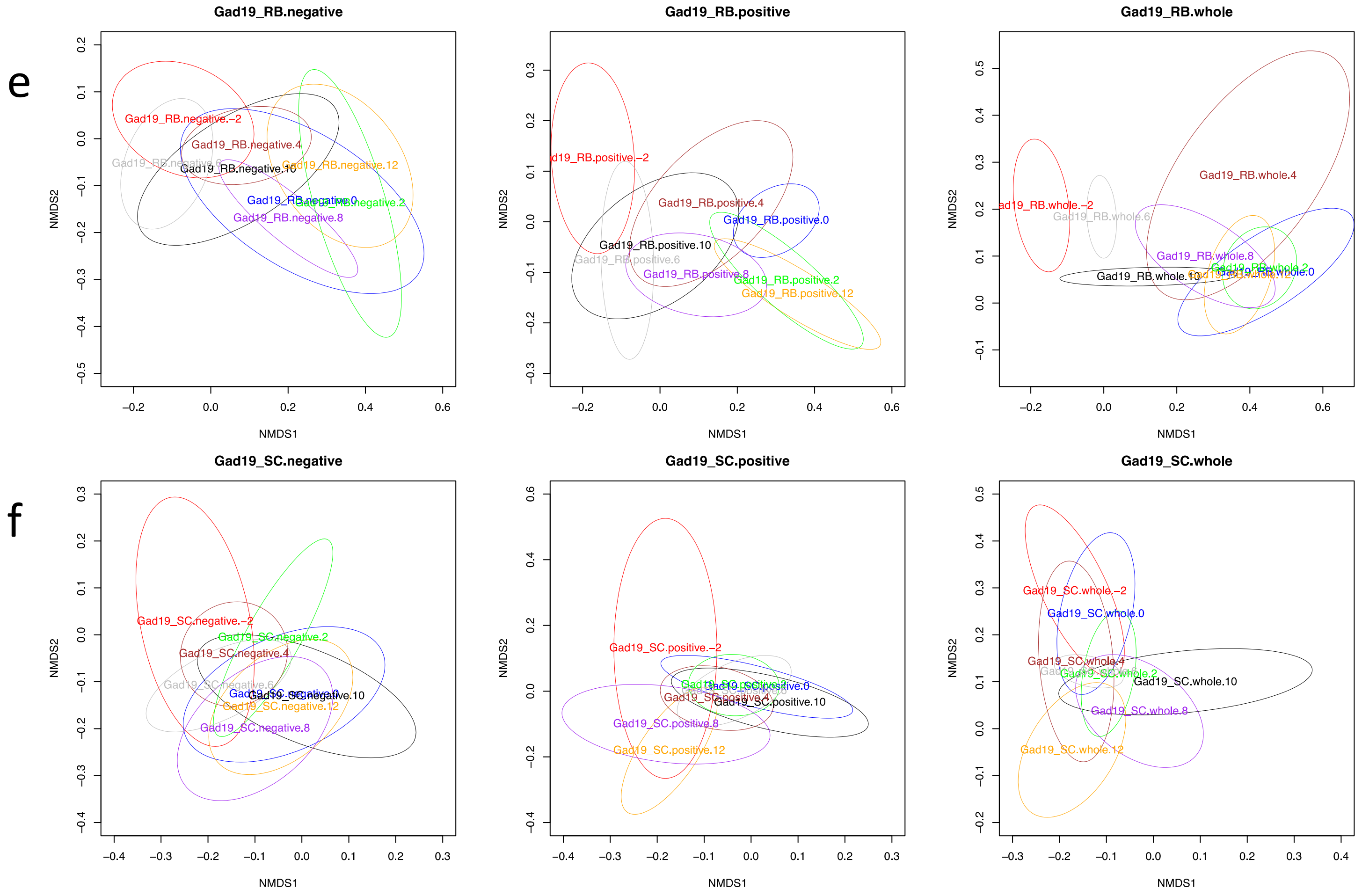

Supplementary Figure S5. Temporal changes in beta-diversity shown by NMDS ordination. Each experimental group is shown in sections a-f, with data separated into plots for IgA-negative, IgA-positive, and whole microbiome communities, respectively, as indicated by title above each plot. Each 95% confidence ellipsoid represents the samples taken at that timepoint, with number at the end of the label corresponding with the week of the sample collection.

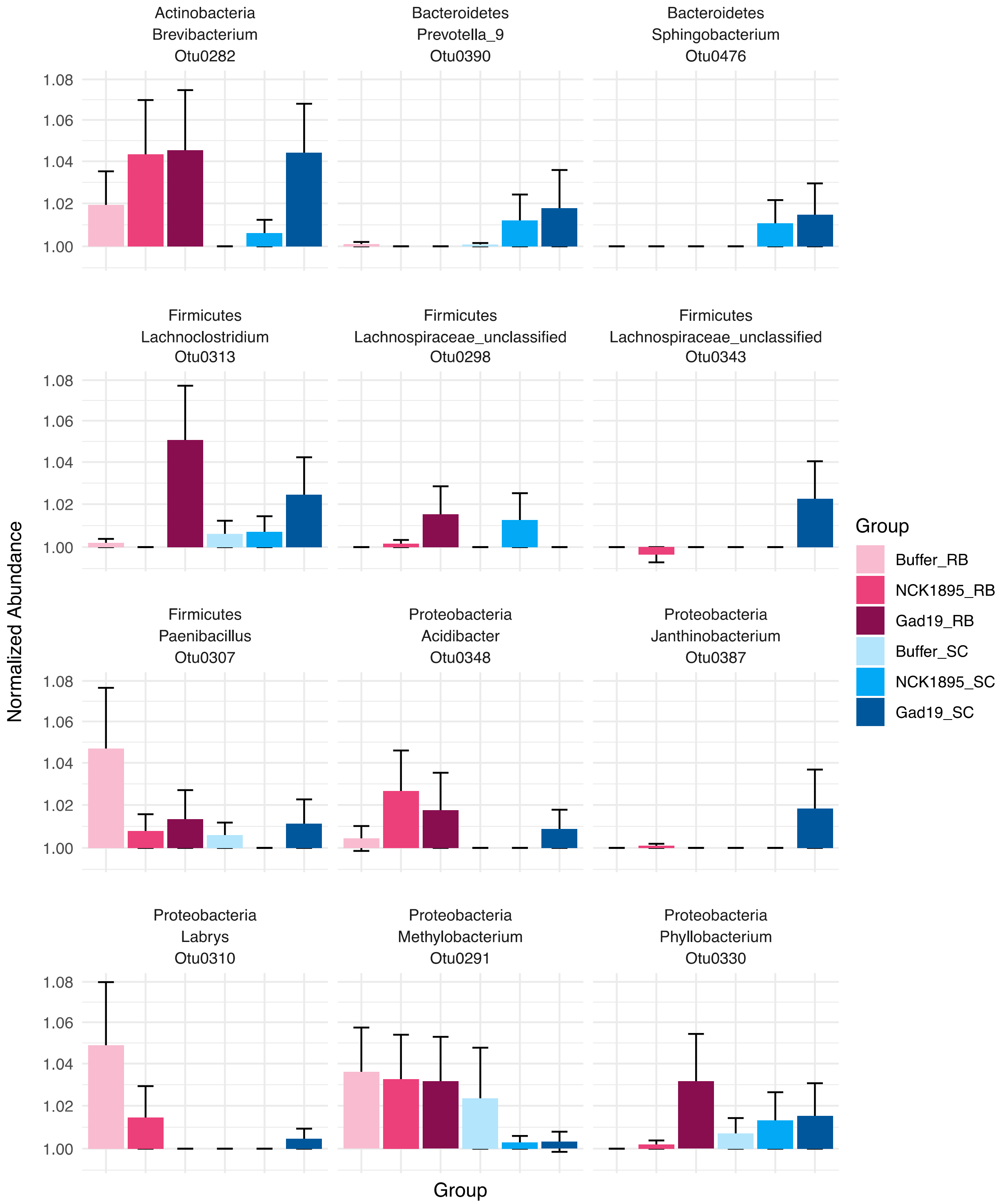

Supplementary Figure S6. Normalized abundances of OTUs found only in IgA-positive or IgA-negative fractions. Each OTU is labeled with the phylum and genus it belongs to, and the relative abundance for each experimental group. Normalized abundances were calculated by Cumulative Sum Scaling as described in the Methods.

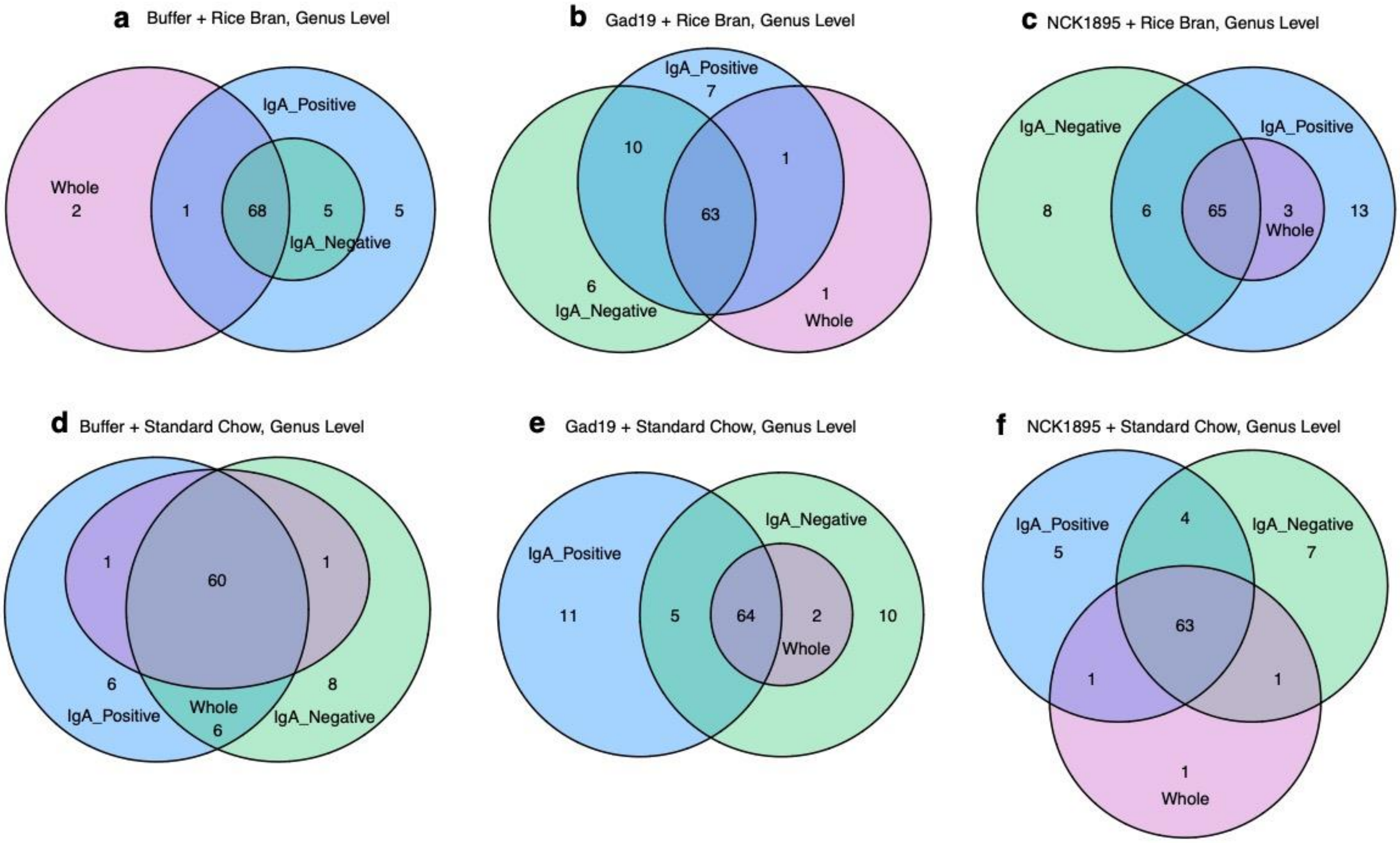

Supplementary Figure S7. Unique and shared genera between microbiome fractions for each experimental group. Each Venn diagram represents genera found in each mouse group used in this study.

Supplementary Tables

Supplementary Table S1. Adjusted P-values for the pairwise comparison of ELISA endpoint titers between experimental groups for each timepoint in the study. The Kruskal-Wallis test of analysis of variance was followed by Dunn's multiple comparison post-hoc test since data was not normally distributed. The Benjamini-Hochberg method was used to adjust p-values for multiple testing.

**Source**	**Comparison-1**	**Comparison-2**	**Week_2**	**Week_4**	**Week_6**	**Week_8**	**Week_10**	**Week_12**
Fecal	Buffer_RB	Buffer_SC	0.3001	0.3650	0.5000	0.3938	0.4316	0.3928
Fecal	Buffer_RB	Gad19_RB	0.1057	0.0017	0.0004	0.0023	0.0003	0.0003
Fecal	Buffer_SC	Gad19_RB	0.0184	0.0002	0.0005	0.0008	0.0004	0.0008
Fecal	Buffer_RB	Gad19_SC	0.3143	0.5702	0.0489	0.0015	0.0003	0.0004
Fecal	Buffer_SC	Gad19_SC	0.1600	0.4362	0.0559	0.0008	0.0007	0.0015
Fecal	Gad19_RB	Gad19_SC	0.2022	0.0020	0.0619	0.4643	0.4906	0.4440
Fecal	Buffer_RB	NCK1895_RB	0.3301	0.4015	0.5357	0.4091	0.4709	0.3347
Fecal	Buffer_SC	NCK1895_RB	0.5000	0.5000	0.5769	0.2630	0.5000	0.4506
Fecal	Gad19_RB	NCK1895_RB	0.0276	0.0003	0.0007	0.0090	0.0005	0.0016
Fecal	Gad19_SC	NCK1895_RB	0.1866	0.4986	0.0652	0.0062	0.0008	0.0029
Fecal	Buffer_RB	NCK1895_SC	0.3668	0.4461	0.6250	0.4266	0.5357	0.5000
Fecal	Buffer_SC	NCK1895_SC	0.5357	0.5357	0.6818	0.5000	0.5179	0.4286
Fecal	Gad19_RB	NCK1895_SC	0.0552	0.0006	0.0015	0.0011	0.0005	0.0006
Fecal	Gad19_SC	NCK1895_SC	0.2240	0.5817	0.0782	0.0017	0.0004	0.0005
Fecal	NCK1895_RB	NCK1895_SC	0.5769	0.5769	0.7500	0.2922	0.5755	0.3719

**Source**	**Comparison-1**	**Comparison-2**	**Week_2**	**Week_4**	**Week_6**	**Week_8**	**Week_10**	**Week_12**
Serum	Buffer_RB	Buffer_SC	0.1249	0.2790	0.5000	0.3306	0.5000	0.3851
Serum	Buffer_RB	Gad19_RB	0.1561	0.1759	0.0920	0.0286	0.0104	0.0009
Serum	Buffer_SC	Gad19_RB	0.5000	0.0419	0.1226	0.0056	0.0119	0.0029
Serum	Buffer_RB	Gad19_SC	0.2082	0.5000	0.1321	0.1656	0.0008	0.0019
Serum	Buffer_SC	Gad19_SC	0.5357	0.3069	0.2641	0.0510	0.0011	0.0057
Serum	Gad19_RB	Gad19_SC	0.5769	0.2199	0.5138	0.2351	0.2720	0.3998
Serum	Buffer_RB	NCK1895_RB	0.3122	0.3410	0.3405	0.3606	0.3778	0.3124
Serum	Buffer_SC	NCK1895_RB	0.6250	0.5357	0.3714	0.5000	0.4093	0.2012
Serum	Gad19_RB	NCK1895_RB	0.6818	0.0629	0.1622	0.0084	0.0035	0.0002
Serum	Gad19_SC	NCK1895_RB	0.7500	0.3836	0.1669	0.0595	0.0003	0.0003
Serum	Buffer_RB	NCK1895_SC	0.6245	0.4385	0.4086	0.3967	0.4465	0.4172
Serum	Buffer_SC	NCK1895_SC	0.8333	0.5769	0.4540	0.5357	0.4911	0.2928
Serum	Gad19_RB	NCK1895_SC	0.9375	0.1258	0.1854	0.0168	0.0042	0.0003
Serum	Gad19_SC	NCK1895_SC	1.0000	0.5115	0.2003	0.0714	0.0005	0.0006
Serum	NCK1895_RB	NCK1895_SC	1.0000	0.6250	0.5357	0.5769	0.5357	0.4043

**Source**	**Comparison-1**	**Comparison-2**	**Week_2**	**Week_4**	**Week_6**	**Week_8**	**Week_10**	**Week_12**

Vaginal	Buffer_RB	Buffer_SC	0.2022	0.2920	0.3445	0.5000	0.4096	0.3785
Vaginal	Buffer_RB	Gad19_RB	0.5000	0.4080	0.1719	0.2248	0.0467	0.0068
Vaginal	Buffer_SC	Gad19_RB	0.2311	0.2380	0.0826	0.2698	0.0172	0.0028
Vaginal	Buffer_RB	Gad19_SC	0.2697	0.3212	0.0992	0.0753	0.0010	0.0010
Vaginal	Buffer_SC	Gad19_SC	0.5357	0.5000	0.0674	0.1506	0.0002	0.0007
Vaginal	Gad19_RB	Gad19_SC	0.3236	0.3570	0.3881	0.3486	0.1106	0.3455
Vaginal	Buffer_RB	NCK1895_RB	0.4045	0.3569	0.3732	0.3639	0.4468	0.5000
Vaginal	Buffer_SC	NCK1895_RB	0.5769	0.5357	0.5000	0.3942	0.5000	0.4076
Vaginal	Gad19_RB	NCK1895_RB	0.5393	0.7139	0.1101	0.3245	0.0200	0.0079
Vaginal	Gad19_SC	NCK1895_RB	0.6250	0.5769	0.1348	0.1222	0.0003	0.0015
Vaginal	Buffer_RB	NCK1895_SC	0.8090	0.4015	0.3721	0.4301	0.4915	0.4416
Vaginal	Buffer_SC	NCK1895_SC	0.6818	0.1414	0.2144	0.4731	0.5357	0.3386
Vaginal	Gad19_RB	NCK1895_SC	1.0000	0.5830	0.2837	0.3651	0.0241	0.0175
Vaginal	Gad19_SC	NCK1895_SC	0.7500	0.1697	0.2329	0.1629	0.0007	0.0029
Vaginal	NCK1895_RB	NCK1895_SC	0.8333	0.2121	0.2412	0.5357	0.5769	0.4817

Supplementary Table S2. Confusion matrix for RF model based on classification of experimental groups. The confusion matrices are shown for each microbiome fraction (whole, IgA-positive, and IgA-negative, respectively).

```
Whole Microbiome:
Call:
 randomForest(x = x_4_whole, y = y_4_whole, ntree = 1000, mtry = 46,      importance = TRUE)
               Type of random forest: classification
                     Number of trees: 1000
No. of variables tried at each split: 46

        OOB estimate of  error rate: 28.09%
Confusion matrix:
```

	Buffer_RB	NCK1895_RB	Gad19_RB	Buffer_SC	NCK1895_SC	Gad19_SC	class.error
Buffer_RB	37	1	2	12	3	0	0.3272727
NCK1895_RB	1	56	2	1	2	1	0.1111111
Gad19_RB	2	4	43	0	1	12	0.3064516
Buffer_SC	4	2	0	34	8	4	0.3461538
NCK1895_SC	4	1	0	7	34	15	0.4426230
Gad19_SC	0	4	5	0	2	52	0.1746032

```
IgA−Positive Microbiome:

Call:
 randomForest(x = x_4_pos, y = y_4_pos, ntree = 1000, mtry = 98,      importance = TRUE)
               Type of random forest: classification
                     Number of trees: 1000
No. of variables tried at each split: 98

        OOB estimate of  error rate: 51.04%
Confusion matrix:
```

	Buffer_RB	NCK1895_RB	Gad19_RB	Buffer_SC	NCK1895_SC	Gad19_SC	class.error
Buffer_RB	26	2	5	7	6	6	0.5000000
NCK1895_RB	5	34	8	4	2	6	0.4237288
Gad19_RB	7	9	30	2	1	11	0.5000000
Buffer_SC	3	3	0	28	9	7	0.4400000
NCK1895_SC	7	7	2	12	17	13	0.7068966
Gad19_SC	3	6	6	5	8	30	0.4827586

```
IgA−Negative Microbiome:

Call:
 randomForest(x = x_4_neg, y = y_4_neg, ntree = 1000, mtry = 60,      importance = TRUE)
               Type of random forest: classification
                     Number of trees: 1000
No. of variables tried at each split: 60

        OOB estimate of  error rate: 42.56%
Confusion matrix:
```

	Buffer_RB	NCK1895_RB	Gad19_RB	Buffer_SC	NCK1895_SC	Gad19_SC	class.error
Buffer_RB	20	3	5	6	8	6	0.5833333
NCK1895_RB	2	32	12	7	3	2	0.4482759
Gad19_RB	2	3	39	1	4	10	0.3389831
Buffer_SC	2	3	3	31	11	2	0.4038462
NCK1895_SC	2	2	3	11	28	13	0.5254237
Gad19_SC	3	5	4	2	3	43	0.2833333

Supplementary Table S3. Confusion matrix for RF model based on classification of treatment (buffer, NCK1895, or GAD19). The confusion matrices are shown for each microbiome fraction (whole, IgA-positive, and IgA-negative, respectively).

```
Whole:
Call:
 randomForest(x = x_2_whole, y = y_2_whole, ntree = 1000, mtry = 77,      importance = TRUE)
               Type of random forest: classification
                     Number of trees: 1000
No. of variables tried at each split: 77

        OOB estimate of  error rate: 16.57%
Confusion matrix:
       Buffer Gad19 NCK1895 class.error
Buffer     96     3       8   0.1028037
Gad19       4   110      11   0.1200000
NCK1895    16    17      91   0.2661290
```

```
IgA_Positive
Call:
 randomForest(x = x_2_pos, y = y_2_pos, ntree = 1000, mtry = 80,      importance = TRUE)
               Type of random forest: classification
                     Number of trees: 1000
No. of variables tried at each split: 80

        OOB estimate of  error rate: 41.84%
Confusion matrix:
       Buffer Gad19 NCK1895 class.error
Buffer     66    23      13   0.3529412
Gad19      14    77      27   0.3474576
NCK1895    32    32      53   0.5470085
```

```
IgA_negative
Call:
 randomForest(x = x_2_neg, y = y_2_neg, ntree = 1000, mtry = 74,      importance = TRUE)
               Type of random forest: classification
                     Number of trees: 1000
No. of variables tried at each split: 74

        OOB estimate of  error rate: 38.1%
Confusion matrix:
       Buffer Gad19 NCK1895 class.error
Buffer     61    17      22   0.3900000
Gad19      11    90      18   0.2436975
NCK1895    26    34      57   0.5128205
```

Supplementary Table S4. Confusion matrix for RF model based on classification of diet (rice bran or standard chow). The confusion matrices are shown for each microbiome fraction (whole, IgA-positive, and IgA-negative, respectively).

```
Whole:
Call:
 randomForest(x = x_3_whole, y = y_3_whole, ntree = 1000, mtry = 5,      importance = TRUE)
               Type of random forest: classification
                     Number of trees: 1000
No. of variables tried at each split: 5

        OOB estimate of  error rate: 14.61%
Confusion matrix:
          Rice_bran Standard_chow  class.error
Rice_bran       144            36   0.20000000
Standard_chow    16           160   0.09090909
```

```
IgA_Positive
Call:
 randomForest(x = x_3_pos, y = y_3_pos, ntree = 1000, mtry = 66,      importance = TRUE)
               Type of random forest: classification
                     Number of trees: 1000
No. of variables tried at each split: 66

        OOB estimate of  error rate: 25.82%
Confusion matrix:
          Rice_bran Standard_chow  class.error
Rice_bran       125            46    0.2690058
Standard_chow    41           125    0.2469880
```

```
IgA_negative
Call:
 randomForest(x = x_3_neg, y = y_3_neg, ntree = 1000, mtry = 97,      importance = TRUE)
               Type of random forest: classification
                     Number of trees: 1000
No. of variables tried at each split: 97

        OOB estimate of  error rate: 24.11%
Confusion matrix:
          Rice_bran Standard_chow  class.error
Rice_bran       121            44    0.2666667
Standard_chow    37           134    0.2163743
```

Additional File 2: Supplementary Table S5 [Excel file titled Supplementary Table S5]. Gini coefficients of importance for the whole microbiome features from the RF model. Extension of Gini coefficients shown in Figure 5, with OTU listed in the first column followed by taxonomic classification. The last column shows the mean Gini coefficient of importance for that feature.

Additional File 3: Supplementary Table S6 [Excel file titled Supplementary Table S6]. Gini coefficients of importance for the IgA-positive microbiome features from the RF model.

Additional File 4: Supplementary Table S7 [Excel file titled Supplementary Table S7]. Gini coefficients of importance for the IgA-negative microbiome features from the RF model.

Additional File 5: Supplementary Table S8 [Excel file titled Supplementary Table S2]. Sum of library reads for each OTU. Microbiome fractions are shown in each column: whole = Whole microbiome, positive = IgA-positive, negative = IgA-negative.
