# Supplementary material for: Oral Vaccination Using a Probiotic Vaccine Platform Combined with Prebiotics Impacts Immune Response and the Microbiome
Source: Vaccines (Basel). 2022 Sep 4;10(9):1465. doi: 10.3390/vaccines10091465 (PMC9504555; doi:10.3390/vaccines10091465)
Supplement: Supplementary file 1 [file vaccines-10-01465-s001.zip › Table_S8_sums_OTU.pdf]

Table_S8_sums_OTU

	whole	positive	negative
Otu0001	1709017	1474756	1367776
Otu0002	436568	351124	244425
Otu0005	379173	261390	230947
Otu0003	358011	289535	316281
Otu0004	269746	230351	465654
Otu0007	254732	165773	153344
Otu0006	234806	208591	213312
Otu0010	171483	143321	128851
Otu0008	163151	153610	148050
Otu0011	146006	131964	148846
Otu0024	139525	46314	39536
Otu0014	136564	134414	109242
Otu0012	127413	138769	155581
Otu0018	126776	89287	89083
Otu0017	122515	153636	41882
Otu0013	118429	138844	131437
Otu0019	117865	82728	80817
Otu0009	115183	140495	200155
Otu0025	99403	75364	46924
Otu0016	89004	108088	136732
Otu0020	88155	97917	68900
Otu0015	87302	86571	178026
Otu0023	77385	77961	88062
Otu0021	75998	84517	96213
Otu0026	70452	64254	69448
Otu0031	65873	43348	45254
Otu0030	63078	49956	46007
Otu0029	62106	52526	44172
Otu0027	60955	54923	48308
Otu0042	52472	38041	29284
Otu0028	50185	55744	55428
Otu0040	49082	37467	30888
Otu0034	48317	44084	45411
Otu0045	47781	14177	13777
Otu0046	47494	31684	32716
Otu0032	47338	45386	49931
Otu0051	47167	26722	21451
Otu0043	46894	35730	36331
Otu0036	44325	42627	42696
Otu0033	43391	40447	56426
Otu0050	39438	30469	25427
Otu0052	38868	28923	24602
Otu0047	37748	38285	33973
Otu0037	37733	42679	48751
Otu0041	34705	42370	42081

Otu0048	33766	34990	40356
Otu0038	32027	41122	52082
Otu0073	29948	9695	7873
Otu0039	27708	37659	58844
Otu0053	27594	27945	32910
Otu0054	26290	28344	25743
Otu0070	25971	19508	5428
Otu0056	24740	24378	24734
Otu0059	24701	23582	20396
Otu0063	24420	18090	16889
Otu0068	24315	16860	14909
Otu0077	23967	10723	7094
Otu0064	23781	11867	25887
Otu0071	23740	16169	10312
Otu0057	23663	22266	27527
Otu0058	22757	21992	27936
Otu0061	22357	22584	20091
Otu0055	22332	27897	30210
Otu0066	20954	24841	10916
Otu0083	20929	10739	7772
Otu0078	19116	12732	9567
Otu0067	18856	13638	22478
Otu0079	17261	15587	8221
Otu0074	17143	15527	13440
Otu0060	16508	10514	42610
Otu0087	16298	8873	8546
Otu0084	15116	9843	8852
Otu0022	14275	77510	64946
Otu0081	14048	12453	13889
Otu0075	13886	13950	14989
Otu0080	13642	14451	12327
Otu0095	13605	8661	6452
Otu0090	13483	8330	6803
Otu0099	12803	4842	5889
Otu0086	12559	11312	8889
Otu0035	12198	23240	25319
Otu0119	12160	3445	4676
Otu0082	12080	11519	13419
Otu0098	12033	9854	6116
Otu0093	12027	8486	7531
Otu0089	11044	9854	10164
Otu0104	10972	6209	6458
Otu0108	10817	6904	5312
Otu0069	10792	11828	29865
Otu0076	10695	13693	18128
Otu0092	10584	8101	9995
Otu0085	10492	11041	10751

Otu0102	10189	6847	5558
Otu0091	10028	8706	8780
Otu0118	9985	4529	3232
Otu0112	9690	7120	7194
Otu0100	9637	6852	7579
Otu0097	9622	8030	7210
Otu0125	9244	3163	1903
Otu0115	9103	5545	4844
Otu0106	8644	6841	5762
Otu0088	8629	7692	15302
Otu0114	8579	8499	6397
Otu0124	7824	3659	4131
Otu0128	7637	2699	1791
Otu0096	7528	8582	10804
Otu0156	6684	77	2891
Otu0101	6482	6357	11420
Otu0141	6408	1913	1166
Otu0129	6310	2740	3371
Otu0110	6273	4684	9176
Otu0127	6266	3967	3397
Otu0103	6183	7341	8940
Otu0094	6124	7461	12993
Otu0120	5987	4799	5176
Otu0123	5967	5508	3629
Otu0122	5934	3798	3361
Otu0117	5742	5658	8871
Otu0121	5590	3885	4755
Otu0109	5388	6083	11687
Otu0138	5265	2943	2463
Otu0134	5051	3008	3386
Otu0105	5012	7586	9574
Otu0133	4952	2927	2570
Otu0116	4768	4434	7823
Otu0137	4764	5538	3096
Otu0145	4517	4151	3193
Otu0152	4331	2490	1433
Otu0149	4281	1208	859
Otu0135	4264	4469	4470
Otu0126	4226	4014	3839
Otu0161	4211	1547	683
Otu0160	4083	3188	1299
Otu0111	3928	8033	9328
Otu0130	3897	3023	3811
Otu0139	3874	3178	3977
Otu0065	3741	6556	7905
Otu0049	3715	42175	24040
Otu0144	3646	3617	2966

Otu0168	3632	796	512
Otu0146	3453	3624	2524
Otu0171	3080	879	713
Otu0157	2941	1789	2154
Otu0164	2939	1168	1134
Otu0072	2926	6466	8530
Otu0140	2913	2744	2967
Otu0142	2868	3433	2800
Otu0150	2778	1736	2022
Otu0186	2537	1267	642
Otu0148	2493	2494	2418
Otu0151	2418	2560	2081
Otu0169	2403	737	567
Otu0190	2393	709	619
Otu0172	2179	1213	2483
Otu0163	2133	3223	2541
Otu0173	2121	813	1394
Otu0159	2110	2577	2098
Otu0195	1953	331	430
Otu0179	1952	1679	1060
Otu0153	1862	1497	2908
Otu0174	1862	2046	1355
Otu0176	1769	684	680
Otu0191	1735	689	363
Otu0147	1691	2096	3949
Otu0198	1610	366	270
Otu0202	1610	346	220
Otu0155	1539	1735	2403
Otu0196	1536	403	361
Otu0178	1445	1860	1975
Otu0181	1440	879	693
Otu0194	1431	1013	518
Otu0113	1427	1227	1865
Otu0167	1392	1056	932
Otu0154	1361	1609	2176
Otu0162	1330	2397	1953
Otu0175	1299	1608	1907
Otu0166	1290	1532	890
Otu0184	1289	1584	1657
Otu0158	1281	2014	1564
Otu0182	1239	1146	1025
Otu0199	1165	620	360
Otu0185	1062	542	616
Otu0219	1030	654	492
Otu0180	997	579	707
Otu0170	979	1038	1213
Otu0062	939	34614	24131

Otu0183	910	638	735
Otu0193	894	927	649
Otu0206	887	334	492
Otu0177	867	698	750
Otu0209	862	176	80
Otu0214	843	338	664
Otu0200	817	371	384
Otu0218	816	761	142
Otu0212	776	230	162
Otu0187	766	514	434
Otu0189	619	496	532
Otu0228	615	204	271
Otu0235	611	129	75
Otu0044	566	1535	1761
Otu0192	559	522	641
Otu0222	542	70	110
Otu0208	537	389	408
Otu0215	484	185	174
Otu0207	481	292	376
Otu0253	440	263	74
Otu0229	431	299	224
Otu0254	402	229	190
Otu0237	368	378	344
Otu0220	322	150	236
Otu0107	310	12100	10731
Otu0165	298	457	520
Otu0211	269	254	263
Otu0205	268	395	219
Otu0243	262	210	206
Otu0201	259	509	438
Otu0232	259	96	163
Otu0270	255	59	81
Otu0239	241	71	46
Otu0259	241	133	17
Otu0255	231	66	50
Otu0266	230	162	151
Otu0244	225	51	33
Otu0234	220	53	15
Otu0286	214	8	10
Otu0221	211	222	221
Otu0223	207	137	128
Otu0257	196	315	130
Otu0132	195	7537	5388
Otu0213	191	216	290
Otu0249	188	30	93
Otu0246	178	16	25
Otu0143	172	6644	4216

Otu0224	171	212	273
Otu0216	166	322	386
Otu0247	155	53	28
Otu0131	142	7990	4709
Otu0264	141	29	11
Otu0271	140	38	53
Otu0265	138	226	129
Otu0236	127	131	75
Otu0274	124	11	3
Otu0260	119	23	4
Otu0231	118	248	120
Otu0278	110	16	32
Otu0272	109	120	71
Otu0273	104	10	11
Otu0136	102	7438	4920
Otu0288	96	140	39
Otu0226	74	67	578
Otu0287	74	15	32
Otu0361	74	14	0
Otu0245	73	40	118
Otu0227	69	130	377
Otu0230	69	133	114
Otu0225	68	103	202
Otu0240	67	69	54
Otu0269	67	15	3
Otu0304	62	19	65
Otu0250	60	21	14
Otu0248	57	17	21
Otu0317	54	50	10
Otu0277	49	23	6
Otu0188	48	1743	1100
Otu0284	48	12	0
Otu0238	43	297	162
Otu0256	43	97	38
Otu0283	42	6	2
Otu0267	36	156	145
Otu0306	31	20	84
Otu0285	31	4	66
Otu0261	31	33	13
Otu0233	30	99	43
Otu0262	29	7	15
Otu0279	28	4	1
Otu0251	27	42	7
Otu0242	25	232	80
Otu0290	24	1	5
Otu0405	24	0	0
Otu0294	23	2	0

Otu0339	22	9	6
Otu0351	22	39	0
Otu0353	21	7	12
Otu0322	21	44	11
Otu0370	20	1	0
Otu0334	18	0	0
Otu0275	16	41	28
Otu0347	16	0	15
Otu0342	16	12	2
Otu0280	15	30	17
Otu0293	13	12	8
Otu0297	11	75	22
Otu0323	11	21	0
Otu0328	11	0	0
Otu0338	11	0	0
Otu0276	10	3	14
Otu0296	10	5	7
Otu0263	9	32	34
Otu0369	8	0	8
Otu0356	8	2	7
Otu0376	8	0	0
Otu0506	8	0	0
Otu0558	8	0	0
Otu0252	7	62	118
Otu0324	7	0	0
Otu0331	7	0	0
Otu0299	6	0	0
Otu0406	6	0	0
Otu0459	6	0	0
Otu0292	5	75	71
Otu0447	5	2	4
Otu0318	5	18	0
Otu0335	5	4	0
Otu0302	5	0	0
Otu0607	5	0	0
Otu0204	4	1569	792
Otu0410	4	0	3
Otu0545	4	0	0
Otu0557	4	0	0
Otu0303	3	1	0
Otu0305	3	0	0
Otu0321	3	0	0
Otu0456	3	0	0
Otu0384	2	0	13
Otu0386	2	11	1
Otu0441	2	0	0
Otu0561	2	0	0

Otu0751	2	0	0
Otu0312	1	2	0
Otu0366	1	1	0
Otu0346	1	0	0
Otu0358	1	0	0
Otu0439	1	0	0
Otu0452	1	0	0
Otu0391	0	0	53
Otu0426	0	0	51
Otu0381	0	0	47
Otu0390	0	2	36
Otu0291	0	52	35
Otu0282	0	116	32
Otu0330	0	16	32
Otu0307	0	45	26
Otu0289	0	0	21
Otu0387	0	1	20
Otu0313	0	36	17
Otu0337	0	0	16
Otu0570	0	0	16
Otu0596	0	0	16
Otu0343	0	3	14
Otu0626	0	0	13
Otu0638	0	0	11
Otu0476	0	13	9
Otu0421	0	0	9
Otu0372	0	0	8
Otu0515	0	0	8
Otu0571	0	0	8
Otu0523	0	0	6
Otu0298	0	14	5
Otu0501	0	8	5
Otu0472	0	0	5
Otu0503	0	0	5
Otu0738	0	0	5
Otu0348	0	40	3
Otu0412	0	22	3
Otu0492	0	5	3
Otu0392	0	0	3
Otu0310	0	66	2
Otu0295	0	7	2
Otu0466	0	0	2
Otu0494	0	0	2
Otu0500	0	0	2
Otu0835	0	0	2
Otu0382	0	31	1
Otu0378	0	0	1

Otu0432	0	0	1
Otu0665	0	0	1
Otu0730	0	0	1
Otu0742	0	0	1
Otu0920	0	0	1
Otu0333	0	40	0
Otu0485	0	32	0
Otu0301	0	25	0
Otu0360	0	23	0
Otu0428	0	22	0
Otu0451	0	22	0
Otu0444	0	21	0
Otu0454	0	21	0
Otu0435	0	20	0
Otu0551	0	18	0
Otu0425	0	17	0
Otu0588	0	17	0
Otu0593	0	16	0
Otu0608	0	15	0
Otu0448	0	14	0
Otu0534	0	11	0
Otu0460	0	10	0
Otu0606	0	10	0
Otu0667	0	9	0
Otu0464	0	6	0
Otu0541	0	6	0
Otu0677	0	6	0
Otu0718	0	6	0
Otu0722	0	6	0
Otu0401	0	5	0
Otu0434	0	5	0
Otu0595	0	4	0
Otu0611	0	4	0
Otu0781	0	4	0
Otu0578	0	3	0
Otu0764	0	3	0
Otu0540	0	2	0
Otu0727	0	2	0
Otu0865	0	2	0
Otu0352	0	1	0
Otu0484	0	1	0
Otu0659	0	1	0
Otu0683	0	1	0
