# Supplementary material for: Oral Vaccination Using a Probiotic Vaccine Platform Combined with Prebiotics Impacts Immune Response and the Microbiome
Source: Vaccines (Basel). 2022 Sep 4;10(9):1465. doi: 10.3390/vaccines10091465 (PMC9504555; doi:10.3390/vaccines10091465)
Supplement: Supplementary file 1 [file vaccines-10-01465-s001.zip › Table_S7_negative_importance-fecal.pdf]

Table_S7_negative_importance-fecal

	kingdom	phylum	class	order	family	genus	neg_a_4
Otu0023	Bacteria	Firmicutes	Clostridia	Clostridiale	Lachnospira	Lachnospira	1
Otu0109	Bacteria	Firmicutes	Clostridia	Clostridiale	Lachnospira	uncultured	0.854053
Otu0048	Bacteria	Firmicutes	Clostridia	Clostridiale	Lachnospira	Acetatifact	0.539027
Otu0064	Bacteria	Firmicutes	Erysipelotri	Erysipelotri	Erysipelotri	Turicibacte	0.512364
Otu0058	Bacteria	Firmicutes	Clostridia	Clostridiale	Lachnospira	uncultured	0.359442
Otu0089	Bacteria	Firmicutes	Clostridia	Clostridiale	Ruminococ	Anaerotrur	0.309309
Otu0040	Bacteria	Firmicutes	Clostridia	Clostridiale	Lachnospira	Lachnospira	0.240638
Otu0111	Bacteria	Firmicutes	Clostridia	Clostridiale	Lachnospira	Lachnospira	0.233953
Otu0057	Bacteria	Firmicutes	Clostridia	Clostridiale	Lachnospira	uncultured	0.202801
Otu0009	Bacteria	Firmicutes	Clostridia	Clostridiale	Clostridiace	Clostridium	0.196545
Otu0013	Bacteria	Firmicutes	Clostridia	Clostridiale	Lachnospira	Lachnospira	0.190137
Otu0015	Bacteria	Firmicutes	Clostridia	Clostridiale	Lachnospira	Roseburia	0.187285
Otu0061	Bacteria	Firmicutes	Clostridia	Clostridiale	Lachnospira	Lachnospira	0.185688
Otu0037	Bacteria	Firmicutes	Clostridia	Clostridiale	Lachnospira	Lachnospira	0.178223
Otu0076	Bacteria	Firmicutes	Clostridia	Clostridiale	Lachnospira	uncultured	0.147698
Otu0010	Bacteria	Firmicutes	Clostridia	Clostridiale	Lachnospira	Lachnospira	0.131539
Otu0047	Bacteria	Firmicutes	Clostridia	Clostridiale	Lachnospira	Lachnospira	0.127867
Otu0157	Bacteria	Firmicutes	Clostridia	Clostridiale	Ruminococ	Oscillibacte	0.127195
Otu0087	Bacteria	Firmicutes	Bacilli	Lactobacilla	Streptococ	Lactococcu	0.120524
Otu0097	Bacteria	Firmicutes	Clostridia	Clostridiale	Lachnospira	GCA-90006	0.119339
Otu0026	Bacteria	Firmicutes	Clostridia	Clostridiale	Lachnospira	Lachnospira	0.094823
Otu0153	Bacteria	Firmicutes	Clostridia	Clostridiale	Ruminococ	Ruminococ	0.09016
Otu0096	Bacteria	Firmicutes	Clostridia	Clostridiale	Lachnospira	uncultured	0.089982
Otu0042	Bacteria	Firmicutes	Clostridia	Clostridiale	Lachnospira	Lachnospira	0.08808
Otu0071	Bacteria	Firmicutes	Clostridia	Clostridiale	Ruminococ	Ruminococ	0.079808
Otu0075	Bacteria	Firmicutes	Clostridia	Clostridiale	Lachnospira	Lachnospira	0.078068
Otu0038	Bacteria	Firmicutes	Clostridia	Clostridiale	Lachnospira	Lachnospira	0.072773
Otu0100	Bacteria	Firmicutes	Clostridia	Clostridiale	Lachnospira	uncultured	0.068615
Otu0011	Bacteria	Firmicutes	Clostridia	Clostridiale	Lachnospira	Lachnospira	0.066798
Otu0091	Bacteria	Firmicutes	Clostridia	Clostridiale	Lachnospira	Lachnospira	0.066765
Otu0022	Bacteria	Proteobact	Gammaprc	Enterobact	Enterobact	Escherichia	0.06638
Otu0051	Bacteria	Firmicutes	Clostridia	Clostridiale	Ruminococ	Ruminococ	0.066014
Otu0068	Bacteria	Firmicutes	Clostridia	Clostridiale	Lachnospira	Lachnospira	0.064522
Otu0012	Bacteria	Firmicutes	Clostridia	Clostridiale	Lachnospira	Lachnospira	0.055872
Otu0139	Bacteria	Firmicutes	Clostridia	Clostridiale	Lachnospira	Lachnospira	0.052349
Otu0020	Bacteria	Firmicutes	Clostridia	Clostridiale	Lachnospira	Lachnospira	0.051168
Otu0016	Bacteria	Firmicutes	Clostridia	Clostridiale	Lachnospira	Lachnospira	0.050137
Otu0053	Bacteria	Firmicutes	Clostridia	Clostridiale	Lachnospira	Lachnospira	0.049899
Otu0060	Bacteria	Firmicutes	Clostridia	Clostridiale	Peptostrep	Romboutsi	0.049764
Otu0155	Bacteria	Firmicutes	Clostridia	Clostridiale	Ruminococ	Anaerotrur	0.046435
Otu0117	Bacteria	Firmicutes	Clostridia	Clostridiale	Ruminococ	Ruminiclos	0.045463
Otu0184	Bacteria	Firmicutes	Clostridia	Clostridiale	Lachnospira	Lachnospira	0.044633
Otu0025	Bacteria	Firmicutes	Clostridia	Clostridiale	Lachnospira	Lachnospira	0.040837
Otu0088	Bacteria	Firmicutes	Clostridia	Clostridiale	Ruminococ	Butyricicoc	0.04024
Otu0154	Bacteria	Firmicutes	Clostridia	Clostridiale	Lachnospira	Lachnospira	0.039949

Otu0024	Bacteria	Firmicutes	Clostridia	Clostridiale	Ruminococ	Ruminococ	0.03955
Otu0006	Bacteria	Firmicutes	Clostridia	Clostridiale	Lachnospira	Lachnospira	0.03675
Otu0101	Bacteria	Firmicutes	Clostridia	Clostridiale	Lachnospira	ASF356	0.036317
Otu0183	Bacteria	Firmicutes	Clostridia	Clostridiale	Ruminococ	Ruminiclos	0.036217
Otu0033	Bacteria	Firmicutes	Clostridia	Clostridiale	Lachnospira	A2	0.034219
Otu0086	Bacteria	Firmicutes	Clostridia	Clostridiale	Lachnospira	Lachnospira	0.033504
Otu0216	Bacteria	Firmicutes	Clostridia	Clostridiale	Lachnospira	Lachnospira	0.033114
Otu0003	Bacteria	Firmicutes	Clostridia	Clostridiale	Lachnospira	Lachnospira	0.033084
Otu0148	Bacteria	Firmicutes	Clostridia	Clostridiale	Lachnospira	Lachnospira	0.031887
Otu0081	Bacteria	Firmicutes	Clostridia	Clostridiale	Lachnospira	Lachnoclos	0.029982
Otu0027	Bacteria	Firmicutes	Clostridia	Clostridiale	Lachnospira	Acetatifact	0.0289
Otu0128	Bacteria	Firmicutes	Clostridia	Clostridiale	Family_XIII	Family_XIII_	0.028485
Otu0052	Bacteria	Firmicutes	Clostridia	Clostridiale	Lachnospira	Lachnospira	0.027192
Otu0055	Bacteria	Firmicutes	Clostridia	Clostridiale	Lachnospira	A2	0.027132
Otu0049	Bacteria	Firmicutes	Bacilli	Bacillales	Bacillaceae	Bacillus	0.026374
Otu0121	Bacteria	Firmicutes	Clostridia	Clostridiale	Lachnospira	Lachnospira	0.025713
Otu0074	Bacteria	Firmicutes	Clostridia	Clostridiale	Lachnospira	Lachnospira	0.024904
Otu0028	Bacteria	Firmicutes	Clostridia	Clostridiale	Lachnospira	Lachnospira	0.024221
Otu0095	Bacteria	Firmicutes	Clostridia	Clostridiale	Lachnospira	Lachnospira	0.024135
Otu0140	Bacteria	Firmicutes	Clostridia	Clostridiale	Lachnospira	Lachnospira	0.021971
Otu0014	Bacteria	Firmicutes	Clostridia	Clostridiale	Lachnospira	Lachnospira	0.021512
Otu0135	Bacteria	Firmicutes	Clostridia	Clostridiale	Lachnospira	Lachnospira	0.021181
Otu0142	Bacteria	Firmicutes	Clostridia	Clostridiale	Lachnospira	Lachnospira	0.02114
Otu0001	Bacteria	Bacteroide	Bacteroidia	Bacteroidal	Muribacula	Muribacula	0.020931
Otu0114	Bacteria	Firmicutes	Clostridia	Clostridiale	Lachnospira	Lachnospira	0.020096
Otu0178	Bacteria	Firmicutes	Clostridia	Clostridiale	Lachnospira	Lachnospira	0.019409
Otu0041	Bacteria	Firmicutes	Clostridia	Clostridiale	Lachnospira	Lachnoclos	0.01886
Otu0066	Bacteria	Firmicutes	Clostridia	Clostridiale	Lachnospira	Lachnospira	0.018802
Otu0173	Bacteria	Firmicutes	Clostridia	Clostridiale	Ruminococ	Ruminococ	0.017874
Otu0034	Bacteria	Firmicutes	Clostridia	Clostridiale	Lachnospira	Acetatifact	0.016435
Otu0032	Bacteria	Firmicutes	Clostridia	Clostridiale	Ruminococ	Ruminiclos	0.016376
Otu0070	Bacteria	Firmicutes	Erysipelotri	Erysipelotri	Erysipelotri	Erysipelato	0.015416
Otu0146	Bacteria	Firmicutes	Clostridia	Clostridiale	Lachnospira	Lachnospira	0.013952
Otu0077	Bacteria	Firmicutes	Clostridia	Clostridiale	Ruminococ	Ruminiclos	0.01349
Otu0039	Bacteria	Firmicutes	Clostridia	Clostridiale	Lachnospira	Lachnospira	0.013063
Otu0031	Bacteria	Firmicutes	Clostridia	Clostridiale	Lachnospira	Marvinbrya	0.012507
Otu0122	Bacteria	Firmicutes	Clostridia	Clostridiale	Ruminococ	Ruminiclos	0.012463
Otu0062	Bacteria	Proteobact	Alphaprote	Rhizobiales	Rhizobiacea	Rhizobiacea	0.012456
Otu0050	Bacteria	Firmicutes	Clostridia	Clostridiale	Ruminococ	Oscillibacte	0.012282
Otu0090	Bacteria	Firmicutes	Clostridia	Clostridiale	Ruminococ	Ruminococ	0.012055
Otu0134	Bacteria	Firmicutes	Clostridia	Clostridiale	Lachnospira	Lachnospira	0.011866
Otu0166	Bacteria	Firmicutes	Clostridia	Clostridiale	Ruminococ	Oscillibacte	0.011693
Otu0078	Bacteria	Firmicutes	Clostridia	Clostridiale	Ruminococ	Ruminococ	0.011239
Otu0099	Bacteria	Actinobact	Coriobacte	Coriobacte	Eggerthella	Adlercreutz	0.011224
Otu0019	Bacteria	Firmicutes	Clostridia	Clostridiale	Ruminococ	Ruminiclos	0.011141
Otu0193	Bacteria	Firmicutes	Clostridia	Clostridiale	Ruminococ	Ruminiclos	0.010921
Otu0054	Bacteria	Firmicutes	Clostridia	Clostridiale	Lachnospira	Lachnospira	0.010178

Otu0107	Bacteria	Proteobact	Alphaprote	Rhizobiales	Rhizobiacea	Brucella	0.009612
Otu0174	Bacteria	Firmicutes	Clostridia	Clostridiale	Lachnospira	Lachnospira	0.009497
Otu0147	Bacteria	Firmicutes	Clostridia	Clostridiale	Lachnospira	Lachnospira	0.008697
Otu0190	Bacteria	Firmicutes	Clostridia	Clostridiale	Lachnospira	Lachnospira	0.008621
Otu0131	Bacteria	Proteobact	Alphaprote	Caulobacte	Caulobacte	Brevundim	0.008094
Otu0182	Bacteria	Firmicutes	Clostridia	Clostridiale	Lachnospira	Lachnospira	0.007964
Otu0056	Bacteria	Firmicutes	Clostridia	Clostridiale	Lachnospira	uncultured	0.007864
Otu0045	Bacteria	Firmicutes	Bacilli	Bacillales	Staphyloco	Staphyloco	0.007826
Otu0227	Bacteria	Firmicutes	Clostridia	Clostridiale	Ruminococ	Ruminococ	0.007397
Otu0207	Bacteria	Firmicutes	Clostridia	Clostridiale	Lachnospira	Lachnospira	0.007026
Otu0069	Bacteria	Firmicutes	Clostridia	Clostridiale	Lachnospira	uncultured	0.006931
Otu0163	Bacteria	Firmicutes	Clostridia	Clostridiale	Lachnospira	Lachnospira	0.006724
Otu0126	Bacteria	Firmicutes	Clostridia	Clostridiale	Lachnospira	Lachnospira	0.006385
Otu0079	Bacteria	Firmicutes	Clostridia	Clostridiale	Lachnospira	Lachnospira	0.006147
Otu0002	Bacteria	Bacteroide	Bacteroidia	Bacteroidal	Muribacula	Muribacula	0.006057
Otu0145	Bacteria	Firmicutes	Clostridia	Clostridiale	Lachnospira	Lachnospira	0.006055
Otu0104	Bacteria	Firmicutes	Clostridia	Clostridiale	Lachnospira	Tyzzerella	0.006034
Otu0168	Bacteria	Firmicutes	Clostridia	Clostridiale	Clostridiale	Clostridiale	0.005993
Otu0098	Bacteria	Firmicutes	Clostridia	Clostridiale	Ruminococ	Ruminococ	0.005969
Otu0004	Bacteria	Firmicutes	Bacilli	Lactobacilla	Lactobacilla	Lactobacillu	0.005937
Otu0029	Bacteria	Firmicutes	Clostridia	Clostridiale	Lachnospira	Lachnospira	0.00569
Otu0225	Bacteria	Firmicutes	Clostridia	Clostridiale	Family_XIII	Family_XIII_	0.00548
Otu0123	Bacteria	Firmicutes	Clostridia	Clostridiale	Lachnospira	Lachnospira	0.004995
Otu0083	Bacteria	Firmicutes	Clostridia	Clostridiale	Ruminococ	Ruminococ	0.00481
Otu0085	Bacteria	Firmicutes	Clostridia	Clostridiale	Lachnospira	Lachnospira	0.004773
Otu0214	Bacteria	Firmicutes	Clostridia	Clostridiale	Lachnospira	Lachnospira	0.004762
Otu0133	Bacteria	Firmicutes	Clostridia	Clostridiale	Ruminococ	Ruminococ	0.004689
Otu0209	Bacteria	Firmicutes	Clostridia	Clostridiale	Peptococca	uncultured	0.004573
Otu0162	Bacteria	Firmicutes	Clostridia	Clostridiale	Lachnospira	Lachnospira	0.004482
Otu0248	Bacteria	Firmicutes	Clostridia	Clostridiale	Ruminococ	Ruminococ	0.004286
Otu0189	Bacteria	Firmicutes	Clostridia	Clostridiale	Lachnospira	Lachnospira	0.00397
Otu0018	Bacteria	Firmicutes	Clostridia	Clostridiale	Ruminococ	Ruminococ	0.003894
Otu0205	Bacteria	Firmicutes	Clostridia	Clostridiale	Ruminococ	Ruminococ	0.003879
Otu0035	Bacteria	Proteobact	Gammapro	Enterobact	Enterobact	Enterobact	0.003805
Otu0233	Bacteria	Firmicutes	Clostridia	Clostridiale	Lachnospira	Lachnospira	0.003531
Otu0202	Bacteria	Firmicutes	Clostridia	Clostridiale	Clostridiale	Clostridiale	0.003491
Otu0165	Bacteria	Firmicutes	Bacilli	Bacillales	Bacillaceae	Bacillus	0.003284
Otu0243	Bacteria	Firmicutes	Clostridia	Clostridiale	Lachnospira	Lachnospira	0.003273
Otu0172	Bacteria	Firmicutes	Clostridia	Clostridiale	Lachnospira	Lachnospira	0.003265
Otu0229	Bacteria	Firmicutes	Clostridia	Clostridiale	Lachnospira	Lachnospira	0.003182
Otu0149	Bacteria	Firmicutes	Clostridia	Clostridiale	Ruminococ	Ruminococ	0.003015
Otu0270	Bacteria	Firmicutes	Clostridia	Clostridiale	Lachnospira	Lachnospira	0.002782
Otu0137	Bacteria	Firmicutes	Clostridia	Clostridiale	Lachnospira	Lachnospira	0.002709
Otu0118	Bacteria	Firmicutes	Clostridia	Clostridiale	Clostridiale	Clostridiale	0.002533
Otu0138	Bacteria	Firmicutes	Clostridia	Clostridiale	Ruminococ	Ruminococ	0.00233
Otu0224	Bacteria	Firmicutes	Clostridia	Clostridiale	Lachnospira	Lachnospira	0.002021
Otu0152	Bacteria	Tenericutes	Mollicutes	Mollicutes_	Mollicutes_	Mollicutes_	0.001973

Otu0161	Bacteria	Firmicutes	Clostridia	Clostridiale	Clostridiale	Clostridiale	0.001796
Otu0120	Bacteria	Firmicutes	Clostridia	Clostridiale	Ruminococ	Ruminococ	0.001723
Otu0186	Bacteria	Firmicutes	Clostridia	Clostridiale	Lachnospira	Lachnospira	0.001637
Otu0171	Bacteria	Firmicutes	Clostridia	Clostridiale	Ruminococ	Ruminococ	0.001594
Otu0221	Bacteria	Firmicutes	Clostridia	Clostridiale	Ruminococ	GCA-90006	0.001546
Otu0272	Bacteria	Firmicutes	Clostridia	Clostridiale	Ruminococ	Ruminococ	0.001546
Otu0228	Bacteria	Tenericutes	Mollicutes	Mollicutes_	Mollicutes_	Mollicutes_	0.001464
Otu0232	Bacteria	Firmicutes	Clostridia	Clostridiale	Ruminococ	Ruminococ	0.001464
Otu0292	Bacteria	Firmicutes	Clostridia	Clostridiale	Lachnospira	Lachnospira	0.001464
Otu0115	Bacteria	Firmicutes	Clostridia	Clostridiale	Lachnospira	Tyzzerella_	0.00146
Otu0160	Bacteria	Firmicutes	Clostridia	Clostridiale	Ruminococ	Ruminococ	0.001391
Otu0191	Bacteria	Firmicutes	Clostridia	Clostridiale	Clostridiale	Clostridiale	0.001391
Otu0222	Bacteria	Firmicutes	Erysipelotri	Erysipelotri	Erysipelotri	Candidatus	0.001325
Otu0169	Bacteria	Firmicutes	Erysipelotri	Erysipelotri	Erysipelotri	Erysipelotri	0.001325
Otu0007	Bacteria	Firmicutes	Clostridia	Clostridiale	Ruminococ	Ruminiclos	0.001192
Otu0212	Bacteria	Firmicutes	Clostridia	Clostridiale	Ruminococ	Ruminococ	0.001159
Otu0180	Bacteria	Firmicutes	Clostridia	Clostridiale	Ruminococ	Oscillibacte	0.001146
Otu0143	Bacteria	Proteobact	Gammapro	Xanthomor	Xanthomor	Stenotroph	0.000896
Otu0141	Bacteria	Actinobact	Coriobacte	Coriobacte	Eggerthella	Eggerthella	0.000891
Otu0132	Bacteria	Actinobact	Actinobact	Micrococca	Micrococca	Micrococca	0.00022
Otu0017	Bacteria	Firmicutes	Bacilli	Lactobacilla	Lactobacilla	Lactobacillu	8.84E-05
Otu0179	Bacteria	Firmicutes	Clostridia	Clostridiale	Lachnospira	Lachnospira	1.82E-05
Otu0113	Bacteria	Firmicutes	Bacilli	Lactobacilla	Lactobacilla	Lactobacillu	0
Otu0156	Bacteria	Actinobact	Actinobact	Micrococca	Microbacte	Curtobacte	0
Otu0185	Bacteria	Firmicutes	Clostridia	Clostridiale	Ruminococ	Ruminococ	0
Otu0194	Bacteria	Firmicutes	Clostridia	Clostridiale	Lachnospira	Lachnospira	0
Otu0206	Bacteria	Firmicutes	Clostridia	Clostridiale	Ruminococ	Ruminococ	0
Otu0213	Bacteria	Firmicutes	Clostridia	Clostridiale	Clostridiale	Clostridiale	0
Otu0215	Bacteria	Tenericutes	Mollicutes	Mollicutes_	Mollicutes_	Mollicutes_	0
Otu0219	Bacteria	Verrucomic	Verrucomic	Verrucomic	Akkermans	Akkermans	0
Otu0220	Bacteria	Firmicutes	Clostridia	Clostridiale	Lachnospira	Lachnospira	0
Otu0223	Bacteria	Firmicutes	Clostridia	Clostridiale	Lachnospira	Lachnospira	0
Otu0234	Bacteria	Bacteroide	Bacteroidia	Bacteroidal	Muribacula	Muribacula	0
Otu0235	Bacteria	Firmicutes	Clostridia	Clostridiale	Clostridiale	Clostridiale	0
Otu0237	Bacteria	Firmicutes	Clostridia	Clostridiale	Lachnospira	Lachnospira	0
Otu0239	Bacteria	Firmicutes	Clostridia	Clostridiale	Ruminococ	Ruminococ	0
Otu0240	Bacteria	Firmicutes	Clostridia	Clostridiale	Lachnospira	Lachnospira	0
Otu0242	Bacteria	Proteobact	Gammapro	Betaprotec	Burkholder	Ralstonia	0
Otu0244	Bacteria	Firmicutes	Clostridia	Clostridiale	Clostridiale	Clostridiale	0
Otu0246	Bacteria	Firmicutes	Clostridia	Clostridiale	Family_XIII	Family_XIII_	0
Otu0247	Bacteria	Firmicutes	Clostridia	Clostridiale	Clostridiale	Clostridiale	0
Otu0249	Bacteria	Firmicutes	Clostridia	Clostridiale	Ruminococ	Ruminococ	0
Otu0250	Bacteria	Firmicutes	Clostridia	Clostridiale	Lachnospira	Lachnospira	0
Otu0251	Bacteria	Firmicutes	Clostridia	Clostridiale	Ruminococ	Ruminiclos	0
Otu0252	Bacteria	Firmicutes	Clostridia	Clostridiale	Lachnospira	Lachnospira	0
Otu0253	Bacteria	Firmicutes	Clostridia	Clostridiale	Clostridiale	Clostridiale	0
Otu0254	Bacteria	Firmicutes	Clostridia	Clostridiale	Lachnospira	Lachnospira	0

Otu0256	Bacteria	Firmicutes	Clostridia	Clostridiale	Lachnospir	Lachnospira	0
Otu0259	Bacteria	Tenericutes	Mollicutes	Mollicutes_	Mollicutes_	Mollicutes_	0
Otu0260	Bacteria	Firmicutes	Firmicutes_	Firmicutes_	Firmicutes_	Firmicutes_	0
Otu0261	Bacteria	Firmicutes	Clostridia	Clostridiale	Lachnospir	Lachnospira	0
Otu0262	Bacteria	Firmicutes	Clostridia	Clostridiale	Ruminococ	Ruminiclos	0
Otu0263	Bacteria	Firmicutes	Clostridia	Clostridiale	Family_XIII	Family_XIII_	0
Otu0264	Bacteria	Firmicutes	Clostridia	Clostridiale	Clostridiale	Clostridiale	0
Otu0265	Bacteria	Firmicutes	Clostridia	Clostridiale	Lachnospir	Tyzzerella_	0
Otu0266	Bacteria	Firmicutes	Clostridia	Clostridiale	Ruminococ	Ruminococ	0
Otu0267	Bacteria	Firmicutes	Bacilli	Lactobacilla	Lactobacilla	Lactobacillu	0
Otu0269	Bacteria	Bacteroide	Bacteroidia	Bacteroidal	Muribacula	Muribacula	0
Otu0271	Bacteria	Firmicutes	Clostridia	Clostridiale	Clostridiale	Clostridiale	0
Otu0273	Bacteria	Firmicutes	Clostridia	Clostridiale	Clostridiale	Clostridiale	0
Otu0274	Bacteria	Firmicutes	Clostridia	Clostridiale	Clostridiale	Clostridiale	0
Otu0276	Bacteria	Firmicutes	Clostridia	Clostridiale	Ruminococ	Ruminococ	0
Otu0277	Bacteria	Firmicutes	Clostridia	Clostridiale	Lachnospir	Lachnospira	0
Otu0278	Bacteria	Actinobact	Coriobacte	Coriobacte	Eggerthella	Eggerthella	0
Otu0279	Bacteria	Bacteroide	Bacteroidia	Bacteroidal	Muribacula	Muribacula	0
Otu0280	Bacteria	Firmicutes	Clostridia	Clostridiale	Lachnospir	Lachnospira	0
Otu0282	Bacteria	Actinobact	Actinobact	Micrococca	Brevibacter	Brevibacter	0
Otu0283	Bacteria	Bacteroide	Bacteroidia	Bacteroidal	Muribacula	Muribacula	0
time	NA	NA	NA	NA	NA	NA	0
Otu0285	Bacteria	Proteobact	Alphaprote	Rhizobiales	Beijerinckia	Methyloba	0
Otu0286	Bacteria	Firmicutes	Clostridia	Clostridiale	Ruminococ	Ruminococ	0
Otu0287	Bacteria	Firmicutes	Clostridia	Clostridiale	Ruminococ	Butyricicoc	0
Otu0288	Bacteria	Firmicutes	Clostridia	Clostridiale	Lachnospir	Lachnospira	0
Otu0289	Bacteria	Firmicutes	Clostridia	Clostridiale	Lachnospir	Lachnospira	0
Otu0290	Bacteria	Firmicutes	Clostridia	Clostridiale	Clostridiale	Clostridiale	0
Otu0291	Bacteria	Proteobact	Alphaprote	Rhizobiales	Beijerinckia	Methyloba	0
Otu0293	Bacteria	Tenericutes	Mollicutes	Mollicutes_	Mollicutes_	Mollicutes_	0
NA.1	NA	NA	NA	NA	NA	NA	0
Otu0295	Bacteria	Firmicutes	Clostridia	Clostridiale	Lachnospir	Lachnoclos	0
Otu0296	Bacteria	Firmicutes	Clostridia	Clostridiale	Ruminococ	Ruminococ	0
Otu0297	Bacteria	Proteobact	Gammapro	Pseudomo	Pseudomo	Pseudomo	0
Otu0298	Bacteria	Firmicutes	Clostridia	Clostridiale	Lachnospir	Lachnospira	0
NA.2	NA	NA	NA	NA	NA	NA	0
NA.3	NA	NA	NA	NA	NA	NA	0
NA.4	NA	NA	NA	NA	NA	NA	0
NA.5	NA	NA	NA	NA	NA	NA	0
Otu0304	Bacteria	Firmicutes	Clostridia	Clostridiale	Ruminococ	Flavonifrac	0
NA.6	NA	NA	NA	NA	NA	NA	0
Otu0306	Bacteria	Firmicutes	Clostridia	Clostridiale	Lachnospir	Lachnospira	0
Otu0307	Bacteria	Firmicutes	Bacilli	Bacillales	Paenibacilla	Paenibacillu	0
Otu0310	Bacteria	Proteobact	Alphaprote	Rhizobiales	Labraceae	Labrys	0
NA.7	NA	NA	NA	NA	NA	NA	0
Otu0313	Bacteria	Firmicutes	Clostridia	Clostridiale	Lachnospir	Lachnoclos	0
Otu0317	Bacteria	Firmicutes	Clostridia	Clostridiale	Lachnospir	Lachnospira	0

NA.8	NA	NA	NA	NA	NA	NA	0
NA.9	NA	NA	NA	NA	NA	NA	0
Otu0322	Bacteria	Firmicutes	Clostridia	Clostridiale	Lachnospir	Lachnospir	0
NA.10	NA	NA	NA	NA	NA	NA	0
NA.11	NA	NA	NA	NA	NA	NA	0
NA.12	NA	NA	NA	NA	NA	NA	0
Otu0330	Bacteria	Proteobact	Alphaprote	Rhizobiales	Rhizobiacea	Phyllobacte	0
NA.13	NA	NA	NA	NA	NA	NA	0
NA.14	NA	NA	NA	NA	NA	NA	0
NA.15	NA	NA	NA	NA	NA	NA	0
NA.16	NA	NA	NA	NA	NA	NA	0
Otu0337	Bacteria	Firmicutes	Clostridia	Clostridiale	Lachnospir	Lachnospir	0
NA.17	NA	NA	NA	NA	NA	NA	0
Otu0339	Bacteria	Firmicutes	Clostridia	Clostridiale	Lachnospir	Acetatifact	0
Otu0342	Bacteria	Firmicutes	Clostridia	Clostridiale	Clostridiale	Clostridiale	0
Otu0343	Bacteria	Firmicutes	Clostridia	Clostridiale	Lachnospir	Lachnospir	0
NA.18	NA	NA	NA	NA	NA	NA	0
Otu0347	Bacteria	Firmicutes	Clostridia	Clostridiale	Lachnospir	Lachnospir	0
Otu0348	Bacteria	Proteobact	Gammaprc	Gammaprc	Unknown_	Acidibacter	0
NA.19	NA	NA	NA	NA	NA	NA	0
NA.20	NA	NA	NA	NA	NA	NA	0
Otu0353	Bacteria	Firmicutes	Clostridia	Clostridiale	Ruminococ	Ruminiclos	0
Otu0356	Bacteria	Firmicutes	Clostridia	Clostridiale	Lachnospir	Lachnospir	0
NA.21	NA	NA	NA	NA	NA	NA	0
NA.22	NA	NA	NA	NA	NA	NA	0
NA.23	NA	NA	NA	NA	NA	NA	0
NA.24	NA	NA	NA	NA	NA	NA	0
Otu0369	Bacteria	Firmicutes	Clostridia	Clostridiale	Ruminococ	Ruminococ	0
NA.25	NA	NA	NA	NA	NA	NA	0
Otu0372	Bacteria	Firmicutes	Bacilli	Lactobacilla	Streptococ	Streptococ	0
NA.26	NA	NA	NA	NA	NA	NA	0
Otu0378	Bacteria	Firmicutes	Clostridia	Clostridiale	Ruminococ	Ruminiclos	0
Otu0381	Bacteria	Firmicutes	Clostridia	Clostridiale	Family_XI	Finegoldia	0
Otu0382	Bacteria	Proteobact	Alphaprote	Rhizobiales	Xanthobact	Rhodopseu	0
Otu0384	Bacteria	Proteobact	Gammaprc	Pseudomoi	Moraxellac	Acinetobac	0
Otu0386	Bacteria	Tenericutes	Mollicutes	Mollicutes_	Mollicutes_	Mollicutes_	0
Otu0387	Bacteria	Proteobact	Gammaprc	Betaprotec	Burkholder	Janthinoba	0
Otu0390	Bacteria	Bacteroide	Bacteroidia	Bacteroidal	Prevotellac	Prevotella_	0
Otu0391	Bacteria	Proteobact	Gammaprc	Pseudomoi	Moraxellac	Acinetobac	0
Otu0392	Bacteria	Proteobact	Alphaprote	Sphingomo	Sphingomo	Sphingomo	0
NA.27	NA	NA	NA	NA	NA	NA	0
NA.28	NA	NA	NA	NA	NA	NA	0
NA.29	NA	NA	NA	NA	NA	NA	0
Otu0410	Bacteria	Firmicutes	Clostridia	Clostridiale	Ruminococ	Ruminococ	0
Otu0412	Bacteria	Firmicutes	Clostridia	Clostridiale	Lachnospir	Lachnospir	0
Otu0421	Bacteria	Actinobact	Actinobact	Corynebact	Corynebact	Corynebact	0
NA.30	NA	NA	NA	NA	NA	NA	0

Otu0426	Bacteria	Firmicutes	Clostridia	Clostridiale	Ruminococ	Subdoligra	0
NA.31	NA	NA	NA	NA	NA	NA	0
Otu0432	Bacteria	Firmicutes	Clostridia	Clostridiale	Lachnospir	Lachnoclos	0
NA.32	NA	NA	NA	NA	NA	NA	0
NA.33	NA	NA	NA	NA	NA	NA	0
NA.34	NA	NA	NA	NA	NA	NA	0
NA.35	NA	NA	NA	NA	NA	NA	0
NA.36	NA	NA	NA	NA	NA	NA	0
Otu0447	Bacteria	Tenericutes	Mollicutes	Mollicutes_	Mollicutes_	Mollicutes_	0
NA.37	NA	NA	NA	NA	NA	NA	0
NA.38	NA	NA	NA	NA	NA	NA	0
NA.39	NA	NA	NA	NA	NA	NA	0
NA.40	NA	NA	NA	NA	NA	NA	0
NA.41	NA	NA	NA	NA	NA	NA	0
NA.42	NA	NA	NA	NA	NA	NA	0
NA.43	NA	NA	NA	NA	NA	NA	0
NA.44	NA	NA	NA	NA	NA	NA	0
Otu0466	Bacteria	Firmicutes	Clostridia	Clostridiale	Lachnospir	Lachnospir	0
Otu0472	Bacteria	Proteobact	Gammapro	Betaprotec	Burkholder	Burkholder	0
Otu0476	Bacteria	Bacteroide	Bacteroidia	Sphingobac	Sphingobac	Sphingobac	0
NA.45	NA	NA	NA	NA	NA	NA	0
NA.46	NA	NA	NA	NA	NA	NA	0
Otu0492	Bacteria	Proteobact	Gammapro	Pseudomo	Moraxellac	Enhydroba	0
Otu0494	Bacteria	Firmicutes	Clostridia	Clostridiale	Lachnospir	Lachnospir	0
Otu0500	Bacteria	Actinobact	Actinobact	Micrococca	Intrasporar	Janibacter	0
Otu0501	Bacteria	Proteobact	Alphaprote	Sphingomo	Sphingomo	Sphingomo	0
Otu0503	Bacteria	Proteobact	Alphaprote	Sphingomo	Sphingomo	Sphingomo	0
NA.47	NA	NA	NA	NA	NA	NA	0
Otu0515	Bacteria	Proteobact	Gammapro	Betaprotec	Burkholder	Burkholder	0
Otu0523	Bacteria	Firmicutes	Clostridia	Clostridiale	Clostridiale	Clostridiale	0
NA.48	NA	NA	NA	NA	NA	NA	0
NA.49	NA	NA	NA	NA	NA	NA	0
NA.50	NA	NA	NA	NA	NA	NA	0
NA.51	NA	NA	NA	NA	NA	NA	0
NA.52	NA	NA	NA	NA	NA	NA	0
NA.53	NA	NA	NA	NA	NA	NA	0
NA.54	NA	NA	NA	NA	NA	NA	0
NA.55	NA	NA	NA	NA	NA	NA	0
Otu0570	Bacteria	Proteobact	Alphaprote	Caulobacte	Caulobacte	Brevundim	0
Otu0571	Bacteria	Deinococcu	Deinococci	Deinococca	Trueperace	Truepera	0
NA.56	NA	NA	NA	NA	NA	NA	0
NA.57	NA	NA	NA	NA	NA	NA	0
NA.58	NA	NA	NA	NA	NA	NA	0
NA.59	NA	NA	NA	NA	NA	NA	0
Otu0596	Bacteria	Firmicutes	Bacilli	Lactobacilla	Leuconosto	Weissella	0
NA.60	NA	NA	NA	NA	NA	NA	0
NA.61	NA	NA	NA	NA	NA	NA	0

NA.62	NA	NA	NA	NA	NA	NA	0
NA.63	NA	NA	NA	NA	NA	NA	0
Otu0626	Bacteria	Firmicutes	Bacilli	Lactobacill	Aerococcac	Aerococcus	0
Otu0638	Bacteria	Planctomy	Phycisphae	Tepidispha	WD2101_s	WD2101_s	0
NA.64	NA	NA	NA	NA	NA	NA	0
Otu0665	Bacteria	Proteobact	Alphaprote	Sphingomo	Sphingomo	Sphingomo	0
NA.65	NA	NA	NA	NA	NA	NA	0
NA.66	NA	NA	NA	NA	NA	NA	0
NA.67	NA	NA	NA	NA	NA	NA	0
NA.68	NA	NA	NA	NA	NA	NA	0
NA.69	NA	NA	NA	NA	NA	NA	0
NA.70	NA	NA	NA	NA	NA	NA	0
Otu0730	Bacteria	Actinobact	Actinobact	Frankiales	Geodermat	Geodermat	0
Otu0738	Bacteria	Firmicutes	Negativicut	Selenomon	Veillonella	Selenomon	0
Otu0742	Bacteria	Proteobact	Alphaprote	Acetobacte	Acetobacte	Craurococc	0
NA.71	NA	NA	NA	NA	NA	NA	0
NA.72	NA	NA	NA	NA	NA	NA	0
NA.73	NA	NA	NA	NA	NA	NA	0
Otu0835	Bacteria	Firmicutes	Bacilli	Bacillales	Bacillaceae	Halobacillu	0
NA.74	NA	NA	NA	NA	NA	NA	0
Otu0920	Bacteria	Acidobacte	Blastocatel	Blastocatel	Blastocatel	Blastocatel	0
Otu0072	Bacteria	Proteobact	Gammapro	Pseudomo	Pseudomo	Pseudomo	-0.00014
Otu0112	Bacteria	Firmicutes	Clostridia	Clostridiale	Lachnospir	Lachnospir	-0.00014
Otu0245	Bacteria	Firmicutes	Clostridia	Clostridiale	Ruminococ	Ruminococ	-0.00015
Otu0195	Bacteria	Firmicutes	Clostridia	Clostridiale	Clostridiale	Clostridiale	-0.00022
Otu0046	Bacteria	Firmicutes	Clostridia	Clostridiale	Lachnospir	Lachnospir	-0.00037
Otu0181	Bacteria	Firmicutes	Clostridia	Clostridiale	Ruminococ	Ruminococ	-0.00045
Otu0188	Bacteria	Proteobact	Alphaprote	Rhizobiales	Rhizobiace	Mesorhizol	-0.00058
Otu0125	Bacteria	Firmicutes	Erysipelotr	Erysipelotr	Erysipelotr	uncultured	-0.00061
Otu0108	Bacteria	Firmicutes	Clostridia	Clostridiale	Lachnospir	Lachnospir	-0.00067
NA.75	NA	NA	NA	NA	NA	NA	-0.00086
Otu0008	Bacteria	Firmicutes	Clostridia	Clostridiale	Lachnospir	uncultured	-0.0009
Otu0110	Bacteria	Firmicutes	Clostridia	Clostridiale	Lachnospir	Lachnospir	-0.001
Otu0231	Bacteria	Firmicutes	Clostridia	Clostridiale	Lachnospir	Lachnospir	-0.00121
Otu0238	Bacteria	Proteobact	Alphaprote	Rhizobiales	Xanthobact	Bradyrhizol	-0.00121
Otu0257	Bacteria	Firmicutes	Clostridia	Clostridiale	Ruminococ	Ruminiclos	-0.00121
Otu0144	Bacteria	Firmicutes	Clostridia	Clostridiale	Lachnospir	Lachnospir	-0.00124
Otu0218	Bacteria	Firmicutes	Clostridia	Clostridiale	Ruminococ	Ruminococ	-0.00126
Otu0255	Bacteria	Firmicutes	Clostridia	Clostridiale	Ruminococ	Oscillibacte	-0.00139
Otu0119	Bacteria	Firmicutes	Clostridia	Clostridiale	Ruminococ	Ruminiclos	-0.00146
Otu0201	Bacteria	Firmicutes	Clostridia	Clostridiale	Ruminococ	Anaerotrur	-0.00164
Otu0044	Bacteria	Firmicutes	Bacilli	Bacillales	Listeriacea	Listeria	-0.00186
Otu0211	Bacteria	Firmicutes	Clostridia	Clostridiale	Lachnospir	Lachnospir	-0.00199
Otu0198	Bacteria	Firmicutes	Clostridia	Clostridiale	Clostridiale	Clostridiale	-0.00205
Otu0030	Bacteria	Firmicutes	Clostridia	Clostridiale	Ruminococ	Oscillibacte	-0.00228
Otu0170	Bacteria	Firmicutes	Clostridia	Clostridiale	Lachnospir	GCA-90006	-0.00244
Otu0196	Bacteria	Firmicutes	Erysipelotr	Erysipelotr	Erysipelotr	Erysipelotr	-0.00253

Otu0200	Bacteria	Firmicutes	Clostridia	Clostridiale	Ruminococ	Ruminococ	-0.00254
Otu0130	Bacteria	Firmicutes	Clostridia	Clostridiale	Ruminococ	Oscillibacte	-0.00255
Otu0199	Bacteria	Firmicutes	Clostridia	Clostridiale	Lachnospir	Lachnospir	-0.00265
Otu0275	Bacteria	Firmicutes	Clostridia	Clostridiale	Lachnospir	Lachnospir	-0.00303
Otu0204	Bacteria	Actinobact	Actinobact	Corynebact	Nocardiace	Gordonia	-0.00353
Otu0127	Bacteria	Firmicutes	Clostridia	Clostridiale	Lachnospir	Lachnospir	-0.00358
Otu0236	Bacteria	Firmicutes	Clostridia	Clostridiale	Ruminococ	Ruminiclos	-0.00391
Otu0208	Bacteria	Firmicutes	Clostridia	Clostridiale	Lachnospir	Lachnospir	-0.00407
Otu0129	Bacteria	Firmicutes	Clostridia	Clostridiale	Ruminococ	Ruminococ	-0.00439
Otu0005	Bacteria	Bacteroide	Bacteroidia	Bacteroidal	Muribacula	Muribacula	-0.00465
Otu0230	Bacteria	Firmicutes	Clostridia	Clostridiale	Lachnospir	Lachnospir	-0.00511
Otu0106	Bacteria	Firmicutes	Clostridia	Clostridiale	Peptococca	uncultured	-0.00514
Otu0175	Bacteria	Firmicutes	Clostridia	Clostridiale	Ruminococ	Ruminococ	-0.00522
Otu0167	Bacteria	Firmicutes	Clostridia	Clostridiale	Ruminococ	Ruminiclos	-0.00527
Otu0136	Bacteria	Actinobact	Actinobact	Corynebact	Nocardiace	Rhodococc	-0.00552
Otu0164	Bacteria	Firmicutes	Clostridia	Clostridiale	Ruminococ	Ruminococ	-0.00565
Otu0226	Bacteria	Firmicutes	Clostridia	Clostridiale	Lachnospir	Lachnospir	-0.00568
Otu0192	Bacteria	Firmicutes	Clostridia	Clostridiale	Lachnospir	Lachnospir	-0.00577
Otu0187	Bacteria	Firmicutes	Clostridia	Clostridiale	Lachnospir	Acetatifact	-0.00596
Otu0067	Bacteria	Firmicutes	Clostridia	Clostridiale	Lachnospir	Lachnospir	-0.00625
Otu0124	Bacteria	Firmicutes	Clostridia	Clostridiale	Ruminococ	Ruminococ	-0.0063
Otu0177	Bacteria	Firmicutes	Clostridia	Clostridiale	Lachnospir	Lachnospir	-0.00641
Otu0063	Bacteria	Firmicutes	Clostridia	Clostridiale	Lachnospir	Lachnospir	-0.00686
Otu0094	Bacteria	Firmicutes	Clostridia	Clostridiale	Ruminococ	Intestinimc	-0.00772
NA.76	NA	NA	NA	NA	NA	NA	-0.00878
Otu0093	Bacteria	Firmicutes	Clostridia	Clostridiale	Ruminococ	Ruminiclos	-0.01014
Otu0092	Bacteria	Firmicutes	Clostridia	Clostridiale	Lachnospir	Lachnospir	-0.0107
Otu0150	Bacteria	Firmicutes	Clostridia	Clostridiale	Lachnospir	Lachnospir	-0.01135
Otu0151	Bacteria	Firmicutes	Clostridia	Clostridiale	Lachnospir	Lachnospir	-0.01144
Otu0103	Bacteria	Firmicutes	Clostridia	Clostridiale	Lachnospir	Lachnoclos	-0.01418
Otu0082	Bacteria	Firmicutes	Clostridia	Clostridiale	Lachnospir	Lachnospir	-0.01422
Otu0065	Bacteria	Firmicutes	Bacilli	Lactobacilla	Enterococc	Enterococc	-0.01427
Otu0080	Bacteria	Firmicutes	Clostridia	Clostridiale	Lachnospir	uncultured	-0.01458
Otu0176	Bacteria	Firmicutes	Clostridia	Clostridiale	Ruminococ	Ruminococ	-0.01484
Otu0084	Bacteria	Firmicutes	Clostridia	Clostridiale	Ruminococ	Ruminiclos	-0.01563
Otu0158	Bacteria	Firmicutes	Clostridia	Clostridiale	Ruminococ	Ruminococ	-0.02046
Otu0105	Bacteria	Firmicutes	Clostridia	Clostridiale	Lachnospir	Lachnospir	-0.02251
Otu0102	Bacteria	Firmicutes	Clostridia	Clostridiale	Ruminococ	Ruminococ	-0.02272
Otu0159	Bacteria	Firmicutes	Clostridia	Clostridiale	Ruminococ	Ruminococ	-0.02383
Otu0073	Bacteria	Tenericutes	Mollicutes	Anaeroplas	Anaeroplas	Anaeroplas	-0.02388
Otu0059	Bacteria	Firmicutes	Clostridia	Clostridiale	Lachnospir	GCA-90006	-0.02425
Otu0116	Bacteria	Firmicutes	Clostridia	Clostridiale	Lachnospir	Lachnospir	-0.0255
Otu0021	Bacteria	Firmicutes	Clostridia	Clostridiale	Lachnospir	Lachnospir	-0.03119
Otu0036	Bacteria	Firmicutes	Clostridia	Clostridiale	Lachnospir	Lachnospir	-0.03154
NA.77	NA	NA	NA	NA	NA	NA	-0.03348
Otu0043	Bacteria	Firmicutes	Clostridia	Clostridiale	Ruminococ	Ruminiclos	-0.03782
