# Supplementary material for: Oral Vaccination Using a Probiotic Vaccine Platform Combined with Prebiotics Impacts Immune Response and the Microbiome
Source: Vaccines (Basel). 2022 Sep 4;10(9):1465. doi: 10.3390/vaccines10091465 (PMC9504555; doi:10.3390/vaccines10091465)
Supplement: Supplementary file 1 [file vaccines-10-01465-s001.zip › Table_S6_positive_importance-fecal.pdf]

Table_S6_positive_importance-fecal

	kingdom	phylum	class	order	family	genus	pos_a_4
Otu0071	Bacteria	Firmicutes	Clostridia	Clostridiale	Ruminococ	Ruminococ	1
Otu0023	Bacteria	Firmicutes	Clostridia	Clostridiale	Lachnospir	Lachnospir	0.67293
Otu0087	Bacteria	Firmicutes	Bacilli	Lactobacilla	Streptococ	Lactococcu	0.467362
Otu0009	Bacteria	Firmicutes	Clostridia	Clostridiale	Clostridiace	Clostridium	0.436323
Otu0100	Bacteria	Firmicutes	Clostridia	Clostridiale	Lachnospir	uncultured	0.322184
Otu0089	Bacteria	Firmicutes	Clostridia	Clostridiale	Ruminococ	Anaerotrun	0.289518
Otu0053	Bacteria	Firmicutes	Clostridia	Clostridiale	Lachnospir	Lachnospir	0.268312
Otu0076	Bacteria	Firmicutes	Clostridia	Clostridiale	Lachnospir	uncultured	0.228268
Otu0048	Bacteria	Firmicutes	Clostridia	Clostridiale	Lachnospir	Acetatifact	0.2197
Otu0109	Bacteria	Firmicutes	Clostridia	Clostridiale	Lachnospir	uncultured	0.217344
Otu0064	Bacteria	Firmicutes	Erysipelotri	Erysipelotri	Erysipelotri	Turicibacte	0.187751
Otu0026	Bacteria	Firmicutes	Clostridia	Clostridiale	Lachnospir	Lachnospir	0.182919
Otu0010	Bacteria	Firmicutes	Clostridia	Clostridiale	Lachnospir	Lachnospir	0.178079
Otu0070	Bacteria	Firmicutes	Erysipelotri	Erysipelotri	Erysipelotri	Erysipelato	0.177375
Otu0017	Bacteria	Firmicutes	Bacilli	Lactobacilla	Lactobacilla	Lactobacillu	0.176532
Otu0047	Bacteria	Firmicutes	Clostridia	Clostridiale	Lachnospir	Lachnospir	0.170573
Otu0061	Bacteria	Firmicutes	Clostridia	Clostridiale	Lachnospir	Lachnospir	0.160885
Otu0051	Bacteria	Firmicutes	Clostridia	Clostridiale	Ruminococ	Ruminococ	0.135477
Otu0084	Bacteria	Firmicutes	Clostridia	Clostridiale	Ruminococ	Ruminiclos	0.13113
Otu0057	Bacteria	Firmicutes	Clostridia	Clostridiale	Lachnospir	uncultured	0.112944
Otu0111	Bacteria	Firmicutes	Clostridia	Clostridiale	Lachnospir	Lachnospir	0.105924
Otu0075	Bacteria	Firmicutes	Clostridia	Clostridiale	Lachnospir	Lachnospir	0.101105
Otu0184	Bacteria	Firmicutes	Clostridia	Clostridiale	Lachnospir	Lachnospir	0.091289
Otu0024	Bacteria	Firmicutes	Clostridia	Clostridiale	Ruminococ	Ruminococ	0.090989
Otu0096	Bacteria	Firmicutes	Clostridia	Clostridiale	Lachnospir	uncultured	0.087982
Otu0142	Bacteria	Firmicutes	Clostridia	Clostridiale	Lachnospir	Lachnospir	0.08151
Otu0068	Bacteria	Firmicutes	Clostridia	Clostridiale	Lachnospir	Lachnospir	0.081454
Otu0040	Bacteria	Firmicutes	Clostridia	Clostridiale	Lachnospir	Lachnospir	0.080927
Otu0055	Bacteria	Firmicutes	Clostridia	Clostridiale	Lachnospir	A2	0.062543
Otu0006	Bacteria	Firmicutes	Clostridia	Clostridiale	Lachnospir	Lachnospir	0.060795
Otu0013	Bacteria	Firmicutes	Clostridia	Clostridiale	Lachnospir	Lachnospir	0.057608
Otu0157	Bacteria	Firmicutes	Clostridia	Clostridiale	Ruminococ	Oscillibacte	0.056628
Otu0015	Bacteria	Firmicutes	Clostridia	Clostridiale	Lachnospir	Roseburia	0.056373
Otu0035	Bacteria	Proteobact	Gammaprc	Enterobact	Enterobact	Enterobact	0.053858
Otu0097	Bacteria	Firmicutes	Clostridia	Clostridiale	Lachnospir	GCA-90006	0.049912
Otu0139	Bacteria	Firmicutes	Clostridia	Clostridiale	Lachnospir	Lachnospir	0.048476
Otu0020	Bacteria	Firmicutes	Clostridia	Clostridiale	Lachnospir	Lachnospir	0.047895
Otu0014	Bacteria	Firmicutes	Clostridia	Clostridiale	Lachnospir	Lachnospir	0.044379
Otu0037	Bacteria	Firmicutes	Clostridia	Clostridiale	Lachnospir	Lachnospir	0.039064
Otu0125	Bacteria	Firmicutes	Erysipelotri	Erysipelotri	Erysipelotri	uncultured	0.037786
Otu0059	Bacteria	Firmicutes	Clostridia	Clostridiale	Lachnospir	GCA-90006	0.037396
Otu0042	Bacteria	Firmicutes	Clostridia	Clostridiale	Lachnospir	Lachnospir	0.037386
Otu0056	Bacteria	Firmicutes	Clostridia	Clostridiale	Lachnospir	uncultured	0.035669
Otu0105	Bacteria	Firmicutes	Clostridia	Clostridiale	Lachnospir	Lachnospir	0.035371
Otu0028	Bacteria	Firmicutes	Clostridia	Clostridiale	Lachnospir	Lachnospir	0.033542

Otu0039	Bacteria	Firmicutes	Clostridia	Clostridiale	Lachnospir	Lachnospira	0.031761
Otu0145	Bacteria	Firmicutes	Clostridia	Clostridiale	Lachnospir	Lachnospira	0.030187
Otu0038	Bacteria	Firmicutes	Clostridia	Clostridiale	Lachnospir	Lachnospira	0.028933
Otu0162	Bacteria	Firmicutes	Clostridia	Clostridiale	Lachnospir	Lachnospira	0.026729
Otu0063	Bacteria	Firmicutes	Clostridia	Clostridiale	Lachnospir	Lachnospira	0.026472
Otu0090	Bacteria	Firmicutes	Clostridia	Clostridiale	Ruminococ	Ruminococ	0.02641
Otu0083	Bacteria	Firmicutes	Clostridia	Clostridiale	Ruminococ	Ruminococ	0.025417
Otu0116	Bacteria	Firmicutes	Clostridia	Clostridiale	Lachnospir	Lachnospira	0.025203
Otu0022	Bacteria	Proteobact	Gammaprc	Enterobact	Enterobact	Escherichia	0.023676
Otu0110	Bacteria	Firmicutes	Clostridia	Clostridiale	Lachnospir	Lachnospira	0.023516
Otu0058	Bacteria	Firmicutes	Clostridia	Clostridiale	Lachnospir	uncultured	0.023402
Otu0081	Bacteria	Firmicutes	Clostridia	Clostridiale	Lachnospir	Lachnoclos	0.022538
Otu0016	Bacteria	Firmicutes	Clostridia	Clostridiale	Lachnospir	Lachnospira	0.022307
Otu0092	Bacteria	Firmicutes	Clostridia	Clostridiale	Lachnospir	Lachnospira	0.021656
Otu0117	Bacteria	Firmicutes	Clostridia	Clostridiale	Ruminococ	Ruminiclos	0.021538
Otu0036	Bacteria	Firmicutes	Clostridia	Clostridiale	Lachnospir	Lachnospira	0.020795
Otu0132	Bacteria	Actinobact	Actinobact	Micrococca	Micrococca	Micrococca	0.02076
Otu0101	Bacteria	Firmicutes	Clostridia	Clostridiale	Lachnospir	ASF356	0.020525
Otu0074	Bacteria	Firmicutes	Clostridia	Clostridiale	Lachnospir	Lachnospira	0.020472
Otu0149	Bacteria	Firmicutes	Clostridia	Clostridiale	Ruminococ	Ruminococ	0.020008
Otu0073	Bacteria	Tenericutes	Mollicutes	Anaeroplas	Anaeroplas	Anaeroplas	0.019977
Otu0112	Bacteria	Firmicutes	Clostridia	Clostridiale	Lachnospir	Lachnospira	0.019632
Otu0128	Bacteria	Firmicutes	Clostridia	Clostridiale	Family_XIII	Family_XIII	0.01953
Otu0119	Bacteria	Firmicutes	Clostridia	Clostridiale	Ruminococ	Ruminiclos	0.018497
Otu0050	Bacteria	Firmicutes	Clostridia	Clostridiale	Ruminococ	Oscillibacte	0.018439
Otu0088	Bacteria	Firmicutes	Clostridia	Clostridiale	Ruminococ	Butyricicoc	0.018246
Otu0153	Bacteria	Firmicutes	Clostridia	Clostridiale	Ruminococ	Ruminococ	0.017896
Otu0174	Bacteria	Firmicutes	Clostridia	Clostridiale	Lachnospir	Lachnospira	0.017813
Otu0135	Bacteria	Firmicutes	Clostridia	Clostridiale	Lachnospir	Lachnospira	0.01753
Otu0032	Bacteria	Firmicutes	Clostridia	Clostridiale	Ruminococ	Ruminiclos	0.016388
Otu0130	Bacteria	Firmicutes	Clostridia	Clostridiale	Ruminococ	Oscillibacte	0.015375
Otu0243	Bacteria	Firmicutes	Clostridia	Clostridiale	Lachnospir	Lachnospira	0.014265
Otu0159	Bacteria	Firmicutes	Clostridia	Clostridiale	Ruminococ	Ruminococ	0.014246
Otu0018	Bacteria	Firmicutes	Clostridia	Clostridiale	Ruminococ	Ruminococ	0.014062
Otu0046	Bacteria	Firmicutes	Clostridia	Clostridiale	Lachnospir	Lachnospira	0.012762
Otu0069	Bacteria	Firmicutes	Clostridia	Clostridiale	Lachnospir	uncultured	0.011927
Otu0190	Bacteria	Firmicutes	Clostridia	Clostridiale	Lachnospir	Lachnospira	0.011772
Otu0060	Bacteria	Firmicutes	Clostridia	Clostridiale	Peptostrep	Romboutsi	0.011691
Otu0134	Bacteria	Firmicutes	Clostridia	Clostridiale	Lachnospir	Lachnospira	0.011353
Otu0175	Bacteria	Firmicutes	Clostridia	Clostridiale	Ruminococ	Ruminococ	0.011061
Otu0144	Bacteria	Firmicutes	Clostridia	Clostridiale	Lachnospir	Lachnospira	0.010898
Otu0183	Bacteria	Firmicutes	Clostridia	Clostridiale	Ruminococ	Ruminiclos	0.010134
Otu0201	Bacteria	Firmicutes	Clostridia	Clostridiale	Ruminococ	Anaerotrur	0.010066
Otu0065	Bacteria	Firmicutes	Bacilli	Lactobacilla	Enterococc	Enterococc	0.009992
Otu0129	Bacteria	Firmicutes	Clostridia	Clostridiale	Ruminococ	Ruminococ	0.009315
Otu0045	Bacteria	Firmicutes	Bacilli	Bacillales	Staphyloco	Staphyloco	0.009112
Otu0182	Bacteria	Firmicutes	Clostridia	Clostridiale	Lachnospir	Lachnospira	0.009076

Otu0099	Bacteria	Actinobact	Coriobacte	Coriobacte	Eggerthella	Adlercreutz	0.008542
Otu0077	Bacteria	Firmicutes	Clostridia	Clostridiale	Ruminococ	Ruminiclos	0.008197
Otu0141	Bacteria	Actinobact	Coriobacte	Coriobacte	Eggerthella	Eggerthella	0.008035
Otu0093	Bacteria	Firmicutes	Clostridia	Clostridiale	Ruminococ	Ruminiclos	0.007376
Otu0126	Bacteria	Firmicutes	Clostridia	Clostridiale	Lachnospir	Lachnospir	0.0073
Otu0106	Bacteria	Firmicutes	Clostridia	Clostridiale	Peptococca	uncultured	0.007274
Otu0027	Bacteria	Firmicutes	Clostridia	Clostridiale	Lachnospir	Acetatifact	0.007117
Otu0168	Bacteria	Firmicutes	Clostridia	Clostridiale	Clostridiale	Clostridiale	0.00703
Otu0131	Bacteria	Proteobact	Alphaprote	Caulobacte	Caulobacte	Brevundim	0.006729
Otu0103	Bacteria	Firmicutes	Clostridia	Clostridiale	Lachnospir	Lachnoclos	0.006605
Otu0167	Bacteria	Firmicutes	Clostridia	Clostridiale	Ruminococ	Ruminiclos	0.006599
Otu0080	Bacteria	Firmicutes	Clostridia	Clostridiale	Lachnospir	uncultured	0.006323
Otu0191	Bacteria	Firmicutes	Clostridia	Clostridiale	Clostridiale	Clostridiale	0.006183
Otu0008	Bacteria	Firmicutes	Clostridia	Clostridiale	Lachnospir	uncultured	0.005948
Otu0012	Bacteria	Firmicutes	Clostridia	Clostridiale	Lachnospir	Lachnospir	0.005945
Otu0062	Bacteria	Proteobact	Alphaprote	Rhizobiales	Rhizobiace	Rhizobiace	0.005922
Otu0161	Bacteria	Firmicutes	Clostridia	Clostridiale	Clostridiale	Clostridiale	0.005904
Otu0001	Bacteria	Bacteroide	Bacteroidia	Bacteroidal	Muribacula	Muribacula	0.005817
Otu0085	Bacteria	Firmicutes	Clostridia	Clostridiale	Lachnospir	Lachnospir	0.005722
Otu0170	Bacteria	Firmicutes	Clostridia	Clostridiale	Lachnospir	GCA-90006	0.005454
Otu0034	Bacteria	Firmicutes	Clostridia	Clostridiale	Lachnospir	Acetatifact	0.005432
Otu0003	Bacteria	Firmicutes	Clostridia	Clostridiale	Lachnospir	Lachnospir	0.005409
NA.1	NA	NA	NA	NA	NA	NA	0.004865
Otu0151	Bacteria	Firmicutes	Clostridia	Clostridiale	Lachnospir	Lachnospir	0.004726
Otu0177	Bacteria	Firmicutes	Clostridia	Clostridiale	Lachnospir	Lachnospir	0.004692
Otu0136	Bacteria	Actinobact	Actinobact	Corynebact	Nocardiace	Rhodococc	0.004663
Otu0021	Bacteria	Firmicutes	Clostridia	Clostridiale	Lachnospir	Lachnospir	0.004607
Otu0160	Bacteria	Firmicutes	Clostridia	Clostridiale	Ruminococ	Ruminococ	0.004508
Otu0205	Bacteria	Firmicutes	Clostridia	Clostridiale	Ruminococ	Ruminococ	0.004508
Otu0207	Bacteria	Firmicutes	Clostridia	Clostridiale	Lachnospir	Lachnospir	0.004285
Otu0152	Bacteria	Tenericutes	Mollicutes	Mollicutes_	Mollicutes_	Mollicutes_	0.004181
Otu0044	Bacteria	Firmicutes	Bacilli	Bacillales	Listeriaceae	Listeria	0.003851
Otu0140	Bacteria	Firmicutes	Clostridia	Clostridiale	Lachnospir	Lachnospir	0.003731
Otu0163	Bacteria	Firmicutes	Clostridia	Clostridiale	Lachnospir	Lachnospir	0.003685
Otu0002	Bacteria	Bacteroide	Bacteroidia	Bacteroidal	Muribacula	Muribacula	0.003667
Otu0150	Bacteria	Firmicutes	Clostridia	Clostridiale	Lachnospir	Lachnospir	0.003638
Otu0072	Bacteria	Proteobact	Gammapro	Pseudomo	Pseudomo	Pseudomo	0.003618
Otu0193	Bacteria	Firmicutes	Clostridia	Clostridiale	Ruminococ	Ruminiclos	0.003367
Otu0054	Bacteria	Firmicutes	Clostridia	Clostridiale	Lachnospir	Lachnospir	0.003048
Otu0166	Bacteria	Firmicutes	Clostridia	Clostridiale	Ruminococ	Oscillibacte	0.002816
Otu0214	Bacteria	Firmicutes	Clostridia	Clostridiale	Lachnospir	Lachnospir	0.002811
Otu0232	Bacteria	Firmicutes	Clostridia	Clostridiale	Ruminococ	Ruminococ	0.002792
Otu0079	Bacteria	Firmicutes	Clostridia	Clostridiale	Lachnospir	Lachnospir	0.002661
Otu0187	Bacteria	Firmicutes	Clostridia	Clostridiale	Lachnospir	Acetatifact	0.002626
Otu0198	Bacteria	Firmicutes	Clostridia	Clostridiale	Clostridiale	Clostridiale	0.002454
Otu0121	Bacteria	Firmicutes	Clostridia	Clostridiale	Lachnospir	Lachnospir	0.002145
Otu0231	Bacteria	Firmicutes	Clostridia	Clostridiale	Lachnospir	Lachnospir	0.00199

Otu0192	Bacteria	Firmicutes	Clostridia	Clostridiale	Lachnospir.	Lachnospir.	0.001975
Otu0172	Bacteria	Firmicutes	Clostridia	Clostridiale	Lachnospir.	Lachnospir.	0.001875
Otu0218	Bacteria	Firmicutes	Clostridia	Clostridiale	Ruminococ	Ruminococ	0.001769
Otu0242	Bacteria	Proteobact	Gammapro	Betaprotec	Burkholder	Ralstonia	0.001745
Otu0228	Bacteria	Tenericutes	Mollicutes	Mollicutes_	Mollicutes_	Mollicutes_	0.00156
Otu0229	Bacteria	Firmicutes	Clostridia	Clostridiale	Lachnospir.	Lachnospir.	0.00156
Otu0292	Bacteria	Firmicutes	Clostridia	Clostridiale	Lachnospir.	Lachnospir.	0.001396
Otu0230	Bacteria	Firmicutes	Clostridia	Clostridiale	Lachnospir.	Lachnospir.	0.001263
Otu0238	Bacteria	Proteobact	Alphaprote	Rhizobiales	Xanthobact	Bradyrhizol	0.001263
Otu0221	Bacteria	Firmicutes	Clostridia	Clostridiale	Ruminococ	GCA-90006	0.001206
Otu0235	Bacteria	Firmicutes	Clostridia	Clostridiale	Clostridiale	Clostridiale	0.001153
Otu0133	Bacteria	Firmicutes	Clostridia	Clostridiale	Ruminococ	Ruminococ	0.000697
Otu0114	Bacteria	Firmicutes	Clostridia	Clostridiale	Lachnospir.	Lachnospir.	0.000647
Otu0146	Bacteria	Firmicutes	Clostridia	Clostridiale	Lachnospir.	Lachnospir.	0.000541
Otu0138	Bacteria	Firmicutes	Clostridia	Clostridiale	Ruminococ	Ruminococ	0.000477
Otu0154	Bacteria	Firmicutes	Clostridia	Clostridiale	Lachnospir.	Lachnospir.	0.000302
Otu0169	Bacteria	Firmicutes	Erysipelotri	Erysipelotri	Erysipelotri	Erysipelotri	0.000234
Otu0223	Bacteria	Firmicutes	Clostridia	Clostridiale	Lachnospir.	Lachnospir.	8.67E-05
Otu0113	Bacteria	Firmicutes	Bacilli	Lactobacilla	Lactobacilla	Lactobacillu	7.76E-05
Otu0156	Bacteria	Actinobact	Actinobact	Micrococca	Microbacte	Curtobacte	0
Otu0165	Bacteria	Firmicutes	Bacilli	Bacillales	Bacillaceae	Bacillus	0
Otu0178	Bacteria	Firmicutes	Clostridia	Clostridiale	Lachnospir.	Lachnospir.	0
Otu0185	Bacteria	Firmicutes	Clostridia	Clostridiale	Ruminococ	Ruminococ	0
Otu0186	Bacteria	Firmicutes	Clostridia	Clostridiale	Lachnospir.	Lachnospir.	0
Otu0202	Bacteria	Firmicutes	Clostridia	Clostridiale	Clostridiale	Clostridiale	0
Otu0204	Bacteria	Actinobact	Actinobact	Corynebact	Nocardiace	Gordonia	0
Otu0212	Bacteria	Firmicutes	Clostridia	Clostridiale	Ruminococ	Ruminococ	0
Otu0215	Bacteria	Tenericutes	Mollicutes	Mollicutes_	Mollicutes_	Mollicutes_	0
Otu0219	Bacteria	Verrucomic	Verrucomic	Verrucomic	Akkermans	Akkermans	0
Otu0222	Bacteria	Firmicutes	Erysipelotri	Erysipelotri	Erysipelotri	Candidatus	0
Otu0226	Bacteria	Firmicutes	Clostridia	Clostridiale	Lachnospir.	Lachnospir.	0
Otu0227	Bacteria	Firmicutes	Clostridia	Clostridiale	Ruminococ	Ruminococ	0
Otu0234	Bacteria	Bacteroide	Bacteroidia	Bacteroidal	Muribacula	Muribacula	0
Otu0236	Bacteria	Firmicutes	Clostridia	Clostridiale	Ruminococ	Ruminiclos	0
Otu0237	Bacteria	Firmicutes	Clostridia	Clostridiale	Lachnospir.	Lachnospir.	0
Otu0239	Bacteria	Firmicutes	Clostridia	Clostridiale	Ruminococ	Ruminococ	0
Otu0240	Bacteria	Firmicutes	Clostridia	Clostridiale	Lachnospir.	Lachnospir.	0
Otu0244	Bacteria	Firmicutes	Clostridia	Clostridiale	Clostridiale	Clostridiale	0
Otu0245	Bacteria	Firmicutes	Clostridia	Clostridiale	Ruminococ	Ruminococ	0
Otu0246	Bacteria	Firmicutes	Clostridia	Clostridiale	Family_XIII	Family_XIII_	0
Otu0247	Bacteria	Firmicutes	Clostridia	Clostridiale	Clostridiale	Clostridiale	0
Otu0248	Bacteria	Firmicutes	Clostridia	Clostridiale	Ruminococ	Ruminococ	0
Otu0249	Bacteria	Firmicutes	Clostridia	Clostridiale	Ruminococ	Ruminococ	0
Otu0250	Bacteria	Firmicutes	Clostridia	Clostridiale	Lachnospir.	Lachnospir.	0
Otu0251	Bacteria	Firmicutes	Clostridia	Clostridiale	Ruminococ	Ruminiclos	0
Otu0252	Bacteria	Firmicutes	Clostridia	Clostridiale	Lachnospir.	Lachnospir.	0
Otu0253	Bacteria	Firmicutes	Clostridia	Clostridiale	Clostridiale	Clostridiale	0

Otu0254	Bacteria	Firmicutes	Clostridia	Clostridiale	Lachnospir	Lachnospir		0
Otu0255	Bacteria	Firmicutes	Clostridia	Clostridiale	Ruminococ	Oscillibacte		0
Otu0256	Bacteria	Firmicutes	Clostridia	Clostridiale	Lachnospir	Lachnospir		0
Otu0259	Bacteria	Tenericute	Mollicutes	Mollicutes_	Mollicutes_	Mollicutes_		0
Otu0260	Bacteria	Firmicutes	Firmicutes_	Firmicutes_	Firmicutes_	Firmicutes_		0
Otu0261	Bacteria	Firmicutes	Clostridia	Clostridiale	Lachnospir	Lachnospir		0
Otu0262	Bacteria	Firmicutes	Clostridia	Clostridiale	Ruminococ	Ruminiclos		0
Otu0263	Bacteria	Firmicutes	Clostridia	Clostridiale	Family_XIII	Family_XIII_		0
Otu0264	Bacteria	Firmicutes	Clostridia	Clostridiale	Clostridiale	Clostridiale		0
Otu0266	Bacteria	Firmicutes	Clostridia	Clostridiale	Ruminococ	Ruminococ		0
Otu0267	Bacteria	Firmicutes	Bacilli	Lactobacill	Lactobacill	Lactobacill		0
Otu0269	Bacteria	Bacteroide	Bacteroidia	Bacteroidal	Muribacula	Muribacula		0
Otu0270	Bacteria	Firmicutes	Clostridia	Clostridiale	Lachnospir	Lachnospir		0
Otu0271	Bacteria	Firmicutes	Clostridia	Clostridiale	Clostridiale	Clostridiale		0
Otu0272	Bacteria	Firmicutes	Clostridia	Clostridiale	Ruminococ	Ruminococ		0
Otu0273	Bacteria	Firmicutes	Clostridia	Clostridiale	Clostridiale	Clostridiale		0
Otu0274	Bacteria	Firmicutes	Clostridia	Clostridiale	Clostridiale	Clostridiale		0
Otu0275	Bacteria	Firmicutes	Clostridia	Clostridiale	Lachnospir	Lachnospir		0
Otu0276	Bacteria	Firmicutes	Clostridia	Clostridiale	Ruminococ	Ruminococ		0
Otu0277	Bacteria	Firmicutes	Clostridia	Clostridiale	Lachnospir	Lachnospir		0
Otu0278	Bacteria	Actinobact	Coriobacte	Coriobacte	Eggerthella	Eggerthella		0
Otu0279	Bacteria	Bacteroide	Bacteroidia	Bacteroidal	Muribacula	Muribacula		0
Otu0280	Bacteria	Firmicutes	Clostridia	Clostridiale	Lachnospir	Lachnospir		0
Otu0282	Bacteria	Actinobact	Actinobact	Micrococca	Brevibacte	Brevibacte		0
Otu0283	Bacteria	Bacteroide	Bacteroidia	Bacteroidal	Muribacula	Muribacula		0
Otu0284	Bacteria	Firmicutes	Clostridia	Clostridiale	Ruminococ	Ruminococ		0
Otu0285	Bacteria	Proteobact	Alphaprote	Rhizobiales	Beijerinckia	Methyloba		0
Otu0286	Bacteria	Firmicutes	Clostridia	Clostridiale	Ruminococ	Ruminococ		0
Otu0287	Bacteria	Firmicutes	Clostridia	Clostridiale	Ruminococ	Butyricicoc		0
Otu0288	Bacteria	Firmicutes	Clostridia	Clostridiale	Lachnospir	Lachnospir		0
NA.2	NA	NA	NA	NA	NA	NA		0
Otu0290	Bacteria	Firmicutes	Clostridia	Clostridiale	Clostridiale	Clostridiale		0
Otu0291	Bacteria	Proteobact	Alphaprote	Rhizobiales	Beijerinckia	Methyloba		0
Otu0293	Bacteria	Tenericute	Mollicutes	Mollicutes_	Mollicutes_	Mollicutes_		0
Otu0294	Bacteria	Firmicutes	Clostridia	Clostridiale	Ruminococ	Ruminococ		0
Otu0295	Bacteria	Firmicutes	Clostridia	Clostridiale	Lachnospir	Lachnoclos		0
Otu0296	Bacteria	Firmicutes	Clostridia	Clostridiale	Ruminococ	Ruminococ		0
Otu0297	Bacteria	Proteobact	Gammaprc	Pseudomo	Pseudomo	Pseudomo		0
Otu0298	Bacteria	Firmicutes	Clostridia	Clostridiale	Lachnospir	Lachnospir		0
NA.3	NA	NA	NA	NA	NA	NA		0
Otu0301	Bacteria	Firmicutes	Clostridia	Clostridiale	Lachnospir	Lachnospir		0
NA.4	NA	NA	NA	NA	NA	NA		0
Otu0303	Bacteria	Firmicutes	Clostridia	Clostridiale	Lachnospir	Lachnospir		0
Otu0304	Bacteria	Firmicutes	Clostridia	Clostridiale	Ruminococ	Flavonifrac		0
NA.5	NA	NA	NA	NA	NA	NA		0
Otu0306	Bacteria	Firmicutes	Clostridia	Clostridiale	Lachnospir	Lachnospir		0
Otu0307	Bacteria	Firmicutes	Bacilli	Bacillales	Paenibacill	Paenibacill		0

Otu0310	Bacteria	Proteobact	Alphaprote	Rhizobiales	Labraceae	Labrys	0
Otu0312	Bacteria	Firmicutes	Clostridia	Clostridiale	Ruminococ	Ruminiclos	0
Otu0313	Bacteria	Firmicutes	Clostridia	Clostridiale	Lachnospir	Lachnoclos	0
Otu0317	Bacteria	Firmicutes	Clostridia	Clostridiale	Lachnospir	Lachnospir	0
Otu0318	Bacteria	Firmicutes	Clostridia	Clostridiale	Ruminococ	Ruminococ	0
NA.6	NA	NA	NA	NA	NA	NA	0
Otu0322	Bacteria	Firmicutes	Clostridia	Clostridiale	Lachnospir	Lachnospir	0
Otu0323	Bacteria	Firmicutes	Clostridia	Clostridiale	Lachnospir	Lachnospir	0
NA.7	NA	NA	NA	NA	NA	NA	0
NA.8	NA	NA	NA	NA	NA	NA	0
Otu0330	Bacteria	Proteobact	Alphaprote	Rhizobiales	Rhizobiace	Phyllobacte	0
NA.9	NA	NA	NA	NA	NA	NA	0
Otu0333	Bacteria	Firmicutes	Clostridia	Clostridiale	Lachnospir	Lachnospir	0
NA.10	NA	NA	NA	NA	NA	NA	0
Otu0335	Bacteria	Firmicutes	Clostridia	Clostridiale	Ruminococ	Ruminococ	0
NA.11	NA	NA	NA	NA	NA	NA	0
NA.12	NA	NA	NA	NA	NA	NA	0
Otu0339	Bacteria	Firmicutes	Clostridia	Clostridiale	Lachnospir	Acetatifact	0
Otu0342	Bacteria	Firmicutes	Clostridia	Clostridiale	Clostridiale	Clostridiale	0
Otu0343	Bacteria	Firmicutes	Clostridia	Clostridiale	Lachnospir	Lachnospir	0
NA.13	NA	NA	NA	NA	NA	NA	0
NA.14	NA	NA	NA	NA	NA	NA	0
Otu0348	Bacteria	Proteobact	Gammapro	Gammapro	Unknown_	Acidibacter	0
Otu0351	Bacteria	Firmicutes	Clostridia	Clostridiale	Clostridiale	Clostridiale	0
Otu0352	Bacteria	Bacteroide	Bacteroidia	Bacteroidal	Muribacula	Muribacula	0
Otu0353	Bacteria	Firmicutes	Clostridia	Clostridiale	Ruminococ	Ruminiclos	0
Otu0356	Bacteria	Firmicutes	Clostridia	Clostridiale	Lachnospir	Lachnospir	0
NA.15	NA	NA	NA	NA	NA	NA	0
Otu0361	Bacteria	Tenericutes	Mollicutes	Mollicutes_	Mollicutes_	Mollicutes_	0
Otu0366	Bacteria	Firmicutes	Clostridia	Clostridiale	Lachnospir	Lachnospir	0
NA.16	NA	NA	NA	NA	NA	NA	0
Otu0370	Bacteria	Tenericutes	Mollicutes	Mollicutes_	Mollicutes_	Mollicutes_	0
NA.17	NA	NA	NA	NA	NA	NA	0
NA.18	NA	NA	NA	NA	NA	NA	0
NA.19	NA	NA	NA	NA	NA	NA	0
NA.20	NA	NA	NA	NA	NA	NA	0
Otu0382	Bacteria	Proteobact	Alphaprote	Rhizobiales	Xanthobact	Rhodopseu	0
NA.21	NA	NA	NA	NA	NA	NA	0
Otu0386	Bacteria	Tenericutes	Mollicutes	Mollicutes_	Mollicutes_	Mollicutes_	0
Otu0387	Bacteria	Proteobact	Gammapro	Betaprotec	Burkholder	Janthinoba	0
Otu0390	Bacteria	Bacteroide	Bacteroidia	Bacteroidal	Prevotellac	Prevotella_	0
NA.22	NA	NA	NA	NA	NA	NA	0
NA.23	NA	NA	NA	NA	NA	NA	0
Otu0401	Bacteria	Firmicutes	Clostridia	Clostridiale	Lachnospir	Lachnospir	0
NA.24	NA	NA	NA	NA	NA	NA	0
NA.25	NA	NA	NA	NA	NA	NA	0
NA.26	NA	NA	NA	NA	NA	NA	0

Otu0412	Bacteria	Firmicutes	Clostridia	Clostridiale	Lachnospir	Lachnospir	0
NA.27	NA	NA	NA	NA	NA	NA	0
Otu0425	Bacteria	Proteobact	Gammapro	Enterobact	Enterobact	Yersinia	0
NA.28	NA	NA	NA	NA	NA	NA	0
Otu0428	Bacteria	Proteobact	Alphaprote	Sphingomo	Sphingomo	Sphingomo	0
NA.29	NA	NA	NA	NA	NA	NA	0
Otu0434	Bacteria	Proteobact	Gammapro	Betaprotec	Burkholder	Pelomonas	0
Otu0435	Bacteria	Firmicutes	Clostridia	Clostridiale	Family_XI	Anaerococ	0
NA.30	NA	NA	NA	NA	NA	NA	0
NA.31	NA	NA	NA	NA	NA	NA	0
Otu0444	Bacteria	Cyanobact	Melainabac	Obscuribac	Obscuribac	Obscuribac	0
Otu0447	Bacteria	Tenericute	Mollicutes	Mollicutes_	Mollicutes_	Mollicutes_	0
Otu0448	Bacteria	Firmicutes	Clostridia	Clostridiale	Ruminococ	Faecalibact	0
Otu0451	Bacteria	Acidobacte	Acidobacte	Subgroup_	Subgroup_	Subgroup_	0
NA.32	NA	NA	NA	NA	NA	NA	0
Otu0454	Bacteria	Actinobact	Actinobact	Corynebact	Corynebact	Corynebact	0
NA.33	NA	NA	NA	NA	NA	NA	0
NA.34	NA	NA	NA	NA	NA	NA	0
Otu0460	Bacteria	Actinobact	Actinobact	Micrococca	Micrococca	Kocuria	0
Otu0464	Bacteria	Proteobact	Alphaprote	Rhizobiales	Rhizobiace	Aureimona	0
NA.35	NA	NA	NA	NA	NA	NA	0
NA.36	NA	NA	NA	NA	NA	NA	0
Otu0476	Bacteria	Bacteroide	Bacteroidia	Sphingobac	Sphingobac	Sphingobac	0
Otu0484	Bacteria	Firmicutes	Bacilli	Bacillales	Bacillales_	Bacillales_	0
Otu0485	Bacteria	Firmicutes	Clostridia	Clostridiale	Ruminococ	Caproicipro	0
Otu0492	Bacteria	Proteobact	Gammapro	Pseudomon	Moraxellac	Enhydroba	0
NA.37	NA	NA	NA	NA	NA	NA	0
NA.38	NA	NA	NA	NA	NA	NA	0
Otu0501	Bacteria	Proteobact	Alphaprote	Sphingomo	Sphingomo	Sphingomo	0
NA.39	NA	NA	NA	NA	NA	NA	0
NA.40	NA	NA	NA	NA	NA	NA	0
NA.41	NA	NA	NA	NA	NA	NA	0
NA.42	NA	NA	NA	NA	NA	NA	0
Otu0534	Bacteria	Deinococcu	Deinococci	Thermales	Thermacea	Thermus	0
Otu0540	Bacteria	Proteobact	Alphaprote	Caulobacte	Caulobacte	uncultured	0
Otu0541	Bacteria	Firmicutes	Negativicut	Selenomon	Veillonellac	Veillonella	0
NA.43	NA	NA	NA	NA	NA	NA	0
Otu0551	Bacteria	Bacteroide	Bacteroidia	Bacteroidal	Prevotellac	Prevotella_	0
NA.44	NA	NA	NA	NA	NA	NA	0
NA.45	NA	NA	NA	NA	NA	NA	0
NA.46	NA	NA	NA	NA	NA	NA	0
NA.47	NA	NA	NA	NA	NA	NA	0
NA.48	NA	NA	NA	NA	NA	NA	0
Otu0578	Bacteria	Proteobact	Gammapro	Betaprotec	Burkholder	Achromoba	0
Otu0588	Bacteria	Planctomy	Planctomy	Planctomy	Rubinispha	uncultured	0
Otu0593	Bacteria	Planctomy	Planctomy	Planctomy	Rubinispha	Planctomic	0
Otu0595	Bacteria	Actinobact	Actinobact	Corynebact	Corynebact	Corynebact	0

NA.49	NA	NA	NA	NA	NA	NA	0
Otu0606	Bacteria	Proteobact	Gammaprc	Betaprotec	Burkholder	Burkholder	0
NA.50	NA	NA	NA	NA	NA	NA	0
Otu0608	Bacteria	Actinobact	Actinobact	Actinobact	Actinobact	Actinobact	0
Otu0611	Bacteria	Firmicutes	Bacilli	Bacillales	Bacillales_u	Bacillales_u	0
NA.51	NA	NA	NA	NA	NA	NA	0
NA.52	NA	NA	NA	NA	NA	NA	0
Otu0659	Bacteria	Firmicutes	Clostridia	Clostridiale	Ruminococ	Ruminococ	0
NA.53	NA	NA	NA	NA	NA	NA	0
Otu0667	Bacteria	Firmicutes	Bacilli	Bacillales	Bacillaceae	Oceanobac	0
Otu0677	Bacteria	Actinobact	Actinobact	Propioniba	Nocardioid	Nocardioid	0
Otu0683	Bacteria	Cyanobact	Melainabac	Obscuribac	Obscuribac	Obscuribac	0
Otu0718	Bacteria	Planctomy	Planctomy	Pirellulales	Pirellulacea	Pirellulacea	0
Otu0722	Bacteria	Proteobact	Gammaprc	Steroidoba	Steroidoba	uncultured	0
Otu0727	Bacteria	Firmicutes	Clostridia	Clostridiale	Clostridiace	Clostridiace	0
NA.54	NA	NA	NA	NA	NA	NA	0
NA.55	NA	NA	NA	NA	NA	NA	0
NA.56	NA	NA	NA	NA	NA	NA	0
NA.57	NA	NA	NA	NA	NA	NA	0
Otu0764	Bacteria	Firmicutes	Bacilli	Lactobacilla	Lactobacilla	Lactobacillu	0
Otu0781	Bacteria	Deinococcu	Deinococci	Deinococca	Deinococca	Deinococcu	0
NA.58	NA	NA	NA	NA	NA	NA	0
Otu0865	Bacteria	Actinobact	Actinobact	Micrococca	Intrasporar	Ornithinim	0
NA.59	NA	NA	NA	NA	NA	NA	0
Otu0123	Bacteria	Firmicutes	Clostridia	Clostridiale	Lachnospira	Lachnospira	-0.00022
Otu0104	Bacteria	Firmicutes	Clostridia	Clostridiale	Lachnospira	Tyzzerella	-0.00031
Otu0220	Bacteria	Firmicutes	Clostridia	Clostridiale	Lachnospira	Lachnospira	-0.00035
Otu0173	Bacteria	Firmicutes	Clostridia	Clostridiale	Ruminococ	Ruminococ	-0.00037
Otu0233	Bacteria	Firmicutes	Clostridia	Clostridiale	Lachnospira	Lachnospira	-0.00039
Otu0195	Bacteria	Firmicutes	Clostridia	Clostridiale	Clostridiale	Clostridiale	-0.00041
Otu0107	Bacteria	Proteobact	Alphaprote	Rhizobiales	Rhizobiacea	Brucella	-0.00072
Otu0095	Bacteria	Firmicutes	Clostridia	Clostridiale	Lachnospira	Lachnospira	-0.00119
Otu0181	Bacteria	Firmicutes	Clostridia	Clostridiale	Ruminococ	Ruminococ	-0.00127
Otu0194	Bacteria	Firmicutes	Clostridia	Clostridiale	Lachnospira	Lachnospira	-0.00133
Otu0224	Bacteria	Firmicutes	Clostridia	Clostridiale	Lachnospira	Lachnospira	-0.00133
Otu0209	Bacteria	Firmicutes	Clostridia	Clostridiale	Peptococca	uncultured	-0.0014
Otu0265	Bacteria	Firmicutes	Clostridia	Clostridiale	Lachnospira	Tyzzerella_	-0.0014
Otu0143	Bacteria	Proteobact	Gammaprc	Xanthomor	Xanthomor	Stenotroph	-0.00151
Otu0199	Bacteria	Firmicutes	Clostridia	Clostridiale	Lachnospira	Lachnospira	-0.00156
Otu0211	Bacteria	Firmicutes	Clostridia	Clostridiale	Lachnospira	Lachnospira	-0.00166
Otu0179	Bacteria	Firmicutes	Clostridia	Clostridiale	Lachnospira	Lachnospira	-0.00167
Otu0213	Bacteria	Firmicutes	Clostridia	Clostridiale	Clostridiale	Clostridiale	-0.00177
Otu0137	Bacteria	Firmicutes	Clostridia	Clostridiale	Lachnospira	Lachnospira	-0.0019
Otu0019	Bacteria	Firmicutes	Clostridia	Clostridiale	Ruminococ	Ruminiclos	-0.00195
Otu0031	Bacteria	Firmicutes	Clostridia	Clostridiale	Lachnospira	Marvinbrya	-0.00202
Otu0189	Bacteria	Firmicutes	Clostridia	Clostridiale	Lachnospira	Lachnospira	-0.00226
Otu0171	Bacteria	Firmicutes	Clostridia	Clostridiale	Ruminococ	Ruminococ	-0.00241

Otu0147	Bacteria	Firmicutes	Clostridia	Clostridiale	Lachnospira	Lachnospira	-0.00241
Otu0257	Bacteria	Firmicutes	Clostridia	Clostridiale	Ruminococ	Ruminiclos	-0.00244
Otu0360	Bacteria	Proteobact	Gammaprc	Pseudomor	Pseudomor	Pseudomor	-0.00255
Otu0158	Bacteria	Firmicutes	Clostridia	Clostridiale	Ruminococ	Ruminococ	-0.00325
Otu0155	Bacteria	Firmicutes	Clostridia	Clostridiale	Ruminococ	Anaerotrur	-0.00373
Otu0098	Bacteria	Firmicutes	Clostridia	Clostridiale	Ruminococ	Ruminococ	-0.00413
Otu0188	Bacteria	Proteobact	Alphaprote	Rhizobiales	Rhizobiacea	Mesorhizol	-0.00429
Otu0164	Bacteria	Firmicutes	Clostridia	Clostridiale	Ruminococ	Ruminococ	-0.00444
Otu0206	Bacteria	Firmicutes	Clostridia	Clostridiale	Ruminococ	Ruminococ	-0.00448
Otu0176	Bacteria	Firmicutes	Clostridia	Clostridiale	Ruminococ	Ruminococ	-0.00475
Otu0005	Bacteria	Bacteroide	Bacteroidia	Bacteroidal	Muribacula	Muribacula	-0.00489
Otu0108	Bacteria	Firmicutes	Clostridia	Clostridiale	Lachnospira	Lachnospira	-0.00498
Otu0216	Bacteria	Firmicutes	Clostridia	Clostridiale	Lachnospira	Lachnospira	-0.00518
Otu0148	Bacteria	Firmicutes	Clostridia	Clostridiale	Lachnospira	Lachnospira	-0.00519
Otu0122	Bacteria	Firmicutes	Clostridia	Clostridiale	Ruminococ	Ruminiclos	-0.00532
Otu0115	Bacteria	Firmicutes	Clostridia	Clostridiale	Lachnospira	Tyzzerella_	-0.00571
Otu0200	Bacteria	Firmicutes	Clostridia	Clostridiale	Ruminococ	Ruminococ	-0.00588
Otu0066	Bacteria	Firmicutes	Clostridia	Clostridiale	Lachnospira	Lachnospira	-0.00602
Otu0180	Bacteria	Firmicutes	Clostridia	Clostridiale	Ruminococ	Oscillibacte	-0.00681
Otu0030	Bacteria	Firmicutes	Clostridia	Clostridiale	Ruminococ	Oscillibacte	-0.00695
Otu0225	Bacteria	Firmicutes	Clostridia	Clostridiale	Family_XIII	Family_XIII_	-0.00712
Otu0094	Bacteria	Firmicutes	Clostridia	Clostridiale	Ruminococ	Intestinimc	-0.00743
Otu0091	Bacteria	Firmicutes	Clostridia	Clostridiale	Lachnospira	Lachnospira	-0.0082
Otu0196	Bacteria	Firmicutes	Erysipelotri	Erysipelotri	Erysipelotri	Erysipelotri	-0.00858
Otu0082	Bacteria	Firmicutes	Clostridia	Clostridiale	Lachnospira	Lachnospira	-0.0102
Otu0029	Bacteria	Firmicutes	Clostridia	Clostridiale	Lachnospira	Lachnospira	-0.01047
Otu0043	Bacteria	Firmicutes	Clostridia	Clostridiale	Ruminococ	Ruminiclos	-0.01195
Otu0208	Bacteria	Firmicutes	Clostridia	Clostridiale	Lachnospira	Lachnospira	-0.01199
Otu0127	Bacteria	Firmicutes	Clostridia	Clostridiale	Lachnospira	Lachnospira	-0.01219
Otu0120	Bacteria	Firmicutes	Clostridia	Clostridiale	Ruminococ	Ruminococ	-0.01237
Otu0067	Bacteria	Firmicutes	Clostridia	Clostridiale	Lachnospira	Lachnospira	-0.01295
Otu0078	Bacteria	Firmicutes	Clostridia	Clostridiale	Ruminococ	Ruminococ	-0.01394
Otu0118	Bacteria	Firmicutes	Clostridia	Clostridiale	Clostridiale	Clostridiale	-0.01445
Otu0049	Bacteria	Firmicutes	Bacilli	Bacillales	Bacillaceae	Bacillus	-0.01521
Otu0102	Bacteria	Firmicutes	Clostridia	Clostridiale	Ruminococ	Ruminococ	-0.01521
Otu0025	Bacteria	Firmicutes	Clostridia	Clostridiale	Lachnospira	Lachnospira	-0.01589
Otu0033	Bacteria	Firmicutes	Clostridia	Clostridiale	Lachnospira	A2	-0.01969
NA.60	NA	NA	NA	NA	NA	NA	-0.02004
Otu0124	Bacteria	Firmicutes	Clostridia	Clostridiale	Ruminococ	Ruminococ	-0.02004
Otu0004	Bacteria	Firmicutes	Bacilli	Lactobacilla	Lactobacilla	Lactobacillu	-0.02137
Otu0086	Bacteria	Firmicutes	Clostridia	Clostridiale	Lachnospira	Lachnospira	-0.02384
Otu0052	Bacteria	Firmicutes	Clostridia	Clostridiale	Lachnospira	Lachnospira	-0.02465
Otu0011	Bacteria	Firmicutes	Clostridia	Clostridiale	Lachnospira	Lachnospira	-0.02523
Otu0007	Bacteria	Firmicutes	Clostridia	Clostridiale	Ruminococ	Ruminiclos	-0.02649
Otu0041	Bacteria	Firmicutes	Clostridia	Clostridiale	Lachnospira	Lachnoclos	-0.03211
