# Supplementary material for: Oral Vaccination Using a Probiotic Vaccine Platform Combined with Prebiotics Impacts Immune Response and the Microbiome
Source: Vaccines (Basel). 2022 Sep 4;10(9):1465. doi: 10.3390/vaccines10091465 (PMC9504555; doi:10.3390/vaccines10091465)
Supplement: Supplementary file 1 [file vaccines-10-01465-s001.zip › Table_S5_whole_importance-fecal.pdf]

Table_S5_whole_importance-fecal

	kingdom	phylum	class	order	family	genus	whole_a_4
Otu0023	Bacteria	Firmicutes	Clostridia	Clostridiale	Lachnospira	Lachnospira	1
Otu0109	Bacteria	Firmicutes	Clostridia	Clostridiale	Lachnospira	uncultured	0.986265
Otu0071	Bacteria	Firmicutes	Clostridia	Clostridiale	Ruminococ	Ruminococ	0.797841
Otu0073	Bacteria	Tenericutes	Mollicutes	Anaeroplas	Anaeroplas	Anaeroplas	0.77873
Otu0157	Bacteria	Firmicutes	Clostridia	Clostridiale	Ruminococ	Oscillibacte	0.777496
Otu0061	Bacteria	Firmicutes	Clostridia	Clostridiale	Lachnospira	Lachnospira	0.715178
Otu0010	Bacteria	Firmicutes	Clostridia	Clostridiale	Lachnospira	Lachnospira	0.700986
Otu0089	Bacteria	Firmicutes	Clostridia	Clostridiale	Ruminococ	Anaerotrun	0.658964
Otu0070	Bacteria	Firmicutes	Erysipelotri	Erysipelotri	Erysipelotri	Erysipelato	0.494404
Otu0068	Bacteria	Firmicutes	Clostridia	Clostridiale	Lachnospira	Lachnospira	0.491523
Otu0100	Bacteria	Firmicutes	Clostridia	Clostridiale	Lachnospira	uncultured	0.48781
Otu0104	Bacteria	Firmicutes	Clostridia	Clostridiale	Lachnospira	Tyzzerella	0.475825
Otu0087	Bacteria	Firmicutes	Bacilli	Lactobacilla	Streptococ	Lactococcu	0.463715
Otu0057	Bacteria	Firmicutes	Clostridia	Clostridiale	Lachnospira	uncultured	0.431506
Otu0013	Bacteria	Firmicutes	Clostridia	Clostridiale	Lachnospira	Lachnospira	0.356777
Otu0037	Bacteria	Firmicutes	Clostridia	Clostridiale	Lachnospira	Lachnospira	0.351467
Otu0024	Bacteria	Firmicutes	Clostridia	Clostridiale	Ruminococ	Ruminococ	0.344948
Otu0048	Bacteria	Firmicutes	Clostridia	Clostridiale	Lachnospira	Acetatifact	0.336915
Otu0053	Bacteria	Firmicutes	Clostridia	Clostridiale	Lachnospira	Lachnospira	0.332727
Otu0040	Bacteria	Firmicutes	Clostridia	Clostridiale	Lachnospira	Lachnospira	0.301761
Otu0015	Bacteria	Firmicutes	Clostridia	Clostridiale	Lachnospira	Roseburia	0.219227
Otu0026	Bacteria	Firmicutes	Clostridia	Clostridiale	Lachnospira	Lachnospira	0.204112
Otu0051	Bacteria	Firmicutes	Clostridia	Clostridiale	Ruminococ	Ruminococ	0.199991
Otu0064	Bacteria	Firmicutes	Erysipelotri	Erysipelotri	Erysipelotri	Turicibacte	0.189668
Otu0047	Bacteria	Firmicutes	Clostridia	Clostridiale	Lachnospira	Lachnospira	0.181059
Otu0081	Bacteria	Firmicutes	Clostridia	Clostridiale	Lachnospira	Lachnoclos	0.181054
Otu0161	Bacteria	Firmicutes	Clostridia	Clostridiale	Clostridiale	Clostridiale	0.164678
Otu0009	Bacteria	Firmicutes	Clostridia	Clostridiale	Clostridiace	Clostridium	0.163278
Otu0042	Bacteria	Firmicutes	Clostridia	Clostridiale	Lachnospira	Lachnospira	0.149017
Otu0076	Bacteria	Firmicutes	Clostridia	Clostridiale	Lachnospira	uncultured	0.146486
Otu0097	Bacteria	Firmicutes	Clostridia	Clostridiale	Lachnospira	GCA-90006	0.142831
Otu0096	Bacteria	Firmicutes	Clostridia	Clostridiale	Lachnospira	uncultured	0.140324
Otu0151	Bacteria	Firmicutes	Clostridia	Clostridiale	Lachnospira	Lachnospira	0.133589
Otu0139	Bacteria	Firmicutes	Clostridia	Clostridiale	Lachnospira	Lachnospira	0.130322
Otu0168	Bacteria	Firmicutes	Clostridia	Clostridiale	Clostridiale	Clostridiale	0.121859
Otu0184	Bacteria	Firmicutes	Clostridia	Clostridiale	Lachnospira	Lachnospira	0.108888
Otu0091	Bacteria	Firmicutes	Clostridia	Clostridiale	Lachnospira	Lachnospira	0.104076
Otu0018	Bacteria	Firmicutes	Clostridia	Clostridiale	Ruminococ	Ruminococ	0.10148
Otu0142	Bacteria	Firmicutes	Clostridia	Clostridiale	Lachnospira	Lachnospira	0.098501
Otu0075	Bacteria	Firmicutes	Clostridia	Clostridiale	Lachnospira	Lachnospira	0.093059
Otu0036	Bacteria	Firmicutes	Clostridia	Clostridiale	Lachnospira	Lachnospira	0.091861
Otu0012	Bacteria	Firmicutes	Clostridia	Clostridiale	Lachnospira	Lachnospira	0.087298
Otu0195	Bacteria	Firmicutes	Clostridia	Clostridiale	Clostridiale	Clostridiale	0.086837
Otu0088	Bacteria	Firmicutes	Clostridia	Clostridiale	Ruminococ	Butyricicoc	0.086435
Otu0006	Bacteria	Firmicutes	Clostridia	Clostridiale	Lachnospira	Lachnospira	0.083263

Otu0111	Bacteria	Firmicutes	Clostridia	Clostridiale	Lachnospira	Lachnospira	0.078221
Otu0190	Bacteria	Firmicutes	Clostridia	Clostridiale	Lachnospira	Lachnospira	0.077403
Otu0162	Bacteria	Firmicutes	Clostridia	Clostridiale	Lachnospira	Lachnospira	0.07721
Otu0153	Bacteria	Firmicutes	Clostridia	Clostridiale	Ruminococ	Ruminococ	0.076318
Otu0106	Bacteria	Firmicutes	Clostridia	Clostridiale	Peptococca	uncultured	0.069227
Otu0084	Bacteria	Firmicutes	Clostridia	Clostridiale	Ruminococ	Ruminiclos	0.068597
Otu0028	Bacteria	Firmicutes	Clostridia	Clostridiale	Lachnospira	Lachnospira	0.064591
Otu0016	Bacteria	Firmicutes	Clostridia	Clostridiale	Lachnospira	Lachnospira	0.060983
Otu0159	Bacteria	Firmicutes	Clostridia	Clostridiale	Ruminococ	Ruminococ	0.058549
Otu0098	Bacteria	Firmicutes	Clostridia	Clostridiale	Ruminococ	Ruminococ	0.057557
Otu0020	Bacteria	Firmicutes	Clostridia	Clostridiale	Lachnospira	Lachnospira	0.054215
Otu0212	Bacteria	Firmicutes	Clostridia	Clostridiale	Ruminococ	Ruminococ	0.050373
Otu0011	Bacteria	Firmicutes	Clostridia	Clostridiale	Lachnospira	Lachnospira	0.047623
Otu0027	Bacteria	Firmicutes	Clostridia	Clostridiale	Lachnospira	Acetatifact	0.044714
Otu0095	Bacteria	Firmicutes	Clostridia	Clostridiale	Lachnospira	Lachnospira	0.044558
Otu0115	Bacteria	Firmicutes	Clostridia	Clostridiale	Lachnospira	Tyzzerella_	0.044352
Otu0029	Bacteria	Firmicutes	Clostridia	Clostridiale	Lachnospira	Lachnospira	0.043583
Otu0149	Bacteria	Firmicutes	Clostridia	Clostridiale	Ruminococ	Ruminococ	0.041736
Otu0017	Bacteria	Firmicutes	Bacilli	Lactobacilla	Lactobacilla	Lactobacillu	0.040728
Otu0164	Bacteria	Firmicutes	Clostridia	Clostridiale	Ruminococ	Ruminococ	0.039596
Otu0019	Bacteria	Firmicutes	Clostridia	Clostridiale	Ruminococ	Ruminiclos	0.037651
Otu0155	Bacteria	Firmicutes	Clostridia	Clostridiale	Ruminococ	Anaerotrur	0.035314
Otu0178	Bacteria	Firmicutes	Clostridia	Clostridiale	Lachnospira	Lachnospira	0.03289
Otu0171	Bacteria	Firmicutes	Clostridia	Clostridiale	Ruminococ	Ruminococ	0.032788
Otu0031	Bacteria	Firmicutes	Clostridia	Clostridiale	Lachnospira	Marvinbrya	0.031412
Otu0173	Bacteria	Firmicutes	Clostridia	Clostridiale	Ruminococ	Ruminococ	0.03091
Otu0083	Bacteria	Firmicutes	Clostridia	Clostridiale	Ruminococ	Ruminococ	0.028143
Otu0137	Bacteria	Firmicutes	Clostridia	Clostridiale	Lachnospira	Lachnospira	0.027853
Otu0121	Bacteria	Firmicutes	Clostridia	Clostridiale	Lachnospira	Lachnospira	0.026869
Otu0086	Bacteria	Firmicutes	Clostridia	Clostridiale	Lachnospira	Lachnospira	0.026031
Otu0174	Bacteria	Firmicutes	Clostridia	Clostridiale	Lachnospira	Lachnospira	0.025641
Otu0130	Bacteria	Firmicutes	Clostridia	Clostridiale	Ruminococ	Oscillibacte	0.023628
Otu0055	Bacteria	Firmicutes	Clostridia	Clostridiale	Lachnospira	A2	0.023394
Otu0035	Bacteria	Proteobact	Gammapro	Enterobact	Enterobact	Enterobact	0.022122
Otu0146	Bacteria	Firmicutes	Clostridia	Clostridiale	Lachnospira	Lachnospira	0.021789
Otu0144	Bacteria	Firmicutes	Clostridia	Clostridiale	Lachnospira	Lachnospira	0.021667
Otu0110	Bacteria	Firmicutes	Clostridia	Clostridiale	Lachnospira	Lachnospira	0.020556
Otu0078	Bacteria	Firmicutes	Clostridia	Clostridiale	Ruminococ	Ruminococ	0.019954
Otu0129	Bacteria	Firmicutes	Clostridia	Clostridiale	Ruminococ	Ruminococ	0.018902
Otu0189	Bacteria	Firmicutes	Clostridia	Clostridiale	Lachnospira	Lachnospira	0.017437
Otu0193	Bacteria	Firmicutes	Clostridia	Clostridiale	Ruminococ	Ruminiclos	0.017413
Otu0202	Bacteria	Firmicutes	Clostridia	Clostridiale	Clostridiale	Clostridiale	0.016698
Otu0090	Bacteria	Firmicutes	Clostridia	Clostridiale	Ruminococ	Ruminococ	0.016095
Otu0134	Bacteria	Firmicutes	Clostridia	Clostridiale	Lachnospira	Lachnospira	0.016033
Otu0141	Bacteria	Actinobact	Coriobacte	Coriobacte	Eggerthella	Eggerthella	0.015963
Otu0003	Bacteria	Firmicutes	Clostridia	Clostridiale	Lachnospira	Lachnospira	0.015735
Otu0079	Bacteria	Firmicutes	Clostridia	Clostridiale	Lachnospira	Lachnospira	0.015678

Otu0056	Bacteria	Firmicutes	Clostridia	Clostridiale	Lachnospira	uncultured	0.015672
Otu0183	Bacteria	Firmicutes	Clostridia	Clostridiale	Ruminococ	Ruminiclos	0.015511
Otu0001	Bacteria	Bacteroide	Bacteroidia	Bacteroidal	Muribacula	Muribacula	0.015172
Otu0192	Bacteria	Firmicutes	Clostridia	Clostridiale	Lachnospira	Lachnospira	0.014953
Otu0116	Bacteria	Firmicutes	Clostridia	Clostridiale	Lachnospira	Lachnospira	0.014708
Otu0166	Bacteria	Firmicutes	Clostridia	Clostridiale	Ruminococ	Oscillibacte	0.013977
Otu0118	Bacteria	Firmicutes	Clostridia	Clostridiale	Clostridiale	Clostridiale	0.013439
Otu0099	Bacteria	Actinobacte	Coriobacte	Coriobacte	Eggerthella	Adlercreutz	0.013032
Otu0092	Bacteria	Firmicutes	Clostridia	Clostridiale	Lachnospira	Lachnospira	0.012929
Otu0120	Bacteria	Firmicutes	Clostridia	Clostridiale	Ruminococ	Ruminococ	0.012664
Otu0145	Bacteria	Firmicutes	Clostridia	Clostridiale	Lachnospira	Lachnospira	0.012052
Otu0043	Bacteria	Firmicutes	Clostridia	Clostridiale	Ruminococ	Ruminiclos	0.011127
Otu0147	Bacteria	Firmicutes	Clostridia	Clostridiale	Lachnospira	Lachnospira	0.011042
Otu0108	Bacteria	Firmicutes	Clostridia	Clostridiale	Lachnospira	Lachnospira	0.010955
Otu0117	Bacteria	Firmicutes	Clostridia	Clostridiale	Ruminococ	Ruminiclos	0.010484
Otu0182	Bacteria	Firmicutes	Clostridia	Clostridiale	Lachnospira	Lachnospira	0.010378
Otu0065	Bacteria	Firmicutes	Bacilli	Lactobacilla	Enterococc	Enterococc	0.010241
Otu0102	Bacteria	Firmicutes	Clostridia	Clostridiale	Ruminococ	Ruminococ	0.010223
Otu0257	Bacteria	Firmicutes	Clostridia	Clostridiale	Ruminococ	Ruminiclos	0.010125
Otu0112	Bacteria	Firmicutes	Clostridia	Clostridiale	Lachnospira	Lachnospira	0.009337
Otu0158	Bacteria	Firmicutes	Clostridia	Clostridiale	Ruminococ	Ruminococ	0.009294
Otu0255	Bacteria	Firmicutes	Clostridia	Clostridiale	Ruminococ	Oscillibacte	0.009148
Otu0196	Bacteria	Firmicutes	Erysipelotri	Erysipelotri	Erysipelotri	Erysipelotri	0.00901
Otu0234	Bacteria	Bacteroide	Bacteroidia	Bacteroidal	Muribacula	Muribacula	0.00897
Otu0005	Bacteria	Bacteroide	Bacteroidia	Bacteroidal	Muribacula	Muribacula	0.008925
Otu0030	Bacteria	Firmicutes	Clostridia	Clostridiale	Ruminococ	Oscillibacte	0.008556
Otu0180	Bacteria	Firmicutes	Clostridia	Clostridiale	Ruminococ	Oscillibacte	0.007668
Otu0025	Bacteria	Firmicutes	Clostridia	Clostridiale	Lachnospira	Lachnospira	0.007207
Otu0179	Bacteria	Firmicutes	Clostridia	Clostridiale	Lachnospira	Lachnospira	0.007047
Otu0216	Bacteria	Firmicutes	Clostridia	Clostridiale	Lachnospira	Lachnospira	0.006945
Otu0069	Bacteria	Firmicutes	Clostridia	Clostridiale	Lachnospira	uncultured	0.006728
Otu0235	Bacteria	Firmicutes	Clostridia	Clostridiale	Clostridiale	Clostridiale	0.006388
Otu0058	Bacteria	Firmicutes	Clostridia	Clostridiale	Lachnospira	uncultured	0.006387
Otu0222	Bacteria	Firmicutes	Erysipelotri	Erysipelotri	Erysipelotri	Candidatus	0.006122
Otu0114	Bacteria	Firmicutes	Clostridia	Clostridiale	Lachnospira	Lachnospira	0.005893
Otu0077	Bacteria	Firmicutes	Clostridia	Clostridiale	Ruminococ	Ruminiclos	0.005684
Otu0059	Bacteria	Firmicutes	Clostridia	Clostridiale	Lachnospira	GCA-90006	0.005679
Otu0259	Bacteria	Tenericutes	Mollicutes	Mollicutes_	Mollicutes_	Mollicutes_	0.005605
Otu0186	Bacteria	Firmicutes	Clostridia	Clostridiale	Lachnospira	Lachnospira	0.005267
Otu0004	Bacteria	Firmicutes	Bacilli	Lactobacilla	Lactobacilla	Lactobacillu	0.005225
Otu0243	Bacteria	Firmicutes	Clostridia	Clostridiale	Lachnospira	Lachnospira	0.004757
Otu0245	Bacteria	Firmicutes	Clostridia	Clostridiale	Ruminococ	Ruminococ	0.004607
Otu0150	Bacteria	Firmicutes	Clostridia	Clostridiale	Lachnospira	Lachnospira	0.004483
Otu0066	Bacteria	Firmicutes	Clostridia	Clostridiale	Lachnospira	Lachnospira	0.003853
Otu0185	Bacteria	Firmicutes	Clostridia	Clostridiale	Ruminococ	Ruminococ	0.003768
Otu0229	Bacteria	Firmicutes	Clostridia	Clostridiale	Lachnospira	Lachnospira	0.003768
Otu0163	Bacteria	Firmicutes	Clostridia	Clostridiale	Lachnospira	Lachnospira	0.003742

Otu0218	Bacteria	Firmicutes	Clostridia	Clostridiale	Ruminococ	Ruminococ	0.003643
Otu0107	Bacteria	Proteobact	Alphaprote	Rhizobiales	Rhizobiacea	Brucella	0.00355
Otu0236	Bacteria	Firmicutes	Clostridia	Clostridiale	Ruminococ	Ruminiclos	0.003345
Otu0228	Bacteria	Tenericutes	Mollicutes	Mollicutes_	Mollicutes_	Mollicutes_	0.003245
Otu0230	Bacteria	Firmicutes	Clostridia	Clostridiale	Lachnospira	Lachnospira	0.003181
Otu0062	Bacteria	Proteobact	Alphaprote	Rhizobiales	Rhizobiacea	Rhizobiacea	0.003053
Otu0154	Bacteria	Firmicutes	Clostridia	Clostridiale	Lachnospira	Lachnospira	0.002947
Otu0214	Bacteria	Firmicutes	Clostridia	Clostridiale	Lachnospira	Lachnospira	0.002856
Otu0022	Bacteria	Proteobact	Gammapro	Enterobact	Enterobact	Escherichia	0.002191
Otu0253	Bacteria	Firmicutes	Clostridia	Clostridiale	Clostridiale	Clostridiale	0.002055
Otu0254	Bacteria	Firmicutes	Clostridia	Clostridiale	Lachnospira	Lachnospira	0.002055
Otu0293	Bacteria	Tenericutes	Mollicutes	Mollicutes_	Mollicutes_	Mollicutes_	0.002055
Otu0207	Bacteria	Firmicutes	Clostridia	Clostridiale	Lachnospira	Lachnospira	0.001927
Otu0105	Bacteria	Firmicutes	Clostridia	Clostridiale	Lachnospira	Lachnospira	0.001922
Otu0119	Bacteria	Firmicutes	Clostridia	Clostridiale	Ruminococ	Ruminiclos	0.001914
Otu0032	Bacteria	Firmicutes	Clostridia	Clostridiale	Ruminococ	Ruminiclos	0.001906
Otu0177	Bacteria	Firmicutes	Clostridia	Clostridiale	Lachnospira	Lachnospira	0.001833
Otu0237	Bacteria	Firmicutes	Clostridia	Clostridiale	Lachnospira	Lachnospira	0.001814
Otu0264	Bacteria	Firmicutes	Clostridia	Clostridiale	Clostridiale	Clostridiale	0.001814
Otu0232	Bacteria	Firmicutes	Clostridia	Clostridiale	Ruminococ	Ruminococ	0.001713
Otu0247	Bacteria	Firmicutes	Clostridia	Clostridiale	Clostridiale	Clostridiale	0.001713
Otu0284	Bacteria	Firmicutes	Clostridia	Clostridiale	Ruminococ	Ruminococ	0.001713
Otu0294	Bacteria	Firmicutes	Clostridia	Clostridiale	Ruminococ	Ruminococ	0.001623
Otu0260	Bacteria	Firmicutes	Firmicutes_	Firmicutes_	Firmicutes_	Firmicutes_	0.001468
Otu0175	Bacteria	Firmicutes	Clostridia	Clostridiale	Ruminococ	Ruminococ	0.001288
Otu0220	Bacteria	Firmicutes	Clostridia	Clostridiale	Lachnospira	Lachnospira	0.001285
Otu0169	Bacteria	Firmicutes	Erysipelotri	Erysipelotri	Erysipelotri	Erysipelotri	0.00126
time	NA	NA	NA	NA	NA	NA	0.001105
Otu0215	Bacteria	Tenericutes	Mollicutes	Mollicutes_	Mollicutes_	Mollicutes_	0.001027
Otu0125	Bacteria	Firmicutes	Erysipelotri	Erysipelotri	Erysipelotri	uncultured	0.000961
Otu0074	Bacteria	Firmicutes	Clostridia	Clostridiale	Lachnospira	Lachnospira	0.000894
Otu0191	Bacteria	Firmicutes	Clostridia	Clostridiale	Clostridiale	Clostridiale	0.000878
Otu0094	Bacteria	Firmicutes	Clostridia	Clostridiale	Ruminococ	Intestinimo	0.000495
Otu0044	Bacteria	Firmicutes	Bacilli	Bacillales	Listeriaceae	Listeria	0
Otu0072	Bacteria	Proteobact	Gammapro	Pseudomo	Pseudomo	Pseudomo	0
Otu0113	Bacteria	Firmicutes	Bacilli	Lactobacilla	Lactobacilla	Lactobacillu	0
Otu0131	Bacteria	Proteobact	Alphaprote	Caulobacte	Caulobacte	Brevundim	0
Otu0132	Bacteria	Actinobact	Actinobact	Micrococca	Micrococca	Micrococca	0
Otu0136	Bacteria	Actinobact	Actinobact	Corynebact	Nocardiace	Rhodococc	0
Otu0143	Bacteria	Proteobact	Gammapro	Xanthomor	Xanthomor	Stenotroph	0
Otu0156	Bacteria	Actinobact	Actinobact	Micrococca	Microbacte	Curtobacte	0
Otu0165	Bacteria	Firmicutes	Bacilli	Bacillales	Bacillaceae	Bacillus	0
Otu0188	Bacteria	Proteobact	Alphaprote	Rhizobiales	Rhizobiacea	Mesorhizol	0
Otu0194	Bacteria	Firmicutes	Clostridia	Clostridiale	Lachnospira	Lachnospira	0
Otu0201	Bacteria	Firmicutes	Clostridia	Clostridiale	Ruminococ	Anaerotrur	0
Otu0204	Bacteria	Actinobact	Actinobact	Corynebact	Nocardiace	Gordonia	0
Otu0211	Bacteria	Firmicutes	Clostridia	Clostridiale	Lachnospira	Lachnospira	0

Otu0213	Bacteria	Firmicutes	Clostridia	Clostridiale	Clostridiale	Clostridiale	0
Otu0219	Bacteria	Verrucomi	Verrucomi	Verrucomi	Akkermans	Akkermans	0
Otu0223	Bacteria	Firmicutes	Clostridia	Clostridiale	Lachnospir	Lachnospir	0
Otu0226	Bacteria	Firmicutes	Clostridia	Clostridiale	Lachnospir	Lachnospir	0
Otu0227	Bacteria	Firmicutes	Clostridia	Clostridiale	Ruminococ	Ruminococ	0
Otu0238	Bacteria	Proteobact	Alphaprote	Rhizobiales	Xanthobact	Bradyrhizol	0
Otu0239	Bacteria	Firmicutes	Clostridia	Clostridiale	Ruminococ	Ruminococ	0
Otu0240	Bacteria	Firmicutes	Clostridia	Clostridiale	Lachnospir	Lachnospir	0
Otu0242	Bacteria	Proteobact	Gammapro	Betaprotec	Burkholder	Ralstonia	0
Otu0248	Bacteria	Firmicutes	Clostridia	Clostridiale	Ruminococ	Ruminococ	0
Otu0249	Bacteria	Firmicutes	Clostridia	Clostridiale	Ruminococ	Ruminococ	0
Otu0250	Bacteria	Firmicutes	Clostridia	Clostridiale	Lachnospir	Lachnospir	0
Otu0251	Bacteria	Firmicutes	Clostridia	Clostridiale	Ruminococ	Ruminiclos	0
Otu0252	Bacteria	Firmicutes	Clostridia	Clostridiale	Lachnospir	Lachnospir	0
Otu0261	Bacteria	Firmicutes	Clostridia	Clostridiale	Lachnospir	Lachnospir	0
Otu0262	Bacteria	Firmicutes	Clostridia	Clostridiale	Ruminococ	Ruminiclos	0
Otu0263	Bacteria	Firmicutes	Clostridia	Clostridiale	Family_XIII	Family_XIII_	0
Otu0266	Bacteria	Firmicutes	Clostridia	Clostridiale	Ruminococ	Ruminococ	0
Otu0267	Bacteria	Firmicutes	Bacilli	Lactobacilla	Lactobacilla	Lactobacillu	0
Otu0269	Bacteria	Bacteroide	Bacteroidia	Bacteroidal	Muribacula	Muribacula	0
Otu0270	Bacteria	Firmicutes	Clostridia	Clostridiale	Lachnospir	Lachnospir	0
Otu0271	Bacteria	Firmicutes	Clostridia	Clostridiale	Clostridiale	Clostridiale	0
Otu0273	Bacteria	Firmicutes	Clostridia	Clostridiale	Clostridiale	Clostridiale	0
Otu0274	Bacteria	Firmicutes	Clostridia	Clostridiale	Clostridiale	Clostridiale	0
Otu0276	Bacteria	Firmicutes	Clostridia	Clostridiale	Ruminococ	Ruminococ	0
Otu0277	Bacteria	Firmicutes	Clostridia	Clostridiale	Lachnospir	Lachnospir	0
Otu0278	Bacteria	Actinobact	Coriobacte	Coriobacte	Eggerthella	Eggerthella	0
Otu0279	Bacteria	Bacteroide	Bacteroidia	Bacteroidal	Muribacula	Muribacula	0
Otu0280	Bacteria	Firmicutes	Clostridia	Clostridiale	Lachnospir	Lachnospir	0
NA.1	NA	NA	NA	NA	NA	NA	0
Otu0283	Bacteria	Bacteroide	Bacteroidia	Bacteroidal	Muribacula	Muribacula	0
Otu0285	Bacteria	Proteobact	Alphaprote	Rhizobiales	Beijerinckia	Methyloba	0
Otu0286	Bacteria	Firmicutes	Clostridia	Clostridiale	Ruminococ	Ruminococ	0
Otu0287	Bacteria	Firmicutes	Clostridia	Clostridiale	Ruminococ	Butyricicoc	0
Otu0288	Bacteria	Firmicutes	Clostridia	Clostridiale	Lachnospir	Lachnospir	0
NA.2	NA	NA	NA	NA	NA	NA	0
Otu0290	Bacteria	Firmicutes	Clostridia	Clostridiale	Clostridiale	Clostridiale	0
NA.3	NA	NA	NA	NA	NA	NA	0
Otu0292	Bacteria	Firmicutes	Clostridia	Clostridiale	Lachnospir	Lachnospir	0
NA.4	NA	NA	NA	NA	NA	NA	0
Otu0296	Bacteria	Firmicutes	Clostridia	Clostridiale	Ruminococ	Ruminococ	0
Otu0297	Bacteria	Proteobact	Gammapro	Pseudomo	Pseudomo	Pseudomo	0
NA.5	NA	NA	NA	NA	NA	NA	0
Otu0299	Bacteria	Firmicutes	Clostridia	Clostridiale	Ruminococ	Ruminococ	0
NA.6	NA	NA	NA	NA	NA	NA	0
Otu0302	Bacteria	Firmicutes	Clostridia	Clostridiale	Ruminococ	Ruminococ	0
Otu0303	Bacteria	Firmicutes	Clostridia	Clostridiale	Lachnospir	Lachnospir	0

Otu0304	Bacteria	Firmicutes	Clostridia	Clostridiale	Ruminococ	Flavonifrac	0
Otu0305	Bacteria	Firmicutes	Clostridia	Clostridiale	Lachnospir	Lachnospir	0
Otu0306	Bacteria	Firmicutes	Clostridia	Clostridiale	Lachnospir	Lachnospir	0
NA.7	NA	NA	NA	NA	NA	NA	0
NA.8	NA	NA	NA	NA	NA	NA	0
Otu0312	Bacteria	Firmicutes	Clostridia	Clostridiale	Ruminococ	Ruminiclos	0
NA.9	NA	NA	NA	NA	NA	NA	0
Otu0317	Bacteria	Firmicutes	Clostridia	Clostridiale	Lachnospir	Lachnospir	0
Otu0318	Bacteria	Firmicutes	Clostridia	Clostridiale	Ruminococ	Ruminococ	0
Otu0321	Bacteria	Firmicutes	Clostridia	Clostridiale	Ruminococ	Ruminiclos	0
Otu0322	Bacteria	Firmicutes	Clostridia	Clostridiale	Lachnospir	Lachnospir	0
Otu0323	Bacteria	Firmicutes	Clostridia	Clostridiale	Lachnospir	Lachnospir	0
Otu0324	Bacteria	Firmicutes	Clostridia	Clostridiale	Lachnospir	Lachnospir	0
Otu0328	Bacteria	Bacteroide	Bacteroidia	Bacteroidal	Muribacula	Muribacula	0
NA.10	NA	NA	NA	NA	NA	NA	0
Otu0331	Bacteria	Firmicutes	Clostridia	Clostridiale	Lachnospir	Acetatifact	0
NA.11	NA	NA	NA	NA	NA	NA	0
Otu0334	Bacteria	Firmicutes	Clostridia	Clostridiale	Lachnospir	Lachnospir	0
Otu0335	Bacteria	Firmicutes	Clostridia	Clostridiale	Ruminococ	Ruminococ	0
NA.12	NA	NA	NA	NA	NA	NA	0
Otu0338	Bacteria	Bacteroide	Bacteroidia	Bacteroidal	Muribacula	Muribacula	0
Otu0339	Bacteria	Firmicutes	Clostridia	Clostridiale	Lachnospir	Acetatifact	0
Otu0342	Bacteria	Firmicutes	Clostridia	Clostridiale	Clostridiale	Clostridiale	0
NA.13	NA	NA	NA	NA	NA	NA	0
Otu0346	Bacteria	Firmicutes	Clostridia	Clostridiale	Ruminococ	Ruminococ	0
Otu0347	Bacteria	Firmicutes	Clostridia	Clostridiale	Lachnospir	Lachnospir	0
NA.14	NA	NA	NA	NA	NA	NA	0
Otu0351	Bacteria	Firmicutes	Clostridia	Clostridiale	Clostridiale	Clostridiale	0
NA.15	NA	NA	NA	NA	NA	NA	0
Otu0353	Bacteria	Firmicutes	Clostridia	Clostridiale	Ruminococ	Ruminiclos	0
Otu0356	Bacteria	Firmicutes	Clostridia	Clostridiale	Lachnospir	Lachnospir	0
Otu0358	Bacteria	Firmicutes	Clostridia	Clostridiale	Ruminococ	Ruminiclos	0
NA.16	NA	NA	NA	NA	NA	NA	0
Otu0361	Bacteria	Tenericutes	Mollicutes	Mollicutes_	Mollicutes_	Mollicutes_	0
Otu0366	Bacteria	Firmicutes	Clostridia	Clostridiale	Lachnospir	Lachnospir	0
Otu0369	Bacteria	Firmicutes	Clostridia	Clostridiale	Ruminococ	Ruminococ	0
Otu0370	Bacteria	Tenericutes	Mollicutes	Mollicutes_	Mollicutes_	Mollicutes_	0
NA.17	NA	NA	NA	NA	NA	NA	0
Otu0376	Bacteria	Firmicutes	Clostridia	Clostridiale	Lachnospir	Lachnospir	0
NA.18	NA	NA	NA	NA	NA	NA	0
NA.19	NA	NA	NA	NA	NA	NA	0
NA.20	NA	NA	NA	NA	NA	NA	0
Otu0384	Bacteria	Proteobact	Gammaprc	Pseudomoı	Moraxellac	Acinetobac	0
Otu0386	Bacteria	Tenericutes	Mollicutes	Mollicutes_	Mollicutes_	Mollicutes_	0
NA.21	NA	NA	NA	NA	NA	NA	0
NA.22	NA	NA	NA	NA	NA	NA	0
NA.23	NA	NA	NA	NA	NA	NA	0

NA.24	NA	NA	NA	NA	NA	NA	0
NA.25	NA	NA	NA	NA	NA	NA	0
Otu0405	Bacteria	Firmicutes	Clostridia	Clostridiale	Lachnospira	Lachnospira	0
Otu0406	Bacteria	Firmicutes	Clostridia	Clostridiale	Lachnospira	Lachnospira	0
Otu0410	Bacteria	Firmicutes	Clostridia	Clostridiale	Ruminococ	Ruminococ	0
NA.26	NA	NA	NA	NA	NA	NA	0
NA.27	NA	NA	NA	NA	NA	NA	0
NA.28	NA	NA	NA	NA	NA	NA	0
NA.29	NA	NA	NA	NA	NA	NA	0
NA.30	NA	NA	NA	NA	NA	NA	0
NA.31	NA	NA	NA	NA	NA	NA	0
NA.32	NA	NA	NA	NA	NA	NA	0
NA.33	NA	NA	NA	NA	NA	NA	0
Otu0439	Bacteria	Firmicutes	Clostridia	Clostridiale	Lachnospira	Lachnospira	0
Otu0441	Bacteria	Proteobact	Alphaprote	Rhizobiales	Beijerinckia	Methyloba	0
NA.34	NA	NA	NA	NA	NA	NA	0
Otu0447	Bacteria	Tenericutes	Mollicutes	Mollicutes_	Mollicutes_	Mollicutes_	0
NA.35	NA	NA	NA	NA	NA	NA	0
NA.36	NA	NA	NA	NA	NA	NA	0
Otu0452	Bacteria	Firmicutes	Clostridia	Clostridiale	Lachnospira	Lachnospira	0
NA.37	NA	NA	NA	NA	NA	NA	0
Otu0456	Bacteria	Firmicutes	Clostridia	Clostridiale	Lachnospira	Lachnospira	0
Otu0459	Bacteria	Tenericutes	Mollicutes	Mollicutes_	Mollicutes_	Mollicutes_	0
NA.38	NA	NA	NA	NA	NA	NA	0
NA.39	NA	NA	NA	NA	NA	NA	0
NA.40	NA	NA	NA	NA	NA	NA	0
NA.41	NA	NA	NA	NA	NA	NA	0
NA.42	NA	NA	NA	NA	NA	NA	0
NA.43	NA	NA	NA	NA	NA	NA	0
NA.44	NA	NA	NA	NA	NA	NA	0
NA.45	NA	NA	NA	NA	NA	NA	0
NA.46	NA	NA	NA	NA	NA	NA	0
NA.47	NA	NA	NA	NA	NA	NA	0
NA.48	NA	NA	NA	NA	NA	NA	0
NA.49	NA	NA	NA	NA	NA	NA	0
Otu0506	Bacteria	Firmicutes	Clostridia	Clostridiale	Lachnospira	Lachnospira	0
NA.50	NA	NA	NA	NA	NA	NA	0
NA.51	NA	NA	NA	NA	NA	NA	0
NA.52	NA	NA	NA	NA	NA	NA	0
NA.53	NA	NA	NA	NA	NA	NA	0
NA.54	NA	NA	NA	NA	NA	NA	0
Otu0545	Bacteria	Firmicutes	Clostridia	Clostridiale	Lachnospira	Lachnospira	0
NA.55	NA	NA	NA	NA	NA	NA	0
Otu0557	Bacteria	Firmicutes	Clostridia	Clostridiale	Lachnospira	Lachnospira	0
Otu0558	Bacteria	Tenericutes	Mollicutes	Mollicutes_	Mollicutes_	Mollicutes_	0
Otu0561	Bacteria	Proteobact	Alphaprote	Rhizobiales	Methylopil	Methylopil	0
NA.56	NA	NA	NA	NA	NA	NA	0

NA.57	NA	NA	NA	NA	NA	NA	0
NA.58	NA	NA	NA	NA	NA	NA	0
NA.59	NA	NA	NA	NA	NA	NA	0
NA.60	NA	NA	NA	NA	NA	NA	0
NA.61	NA	NA	NA	NA	NA	NA	0
NA.62	NA	NA	NA	NA	NA	NA	0
NA.63	NA	NA	NA	NA	NA	NA	0
Otu0607	Bacteria	Firmicutes	Clostridia	Clostridiale	Lachnospir	Lachnospir	0
NA.64	NA	NA	NA	NA	NA	NA	0
NA.65	NA	NA	NA	NA	NA	NA	0
NA.66	NA	NA	NA	NA	NA	NA	0
NA.67	NA	NA	NA	NA	NA	NA	0
NA.68	NA	NA	NA	NA	NA	NA	0
NA.69	NA	NA	NA	NA	NA	NA	0
NA.70	NA	NA	NA	NA	NA	NA	0
NA.71	NA	NA	NA	NA	NA	NA	0
NA.72	NA	NA	NA	NA	NA	NA	0
NA.73	NA	NA	NA	NA	NA	NA	0
NA.74	NA	NA	NA	NA	NA	NA	0
NA.75	NA	NA	NA	NA	NA	NA	0
NA.76	NA	NA	NA	NA	NA	NA	0
NA.77	NA	NA	NA	NA	NA	NA	0
NA.78	NA	NA	NA	NA	NA	NA	0
Otu0751	Bacteria	Firmicutes	Clostridia	Clostridiale	Lachnospir	Lachnospir	0
NA.79	NA	NA	NA	NA	NA	NA	0
NA.80	NA	NA	NA	NA	NA	NA	0
NA.81	NA	NA	NA	NA	NA	NA	0
NA.82	NA	NA	NA	NA	NA	NA	0
NA.83	NA	NA	NA	NA	NA	NA	0
Otu0135	Bacteria	Firmicutes	Clostridia	Clostridiale	Lachnospir	Lachnospir	-0.0002
Otu0124	Bacteria	Firmicutes	Clostridia	Clostridiale	Ruminococ	Ruminococ	-0.00082
Otu0103	Bacteria	Firmicutes	Clostridia	Clostridiale	Lachnospir	Lachnoclos	-0.00098
Otu0128	Bacteria	Firmicutes	Clostridia	Clostridiale	Family_XIII	Family_XIII	-0.00127
Otu0172	Bacteria	Firmicutes	Clostridia	Clostridiale	Lachnospir	Lachnospir	-0.0014
Otu0225	Bacteria	Firmicutes	Clostridia	Clostridiale	Family_XIII	Family_XIII	-0.00147
Otu0049	Bacteria	Firmicutes	Bacilli	Bacillales	Bacillaceae	Bacillus	-0.00154
Otu0181	Bacteria	Firmicutes	Clostridia	Clostridiale	Ruminococ	Ruminococ	-0.00154
Otu0224	Bacteria	Firmicutes	Clostridia	Clostridiale	Lachnospir	Lachnospir	-0.00154
Otu0060	Bacteria	Firmicutes	Clostridia	Clostridiale	Peptostrep	Romboutsi	-0.00162
Otu0246	Bacteria	Firmicutes	Clostridia	Clostridiale	Family_XIII	Family_XIII	-0.00162
Otu0160	Bacteria	Firmicutes	Clostridia	Clostridiale	Ruminococ	Ruminococ	-0.00171
Otu0256	Bacteria	Firmicutes	Clostridia	Clostridiale	Lachnospir	Lachnospir	-0.00171
Otu0045	Bacteria	Firmicutes	Bacilli	Bacillales	Staphyloco	Staphyloco	-0.00173
Otu0233	Bacteria	Firmicutes	Clostridia	Clostridiale	Lachnospir	Lachnospir	-0.00195
Otu0138	Bacteria	Firmicutes	Clostridia	Clostridiale	Ruminococ	Ruminococ	-0.00199
Otu0221	Bacteria	Firmicutes	Clostridia	Clostridiale	Ruminococ	GCA-90006	-0.00206
Otu0080	Bacteria	Firmicutes	Clostridia	Clostridiale	Lachnospir	uncultured	-0.00221

Otu0209	Bacteria	Firmicutes	Clostridia	Clostridiale	Peptococca	uncultured	-0.00235
Otu0272	Bacteria	Firmicutes	Clostridia	Clostridiale	Ruminococ	Ruminococ	-0.00315
NA.84	NA	NA	NA	NA	NA	NA	-0.00335
NA.85	NA	NA	NA	NA	NA	NA	-0.00352
Otu0231	Bacteria	Firmicutes	Clostridia	Clostridiale	Lachnospira	Lachnospira	-0.00363
Otu0206	Bacteria	Firmicutes	Clostridia	Clostridiale	Ruminococ	Ruminococ	-0.0038
Otu0082	Bacteria	Firmicutes	Clostridia	Clostridiale	Lachnospira	Lachnospira	-0.00395
Otu0208	Bacteria	Firmicutes	Clostridia	Clostridiale	Lachnospira	Lachnospira	-0.00398
Otu0039	Bacteria	Firmicutes	Clostridia	Clostridiale	Lachnospira	Lachnospira	-0.00423
Otu0002	Bacteria	Bacteroide	Bacteroidia	Bacteroidal	Muribacula	Muribacula	-0.00469
Otu0205	Bacteria	Firmicutes	Clostridia	Clostridiale	Ruminococ	Ruminococ	-0.0049
Otu0199	Bacteria	Firmicutes	Clostridia	Clostridiale	Lachnospira	Lachnospira	-0.00601
Otu0046	Bacteria	Firmicutes	Clostridia	Clostridiale	Lachnospira	Lachnospira	-0.00602
Otu0170	Bacteria	Firmicutes	Clostridia	Clostridiale	Lachnospira	GCA-90006	-0.00649
Otu0200	Bacteria	Firmicutes	Clostridia	Clostridiale	Ruminococ	Ruminococ	-0.00677
Otu0123	Bacteria	Firmicutes	Clostridia	Clostridiale	Lachnospira	Lachnospira	-0.00714
Otu0050	Bacteria	Firmicutes	Clostridia	Clostridiale	Ruminococ	Oscillibacte	-0.00739
Otu0265	Bacteria	Firmicutes	Clostridia	Clostridiale	Lachnospira	Tyzzerella_	-0.00793
Otu0198	Bacteria	Firmicutes	Clostridia	Clostridiale	Clostridiale	Clostridiale	-0.00797
Otu0133	Bacteria	Firmicutes	Clostridia	Clostridiale	Ruminococ	Ruminococ	-0.00832
Otu0038	Bacteria	Firmicutes	Clostridia	Clostridiale	Lachnospira	Lachnospira	-0.00839
Otu0176	Bacteria	Firmicutes	Clostridia	Clostridiale	Ruminococ	Ruminococ	-0.00885
Otu0275	Bacteria	Firmicutes	Clostridia	Clostridiale	Lachnospira	Lachnospira	-0.00982
Otu0244	Bacteria	Firmicutes	Clostridia	Clostridiale	Clostridiale	Clostridiale	-0.00989
Otu0021	Bacteria	Firmicutes	Clostridia	Clostridiale	Lachnospira	Lachnospira	-0.01134
Otu0067	Bacteria	Firmicutes	Clostridia	Clostridiale	Lachnospira	Lachnospira	-0.01142
Otu0122	Bacteria	Firmicutes	Clostridia	Clostridiale	Ruminococ	Ruminiclos	-0.01166
Otu0054	Bacteria	Firmicutes	Clostridia	Clostridiale	Lachnospira	Lachnospira	-0.01252
Otu0187	Bacteria	Firmicutes	Clostridia	Clostridiale	Lachnospira	Acetatifact	-0.01269
Otu0014	Bacteria	Firmicutes	Clostridia	Clostridiale	Lachnospira	Lachnospira	-0.01295
Otu0148	Bacteria	Firmicutes	Clostridia	Clostridiale	Lachnospira	Lachnospira	-0.01299
Otu0167	Bacteria	Firmicutes	Clostridia	Clostridiale	Ruminococ	Ruminiclos	-0.01305
Otu0093	Bacteria	Firmicutes	Clostridia	Clostridiale	Ruminococ	Ruminiclos	-0.01378
Otu0085	Bacteria	Firmicutes	Clostridia	Clostridiale	Lachnospira	Lachnospira	-0.01389
Otu0041	Bacteria	Firmicutes	Clostridia	Clostridiale	Lachnospira	Lachnoclos	-0.01405
Otu0140	Bacteria	Firmicutes	Clostridia	Clostridiale	Lachnospira	Lachnospira	-0.01554
Otu0152	Bacteria	Tenericutes	Mollicutes	Mollicutes_	Mollicutes_	Mollicutes_	-0.01742
Otu0033	Bacteria	Firmicutes	Clostridia	Clostridiale	Lachnospira	A2	-0.01947
Otu0101	Bacteria	Firmicutes	Clostridia	Clostridiale	Lachnospira	ASF356	-0.01955
Otu0126	Bacteria	Firmicutes	Clostridia	Clostridiale	Lachnospira	Lachnospira	-0.02153
Otu0034	Bacteria	Firmicutes	Clostridia	Clostridiale	Lachnospira	Acetatifact	-0.02155
Otu0007	Bacteria	Firmicutes	Clostridia	Clostridiale	Ruminococ	Ruminiclos	-0.02263
Otu0127	Bacteria	Firmicutes	Clostridia	Clostridiale	Lachnospira	Lachnospira	-0.02405
Otu0008	Bacteria	Firmicutes	Clostridia	Clostridiale	Lachnospira	uncultured	-0.02406
Otu0063	Bacteria	Firmicutes	Clostridia	Clostridiale	Lachnospira	Lachnospira	-0.02534
Otu0052	Bacteria	Firmicutes	Clostridia	Clostridiale	Lachnospira	Lachnospira	-0.02993
